# Teaching and Learning Computational Drug Design: Student Investigations of 3D Quantitative Structure–Activity Relationships through Web Applications

**DOI:** 10.1021/acs.jchemed.0c00117

**Published:** 2020-06-23

**Authors:** Rino Ragno, Valeria Esposito, Martina Di Mario, Stefano Masiello, Marco Viscovo, Richard D. Cramer

**Affiliations:** † Rome Center for Molecular Design, Department of Drug Chemistry and Technology, Sapienza Rome University, P. le A. Moro 5, 00185 Rome, Italy; ‡ Pharmacy and Medicine Faculty, Pharmaceutical Biotechnology Master Degree Course, Sapienza Rome University, P. le A. Moro 5, 00185 Rome, Italy; § Retired, Santa Fe, New Mexico 87501, United States

**Keywords:** Upper-Division Undergraduate, Graduate Education/Research, Continuing Education, Chemoinformatics, Interdisciplinary/Multidisciplinary, Computer-Based Learning, Molecular Modeling, Drugs/Pharmaceuticals, Medicinal Chemistry, 3D QSAR

## Abstract

The increasing use of information technology in the discovery of new molecular entities encourages the use of modern molecular-modeling tools to help teach important concepts of drug design to chemistry and pharmacy undergraduate students. In particular, statistical models such as quantitative structure–activity relationships (QSAR)—often as its 3D QSAR variant—are commonly used in the development and optimization of a leading compound. We describe how these drug discovery methods can be taught and learned by means of free and open-source web applications, specifically the online platform www.3d-qsar.com. This new suite of web applications has been integrated into a drug design teaching course, one that provides both theoretical and practical perspectives. We include the teaching protocol by which pharmaceutical biotechnology master students at Pharmacy Faculty of Sapienza Rome University are introduced to drug design. Starting with a choice among recent articles describing the potencies of a series of molecules tested against a biological target, each student is expected to build a 3D QSAR ligand-based model from their chosen publication, proceeding as follows: creating the initial data set (Py-MolEdit); generating the global minimum conformations (Py-ConfSearch); proposing a promising mutual alignment (Py-Align); and finally, building, and optimizing a robust 3D QSAR models (Py-CoMFA). These student activities also help validate these new molecular modeling tools, especially for their usability by inexperienced hands. To more fully demonstrate the effectiveness of this protocol and its tools, we include the work performed by four of these students (four of the coauthors), detailing the satisfactory 3D QSAR models they obtained. Such scientifically complete experiences by undergraduates, made possible by the efficiency of the 3D QSAR methodology, provide exposure to computational tools in the same spirit as traditional laboratory exercises. With the obsolescence of the classic Comparative Molecular Field Analysis Sybyl host, the 3dqsar web portal offers one of the few available means of performing this well-established 3D QSAR method.

## Introduction

**Figure 1:**
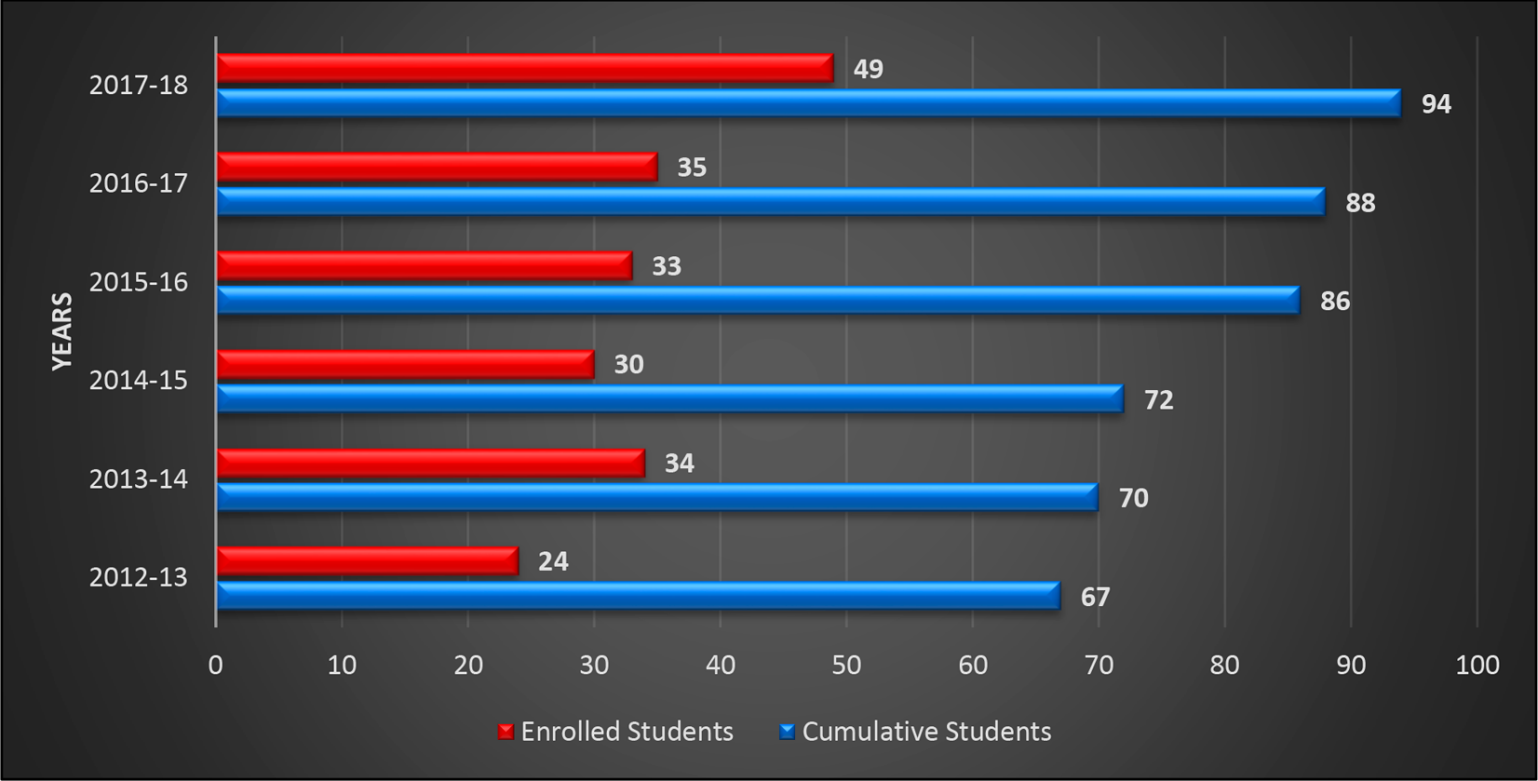
Number of SPBMD enrolled and cumulative students per year. Cumulative students are the total student in the full course (first + second years).

A basic knowledge of pharmaceutical chemistry is one fundamental goal for students in master’s degree (MD) courses such as Pharmaceutical Biotechnology (PB, PBMD) or Medicinal Chemistry (MC, MCMD) and Pharmaceutical Technology (Industrial Pharmacy Degree, PT, PTMD). To undertake these courses, students are required to have knowledge about biology, biochemistry, chemistry, pharmacology, and general pathology, normally acquired from introductory courses for medicinal chemistry during a bachelor’s degree program. Master’s degree courses usually emphasize frontal lectures delivered by teachers with the students’ evaluations being written and/or oral student exams. Only a few of them include practical training in the application of theoretical rules and learned knowledge. At Pharmacy and Medicine Faculty of Sapienza University of Rome PBMD (Sapienza PBMD, SPBMD), this traditional approach to teaching and learning is being enriched by increasing the number of practical lessons and by augmenting the final evaluation exam with the student’s multimedia presentation, given in a classroom in the presence of the teacher and other students. Although perhaps coincidentally, the number of SPBMD enrolled students has increased during the last five years (Figure 1).

More specifically, as other Italian universities^1−3^ and non-Italian universities^4−6^ have also been doing, SPBMD has been offering a drug design (DD) course, previously named medicinal chemistry or computational medicinal chemistry. At SPBMD, this DD course has gradually evolved from pure frontal to interactive lectures, increasing the students’ proficiency by exposing them to such computational resources as online databases:

- Protein Data Bank (PDB)^7^
- ChEMBL^8^
- PubChem^9^

Students also gain exposure to specialized computational chemistry software and techniques:

- Multiple linear regression (MLR)^10^
- Principal component analysis (PCA)^11^
- Partial least-squares (PLS)^12,13^
- Quantitative structure–activity relationships (QSAR)^14^
- Three-dimensional QSAR (3D QSAR)^15^
- Pharmacophore modeling (PM)^16^
- Molecular docking (MDock)^17^

### Pedagogic Aspects

This article describes how, in MOOC-like fashion (MOOC: Massive Online Open Courses^18^) with interactive lectures and online platforms, students are learning these DD methods, in particular 3D QSAR, by means of the www.3d-qsar.com portal.^19^ While doing so, students also improve their computer skills, in general and within this applied field, which can of course benefit any future career.

From a somewhat different perspective, that is, the latest thinking on the most effective approaches to education, this combination of technology usage with a socio-constructivist approach^20−22^ gives students the opportunity to benefit from real learning by doing.^23^ The curriculum combines interactive lectures and technical interaction tools that involve them in the construction of a scientific product. We are thus instantiating the so-called trialogical learning approach^24^ (TLA) and the achievement of the knowledge considered as coconstruction mediated by cultural and social artifacts and implemented at the interpersonal level, through communication and interaction with peers and experts.^25^ As in a recent definition,^26^ in the our TLA approach the students are actively collaborating in developing and creating new science work, as similarly described by Sins and Andreissen.^27^

However, the TLA approach can represent a novel challenge to a student who has been exposed only to traditional instruction and who may have not had much direct exposure to executables and files. At first the student’s feelings are uncertain. On the one hand, the volume of new concepts to be acquired, may be intimidating; yet on the other side there can be a great curiosity that drives them to explore a new way of learning. Among the 50 students that attended the course for the academic year 2017–2018, four of them were particularly interested in its educational program and were therefore invited to participate in preparing and compiling this report, by reporting their fulfillments of these assignments in this work and becoming coauthors of this publication.

Students were also asked about the difficulties encountered in performing the assignment, and their doubts and questions helped to improve the way to present some topics in the frontal lessons. Among the most frequently difficulties was the recognition of the 3-D aspects of molecules and the fact that to develop a 3D QSAR model these 3-D aspects must somehow be aligned. To help overcome some of the difficulties a blog session was created for students to pose written questions to be answered by the teacher, assistants, and also other more skilled colleagues. At the time of writing this article, this blog lists more than 300 answered questions (Italian language) and a blog session has been added to the public www.3d-qsar.com(19) (English language, the blog is accessible only after registering at the site).

### Similar Approaches

Providing students with a fuller understanding of computer-aided drug design (CADD) by the hands-on application of its methods is the goal of several educational programs that have recently been described.^28^ Of course, these programs do vary somewhat in their emphases among the large variety of CADD methods that are currently practiced. All start with the nature of the problem: the primality of 3-dimensional shape and flexibility; the multiple properties that an effective drug molecule must have the many repetitive make-and-test cycles. Hands-on instruction begins with the basic and often clunky tools of the trade, today freely available from many sources, to perform: the usage of public databases; the computer representations of chemical structure and shape, and their means of modification; the generation of canonical 3D shapes from 2D structures.

At this point, these published CADD educational programs diverge. Usually the emphasis is on docking, today’s representation, and the attempted quantification and optimization of Ehrlich’s foundational “lock-and-key” intuition, specifically of one or two ligands into a single receptor, often with a further “pharmacophoric” goal of attributing an affinity to specific interactions between ligand and receptor atoms. Our 3D QSAR approach thus rather complements docking, as it involves comparisons among many molecular structures, themselves considered as wholes.

Other noteworthy features among these educational programs include the following. Tantillo et al. agree with us in the motivational value of utilizing the exposures to these concepts and tools to complete a DD project.^28^ The Computational Structural Biology group at Molecular Modeling Group of the SIB in Lausanne describes an elegant web-based GUI’s intended to introduce DD and CADD to a very different audience, the general public, including high school teachers and students.^29^ Johnson et al.^30^ incorporated pharmacokinetic considerations in their use of lighter computational tools to strengthen a conventional medicinal chemistry course. A Brazilian group^31^ included ligand-based approaches and free computational resources in prescribing a receptor-based study of a specific target, COX-1. Sutch et al.^32^ gave special attention to pharmacokinetic approaches in the application of various web-available receptor-based tools to the caspase-3 target.

### DD Course Overview at SPBMD

The SPBMD DD course includes frontal and practical lessons. During the frontal lessons, students are taught about the history of classical and modern medicinal chemistry concepts mainly focused on structure–activity relationship (SAR), from Lipinski rules^33^ to current definitions of QSAR, pharmacophores, and molecular docking, and is also given an introduction to pharmacology, emphasizing ADMET (Adsorption, Distribution, Metabolism and Excretion–Toxicity) and thereby pharmacokinetics, with some details about the different macro-groups of drugs. Also included, in preparation for the second part of the coursework, are some theory and usage of the following molecular modeling computer programs:

- ChemAxon MarvinSketch:^34^ used to edit and draw molecules
- *UCSF Chimera*:^35^ used to visualize the structure target and in preparation for a docking study
- *AutoDock Vina*:^36^ a Chimera tool, to perform a docking study
- *OpenBabel*:^37^ used to interconvert molecular formats
- www.3d-qsar.com:^38^ a comprehensive online platform to build 3D QSAR models from scratch

Participating in practical sessions, in a multimedia room, helped the students in the use of personal computers to apply these computational chemistry tools. A series of tutorials were prepared and shared by means of the Moodle platform used in Sapienza University.^39^ Basic training in MS Windows, MAC OS, and Linux operating systems and office suites (MS Office and Libreoffice) was also available as needed.

As final test, each student was assigned a project in which all the skills acquired during both the lessons and practical sessions were to be actively applied to the design of new analogues with improved biological potency (the goal of any actual drug design project). Each student selected a scientific article starting from the current year issue of the *Journal of Medicinal Chemistry* or its ASAP articles.^40^ The selected article should include, at minimum, a list of 40 newly synthesized molecules including their affinities for a macromolecular target whose experimental structure was listed in the protein data bank.^7^ On their selected article the student should perform the following tasks:

1. Review its context in depth, considering:

a. The reason for carrying out the reported research
b. The chemical routes used to synthesize the presented compounds
c. The biology and biochemistry of the macromolecular target

2. Create models of each listed molecule and tabulate their structures, in SMILES and MOL2 formats
3. Calculate and include a first series of descriptors, in order to

a. Evaluate the molecules’ “drug-likeness” by means of the Lipinski^41^ and Veber^42^ rules and attach the calculated data
b. Examine the overall physicochemical profile of these molecules, based on the parameters that underly these “drug-like” rules, by means of PCA
c. Make QSAR models using only these same parameters, by means of MLR and PLS statistical approaches

4. Use the modeled molecules as a starting point to perform a ligand-based (LB) 3D QSAR study with the www.3d-qsar.com web applications.
5. Use the modeled molecules in a structure-based (SB) application to

a. Investigate the binding modes of the molecules by molecular docking
b. Obtain a SB molecular alignment for subsequent SB 3D QSAR study

6. Prepare a final report, which includes:

a. An electronic document describing the work and its results
b. An electronic presentation by means of MS Powerpoint or Libreoffice Impress

7. Make an oral presentation of a maximum 30–40 min

While all of these theoretical background matters are also described in the didactic material of the course, shared and freely available from the Moodle platform at Sapienza University,^39^ here the discussion will be focused only on their 3D QSAR aspects, since it seems that elsewhere 3D QSAR and analogous techniques are simply mentioned, without any practical components. Please note that our 3D QSAR facilities and experiences can provide anyone with a fresh template for teaching and learning 3D QSAR, again with no costs whatsoever and without any need to install any specialized software.

The four selected students (coauthors in this publication; V.E., M.D.M., S.M., and M.V.) were among the best students of the 2017–2018 academic year. Among their course (2017–2018), they were among the first to take the master’s degree and all of them earned the maximum degree rank cum laude. Also, their reports were among the best then reported, although with the maturation of the course and with higher reference many other students of the same academic year and many others among the following one (2018–2019) performed just as well. The high quality of the 3D QSAR models themselves was not considered a requisite to pass the exam, as not all data sets can yield satisfactory models. Instead the quality of a final presentation allowed the examining committee to evaluate the student’s understanding and performance. Only a few percent of students actually failed to pass the exam (4%) while about 16% passed with the maximum rank (30/30 cum laude), about 40% were very good (28–30/30), about 30 gained an average rank (24–27/30) and the last 10% had a low rank((18–23/30).

## A Brief Introduction to 3D QSAR

Many reviews and a few books have been published on 3D QSAR.^15,43−47^ While other 3D QSAR methods are known, with some discussed below, the field-based (FB) 3D QSARs (FB 3D QSAR) were the first introduced and are by far the most practiced, starting with the first Cramer et al. comparative molecular field analysis (CoMFA) published article,^47^ and with little subsequent enhancement of CoMFA beyond Cramer’s developments of “topomers”^48,49^ and “template CoMFA”^50−52^ as simple yet robust options for producing ligand-based (LB) alignments (in general drug design nomenclature, “Field-based” (FB) approaches are a major subset of “ligand-based” (LB) approaches, with LB in turn to be contrasted with the set of “receptor-based” or “structure-based” approaches).

To summarize the CoMFA methodology, the set of 3-D aligned molecular conformations is virtually embedded into a 3-D lattice spanning at least 5 Å, the minimum volume typically required to enclose all the molecules. At each grid point a hypothetical probe atom, typically an sp^3^ carbon atom bearing a positive charge, is placed, and then its Lennard-Jones and Coulomb law potentials with each of the atoms in every molecule are accumulated, collectively comprising the respective steric and electrostatic “interaction fields (MIF)” for each molecule. This result can be visualized as a structure/activity table or matrix with far more columns (lattice point count × 2), than rows (structures)—a severely under-constrained base for conventional model derivation! Happily, by treating this matrix as a whole rather than as a collection of independently manipulable descriptor columns, the PLS algorithm coupled with cross-validation can yield a robust mathematical model from these MIFs, although one described by a long unreadable regression equation containing thousands of terms
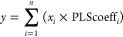
where *y* is the calculated or predicted response; *x*_*i*_ are the field values; and PLScoeff_*i*_ are the PLS correlation coefficients.

Yet the graphical representation of PLScoeff_*i*_ (after multiplication by each node point’s average value) yields very informative polyhedron whose interpretation can aid in the design of new molecules.

### Steps for the FB 3D QSAR Procedure

**Figure 2:**
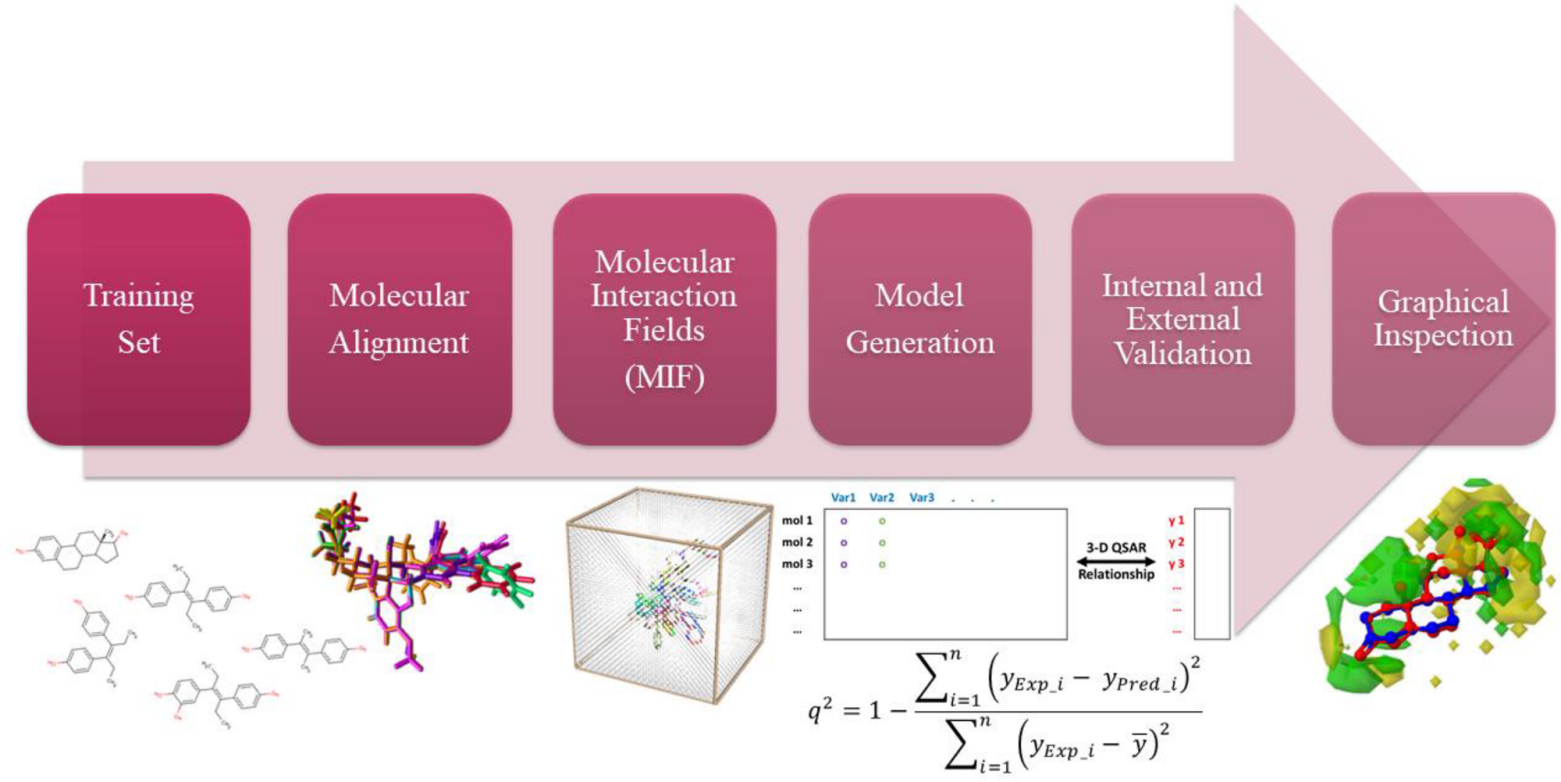
Workflow of a FB 3D QSAR procedure.

Carrying out a FB 3D QSAR model involves the following steps, as shown in Figure 2.

#### Data Set Compilation

Selection of a molecular data set with associated biological activities or properties (responses) which do not need to share a structural scaffold. It is recommended that biological activities, when as typically expressed in concentrations (IC_50_, EC_50_, *K*_i_), are expressed by the “*p* = −log() (logistic) function”, with the negative sign associating positive model coefficients with improved activities. To ensure that the differences among the activities exceed random experimental variabilities, the range of these logistic values should span at least 2 log values. Many authors are actually indicating a range of 4–5 logs to get good QSAR models, but it is not always possible to have such a range of activity.

To permit an estimate of the predictive ability of an ensuing model, the intended conclusion with CoMFA, the data set can also be split into a training (model building) and a test (model evaluation) set. To develop such a reliable model a minimum number of 15–20 molecules for the training set is desirable.

#### Generation of 3-D Conformations

If the data set molecules are available only in SMILES format, their “flat” representations must first be converted into 3-D conformations. Appropriate tools include graphical sketch-based (UCSF Chimera^35^) or command line methods (OpenBabel^53^) or free web services (ochem.eu).^54^

#### Definition of Alignment Rules

This is the most critical step. Its goal is to superimpose (align) each of the molecules so that the differences among their atoms’ identities and positions represent the resulting field differences in a way that then yields a satisfactory CoMFA model. Different alignment strategies can be adopted.^55^ Many articles have reported several approaches to obtain the aligned training set through automatic alignment programs^56^ or by atom-by-atom superposition of the maximum common substructure^57^ or by using pharmacophore modeling.^58^ One of us (Cramer) has found that alignment rules, such as “topomers” and “template CoMFA”, which prefer the field differences intentionally introduced by chemical synthesis to those imposed by physicochemical realities, often easily generate remarkably powerful and versatile 3D-QSAR models. However, with the demise of classical CoMFA’s Sybyl host but the continuance of relevant patents, these alignment methods are unfortunately available only as crude, unsupported, and almost unvalidated open source codes that also require the acquisition and installation of underlying commercial software.

#### Calculation of MIF

The aligned molecules are virtually placed in a grid of proper dimension and the MIF are calculated.

#### Model Generation

At this point, all the data needed to run the PLS and generate the models for a given number of extracted principal components have been assembled. The goodness of the models is evaluated through the squared correlation coefficient *r*^2^ calculated by
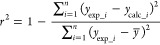


#### Model Validation

The best model is selected by means of

- Robustness: Cross-validation that indicates the most robust model on the basis of an internal predictive coefficient called *q*^2^ evaluated by the following equation:

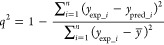

- Lack of Chance Correlation: Even a robust model could have been obtained by chance. A further test is the “Y-scrambling” (Y–S) method, by randomly reassigning the experimental potencies to the compounds. Upon rederivation of this now clearly invalid model, the obtained *r*_Y–S_^2^ and *q*_Y–S_^2^ values should be always lower than those of the original model.
- Predictive Ability: In the case of availability of additional molecules external to this model-building set (perhaps by subsequent synthesis), it is the usual practice in CoMFA to evaluate the predictive ability of its final model, by calculating the standard deviation error of prediction (SDEP), and possibly computing an *r*_pred_^2^ (*r*^2^ calculated using the predicted and experimental responses of the test set). The calculation of SDEP can be easily achieved with the standard deviation equation:

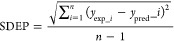


#### Graphical Interpretation

A series of plots are generated to inspect and describe the model and design new derivatives on the basis of the observation of the plots superimposed on the training set molecules.

### Field-Based Instructional Resources

As mentioned above, 3D QSAR was introduced by Cramer et al. in 1988,^47^ with the name of CoMFA as an acronym for “Comparative Molecular Field Analysis”. Nevertheless, despite the application of 3D QSAR as CoMFA in tens of thousands of articles and many books, we can find no current evidence of any practical presentation within a DD academic MD course. CoMFA had been available only in the Tripos Suites (aka Sybyl), in a very friendly form so easy to use that with a few “mouse clicks” almost any user was able to develop their own 3D QSAR model, and was well documented in the CoMFA manual. Nevertheless some IT knowledge and costs were required to obtain, install and run the software. Although other 3D QSAR software appeared on the scene, the original CoMFA patent prevented its commercialization by any other software house until its expiration in 2011. Possibly the further development of 3D QSAR was inhibited; 4D QSAR,^59^ 5D QSAR,^60^ and 6D QSAR^61^ appeared but few scientific applications followed. However, the Cresset Group now commercially offers a field-based nonlattice 3D QSAR package.^62^ In 1994, Cruciani et al. introduced GOLPE,^63^ a chemometric tool that in combination with Goodford’s GRID provided highly reliable 3D QSAR models. In 2011, after the CoMFA patent expired, Open3DQSAR,^64^ was announced, the first free and open source program that with the companion softwares Open3DALIGN^65^ and Open3DGRID (open3DGRID.sourceforge.net) provided all the steps in 3D QSAR. However, a web-based implementation of Open3DQSAR in a Web site^66^ lacked model building and graphical analysis. In 2012, 3D-QSAutogrid/R,^67^ a second open source software based 3D QSAR procedure, was reported in which the MIFs were calculate by means of the AutoDock suite AutoGrid program^68^ and the statistical calculations (PLS, cross-validation and Y-scrambling) were performed by means of the R-CRAN pls package.^69^ The only other CoMFA source, an open source Open Eye-based code, was mentioned above.

Thus, although these other tools for building other types of FB 3D QSAR models exist, it is evident that, today, well-validated classic CoMFA 3D QSAR models can easily be created at no cost with Open3DQSAR and via the readily accessible and www.3d-qsar.com portal.^19^

## Developing 3D QSAR Models by Means of the QSAR Web Portal

### Overview of the Web Portal Procedure

Here is an overview of this portal’s workflow, serving also as background for the following summaries of the four student projects. The scientific and technical rationales underlying each of its six successive stages have already been summarized.

#### Data Set Compilation through the Web Portal

At first, an empty data set container, a sort of work folder or virtual spreadsheet, is prepared by means of the Py-MolEdit web application. Molecules may be added to this data set in several different ways. For these projects 3D structures were created with MarvinSketch and uploaded to the data set container using the “Add Multiple Molecules” command. A separately prepared file added biological activities to these data set molecules. (See the Py-MolEdit tutorial link in the Supporting Information.)

#### Generation of 3-D Conformations through the Web Portal

A ligand-based (LB) conformation analysis was performed with the Py-ConfSearch module on all of the molecules in this data set. Its goal is to explore the conformational space of each molecule and provide an idea of their flexibility, with a family of conformations being saved for every flexible molecule. (See the Py-ConfSearch tutorial link in the Supporting Information.)

#### Definition of Alignment Rules through the Web Portal

Single conformations, selected from the conformation family for each of the molecules are aligned by means of the Py-Align application, forming one training set. The Py-Align application provides 16 alignment approaches, with the expectation that many different training sets, conformational analysis and alignment couplex, will be considered from each data set. (See the Py-Align tutorial link in the Supporting Information.)

#### Calculation of MIF Fields and Model Generation through the Web Portal

For each such aligned training set, a 3D QSAR model can be generated using the Py-CoMFA module^70^ with the default settings, which can then be further explored by varying the probe type, the grid spacing, the min-max cutoff energy and the minimum sigma. To evaluate model robustness, cross-validation is done as the model is built. Other tests for chance correlation, as discussed above, can be performed on the completed model. (See the Py-CoMFA tutorial link in the Supporting Information.)

#### Model Validation and Prediction through the Web Portal

Since the main goal of any 3D QSAR model is to predict unknown molecules activities by means of their structures and the alignment rules, an external test set is usually prepared along with the training set. The aim of external validation is to verify the predictive capacity of the model. Including this capability can be made part of the derivation of the 3D QSAR model, by random selection from the test set molecules in the data set container.

#### Graphical Interpretation through the Web Portal

Still within the Py-CoMFA application, once the model has been built and validated the standard CoMFA 3-D map can be created and inspected directly through the web browser without the use of any supplementary software. The polyhedron images can generated for an individual molecule’s MIF as well as for the entire 3D QSAR model.

### Protocol Most Commonly Followed by the Students

#### Target Assignment

As described above (see also DD course overview at SPBMD) the students were each assigned a *J. Med. Chem*.^71^ article describing more than 40 molecules for a given biological target. The four targets chosen were: indoleamine 2,3-dioxygenase 1 (IDO1),^72,73^ tyrosine–protein phosphatase nonreceptor type II (SHP-2),^74,75^ interleukin-1 receptor associated kinase-4 (IRAK4),^76,77^ and bromodomain-containing protein 4 (BRD4).^78^

#### General Procedures for Initial Molecular Modeling of Ligands

All of the molecules listed in the target articles were drawn and edited through ChemAxon, MarvinSketch, or the Py-MolEdit web application available on the Web site www.3d-qsar.com.^19^ (For use, see the Supporting Information.) With ChemAxon, the molecules must be downloaded from another Web site, and the direct editing on Py-MolEdit saves the molecule in the database. Conformation analysis was performed on each of the four completed data sets (Py-ConfSearch), specifically using the Balloon^79^ method, and an arbitrary number of conformations for each molecule, specifically 70, were aligned by Py-Align. Py-Align provides 16 possibilities from a training set as templates for alignment, such as the most active, the less active, or the most flexible. Once the template is selected, Py-Align provides several options for automatic alignment; for most projects the option chosen was ShaEP^56^ with the default settings. From the result of each training set alignment, a 3D QSAR model was generated.

#### General Procedure for the 3D QSAR Models’ Development

The 3D QSAR Ligand-Based approach was performed with the same default settings for all listed targets. Therefore, for each one a conformational analysis was carried out, various alignment rules were applied and finally, several models were generated. Then, these models were optimized, modifying the settings as following:

- *Probes atom:* C.3, C.2, C.cat, O.3, N.3, H
- *Grid spacing*: range 1–3 with 0.1 unit of difference
- *Grid extension*: range 5–10 with 1 unit of difference
- *Min/max cutoff energy*: range 20–40 with 5 units of difference
- *Minimum Sigma*: range 0.05–2 with 0.05 units of difference

A total of about 80 3D QSAR models were built for each target, and for the one yielding the highest *r*^2^ and *q*^2^ values, both steric and electrostatic contours maps were generated and analyzed through the graphical plots implemented within www.3d-qsar.com.^19^

## Student Work Examples

The students applied the above protocol to their selected *J. Med. Chem*. articles to generate ligand-based aligned FB 3D QSAR models by means of the www.3d-qsar.com portal^19^ (Table 1). Considering all four of the projects, a total of 194 inhibitors were modeled and more than 320 3D QSAR models were developed and internally validated for fitting, robustness, and lack of chance correlation. External validations were also performed in three of the four projects, yielding low errors of predictions (see the Supporting Information). Each of the four final models were further analyzed by means of classical CoMFA contour maps revealing the possibility for future drug design.

**Table 1:** Comparison of 3D QSAR Models Metrics^a^

						*q*^2^		SDEP^d^			*q*^2^ YS		settings				
target	*N*	fields	ONPC^b^	*r*^2^	SDEC^c^	LOO^e^	LSO^f^	LOO^e^	LSO^f^	*r*^2^ YS	LOO^e^	LSO^f^	PA^g^	GS^h^	GE^i^	MS^j^	C^k^
IDO1	51	Ste^l^	2	0.54	0.77	–0.06	–0.06	1.17	1.17	0.52	–0.27	–0.28	C.2	1.312	5	0.05	15
		Ele^m^	8	0.98	0.13	0.47	0.40	0.84	0.87	0.88	–1.45	–0.94					
		Both^n^	8	0.98	0.12	0.15	0.19	1.04	1.02	0.94	–0.36	–0.42					
SHP-2	40	Ste^l^	4	0.84	0.31	0.41	0.41	0.61	0.61	0.76	–0.67	–0.46	H	2.200	10	1.50	25
		Ele^m^	1	0.18	0.71	0.12	0.12	0.74	0.74	0.05	–0.12	–0.10					
		Both^n^	4	0.78	0.36	0.27	0.29	0.67	0.66	0.61	–0.61	–0.51					
IRAK4	58	Ste^l^	6	0.98	0.13	0.29	0.31	0.94	0.93	0.96	–0.30	–0.29	O.3	1.000	5	2.00	25
		Ele^m^	4	0.83	0.46	0.21	0.26	0.99	0.96	0.62	–0.36	–0.47					
		Both^n^	6	0.97	0.16	0.44	0.45	0.83	0.83	0.93	–0.30	–0.27					
BRD4	45	Ste^l^	2	0.69	0.40	0.28	0.31	0.61	0.60	0.55	–0.64	–0.39	H	2.200	5	0.05	25
		Ele^m^	2	0.31	0.60	0.04	0.08	0.71	0.69	0.22	–0.39	–0.23					
		Both^n^	4	0.88	0.24	0.54	0.54	0.49	0.48	0.74	–0.67	–0.36					

a Note: For further details, see the Supporting Information.

b ONPC: Optimal number of principal components.

c SDEP: Cross-validated standard deviation error prediction.

d SDEC: Standard deviation error calculation.

e LOO: Leave-one-out.

f LSO: Leave-some-out.

g PA: Probe atom.

h GS: Grid step.

i GE: Grid extension.

j MS: Minimum sigma.

k C: Max/min energy of cutoff value.

l Ste: Steric MIF.

m Ele: Electrostatic MIF.

n Both: Steric and electrostatic fields.

## Conclusions

In this publication, we believe we have demonstrated the effectiveness and the convenience of the free Web site platform www.3d-qsar.com as a tool to teach 3D QSAR in DD courses, including the difficult underlying chemical concepts. Our experiences suggest that the 3D QSAR approach is quite appropriate for undergraduate students of pharmaceutical biotechnology, and more generally as a part of bachelor, master, and Ph.D. degree programs, and perhaps even within high school courses. Particularly relevant evidence for this view could be the work performed by the four students from the SPBMD course during the academic year 2017–2018, who constructively criticized and then employed four modules of the www.3d-qsar.com website^19^ (Py-MolEdit, Py-ConfSearch, Py-Align, Py-CoMFA) to complete a ligand-based 3D QSAR study. This experience helped these students to obtain a high score in the drug design exam, increasing their biological computational skills, and encouraging them to continue these kind of studies.

To also provide students with some experience in receptor-based methodogies, since the targets of the four publications used for this study are available in crystallized form in the online database PDB (protein data bank),^7^ another study will describe the use of these targets to generate alternative molecular alignments for 3D QSAR. Also, video tutorial are planned for release soon to provide even better guides to develop 3D QSAR models.

In conclusion, beyond the value of the students’ models themselves, their work clearly demonstrates that by using the protocol we describe they learned many skills and perspectives in computational medicinal chemistry, as was the main objective of the course.

## Supporting Information Available

Details of student work examples with tutorial links for interacting at the 3D QSAR Web site (PDF)
