# Supplementary material for: Teaching and Learning Computational Drug Design: Student Investigations of 3D Quantitative Structure–Activity Relationships through Web Applications
Source: J Chem Educ. 2020 Jun 23;97(7):1922–30. doi: 10.1021/acs.jchemed.0c00117 (PMC8008382; doi:10.1021/acs.jchemed.0c00117)
Supplement: Supplementary file 1 — ed0c00117_si_001.pdf [file ed0c00117_si_001.pdf]

Supplementary Information

Teaching and Learning Computational Drug Design. Three-Dimensional Quantitative Structure-Activity Relationships (3D-QSARs) Through Web Applications

Rino Ragno,[ς,¥,*] Valeria Esposito,[¥,†] Martina Di Mario,[¥,†] Stefano Masiello,[¥,†] Marco Viscovo[¥,†] and Richard D. Cramer[§]

[ς]Rome Center for Molecular Design, Department of Drug Chemistry and Technology, Sapienza Rome University, P. le A. Moro 5, 00185 Rome, Italy
[¥]Pharmacy and Medicine Faculty, Pharmaceutical Biotechnology Master Degree Course, Sapienza Rome University, P. le A. Moro 5, 00185 Rome, Italy
[§]Retired, Santa Fe, NM, USA

1. Students works [V.E.]: Indoleamine 2,3-dioxygenase 1 (IDO1)

In this section is described the development of FB LB 3-D QSAR model based on the molecules described by Tianwei Weng et al[50] and Ute F. Röhrig et al [51]. The molecules were evaluated as inhibitors of IDO1.

Overview of the biological target and connected pathology. IDO1 (Figure SI1) is a monomeric oxidase (45 kDa) containing an active heme with a Fe^{+2} ion. Structurally, the active site of IDO1 comprises two lipophilic regions: a pocket immediately above the heme group, site of the catalytic tryptophan (pocket A, in orange), and a lipophilic binding area (pocket B, in green). Pocket B is composed by several key residues, such as Arg231, Phe226 and Phe227, which were recognized as fundamental for IDO1 activity.[50]

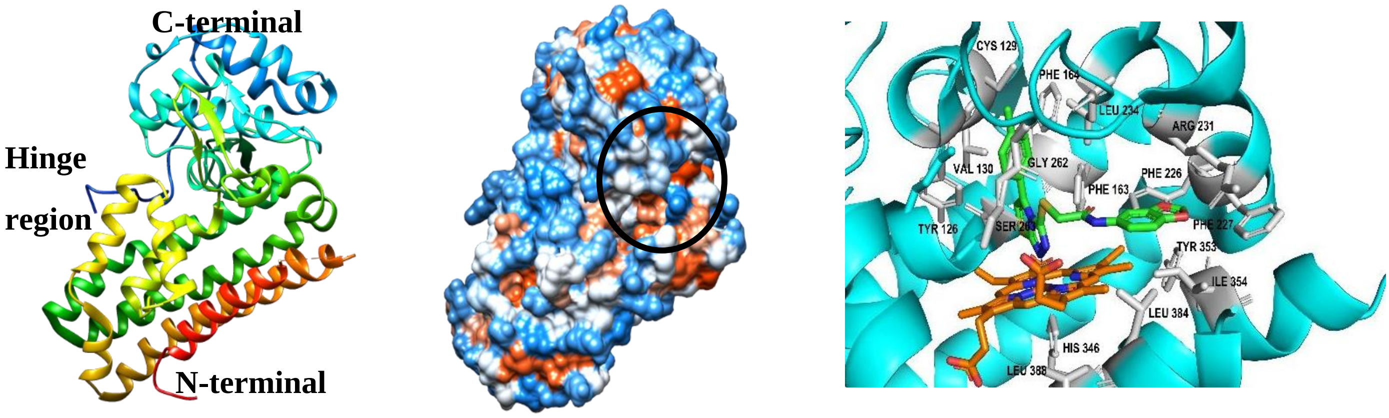

A B C

Figure SI1. Cartoon representation of IDO1 3D structure. (A): There is a C-terminal lobe (blue and light blue) linked by a hinge region with the N-terminal lobe (green, yellow, orange and red). (B): Surface representation of hydrophobic and hydrophilic sites of the protein. The black circle indicates the core. (C): Cartoon, stick and surface representation of the interaction between a competitive inhibitor (orange) and the binding site. For figure generation, the PDB entry code 4PK5 was used.

Cancer immunotherapy is currently entering an exciting new era. Among attractive immunotherapy approaches, immune checkpoint therapy which targets regulatory pathways to enhance the antitumor immune responses of T cells has led to important clinical advances in cancer. A central role in immune escape has been attributed to the kynurenine pathway of tryptophan (TRP) metabolism, which leads to the depletion of tryptophan and to the production of kynurenine metabolites, both responsible for local immunosuppression.[51] IDO1, which catalyzes the initial and rate-limiting step of the kynurenine pathway, is expressed by tumour cells to escape a potentially effective immune response, and high IDO1 expression is associated with poor prognosis in a variety of cancer types.

In vitro and in vivo studies demonstrated that IDO1 inhibitor administration improved the efficacy of therapeutic vaccination, chemotherapy, or radiation therapy. Physiologically, IDO1 role is to prevent autoimmunity phenomena and is therefore involved in peripheral immune tolerance. The classical concept proposes that tumour cells express high levels of IDO1, whose enzymatic activity determines the depletion of TRP in the local microenvironment and the consequent inhibition of T cell responses. T cells, in fact, detect low levels of TRP through unloaded tRNAs and subsequently initiated a lack of amino acid response resulting in cell cycle arrest and cell death. It has also been shown that IDO1 expression is induced by tumour necrosis factor alpha (TNF-α) and other inflammatory mediators.[50] Therefore, IDO1 could be induced as a consequence of the initial inflammatory response to the tumour. Furthermore, several studies have proposed that the immunosuppression by the degradation of TRP is not only a consequence of the lowering of local TRP levels but also the accumulation of high levels of TRP metabolites. This idea was supported by studies demonstrating that T cell responses are inhibited by TRP metabolites due to the binding of the kynurenine metabolite (KYN) with the aryl hydrocarbon receptor (AHR) which involves modulation of the immune response and the IDO1 activity of.[59] [50]

Training set composition. The training set comprised 51 molecules (Table SI1)[50] compiled from two recent articles [50-51] reporting inhibitor potencies (IC_{50}) spanning over 5 logs units (pIC_{50}: min 3.400, max 8.770). The molecules belong to different scaffolds as depicted in Table SI2.

Table SI1. IDO1 training set composition. The table shows the experimental activity of each molecule and the predicted activity of the model for each molecule, with the relative error of fitting.

Mol ID	Experimental	Predicted	Error
**IDO1_1**	7.140	6.953	0.187
**IDO1_2**	7.890	5.410	2.480
**IDO1_3**	6.390	6.464	-0.074
**IDO1_4**	8.770	6.603	2.167
**IDO1_5**	4.890	4.919	-0.029
**IDO1_6**	4.340	5.325	-0.985
**IDO1_7**	5.150	6.026	-0.876
**IDO1_8**	3.420	4.026	-0.606
**IDO1_9**	3.400	5.513	-2.113
**IDO1_10**	4.320	4.364	-0.044
**IDO1_11**	5.320	5.208	0.112
**IDO1_12**	5.120	5.577	-0.457
**IDO1_13**	5.110	5.721	-0.611
**IDO1_14**	5.520	6.625	-1.105
**IDO1_15**	7.110	5.667	1.443
**IDO1_16**	5.820	5.587	0.233
**IDO1_17**	7.170	7.903	-0.733
**IDO1_18**	7.230	6.786	0.444
**IDO1_19**	5.150	5.798	-0.648
**IDO1_20**	6.270	5.894	0.376
**IDO1_21**	6.780	6.687	0.093
**IDO1_22**	6.890	6.342	0.548
**IDO1_23**	7.450	6.302	1.148
**IDO1_24**	5.350	5.608	-0.258
**IDO1_25**	5.480	5.436	0.044
**IDO1_26**	4.920	5.979	-1.059
**IDO1_27**	5.120	5.223	-0.103
**IDO1_28**	5.390	5.367	0.023
**IDO1_29**	6.570	6.040	0.530
**IDO1_30**	5.100	4.491	0.609
**IDO1_31**	5.550	5.207	0.343
**IDO1_32**	5.280	4.865	0.415
**IDO1_33**	4.080	5.148	-1.068
**IDO1_34**	5.220	5.364	-0.144
**IDO1_35**	6.050	6.148	-0.098
**IDO1_36**	6.620	5.573	1.047
**IDO1_37**	6.960	5.459	1.501
**IDO1_38**	6.700	6.167	0.533
**IDO1_39**	4.680	5.523	-0.843
**IDO1_40**	5.000	5.916	-0.916
**IDO1_41**	4.820	6.612	-1.792

**IDO1_42**	5.110	5.027	0.083
**IDO1_43**	6.600	6.018	0.582
**IDO1_44**	5.770	5.467	0.303
**IDO1_45**	5.660	4.883	0.777
**IDO1_46**	5.920	5.731	0.189
**IDO1_47**	4.300	4.511	-0.211
**IDO1_48**	4.080	5.428	-1.348
**IDO1_49**	6.480	6.196	0.284
**IDO1_50**	4.920	5.735	-0.815
**IDO1_51**	7.220	7.596	-0.376

Table SI2. IDs, SMILES and 2-D structures of the IDO1 datasets.[1-2]

ID	SMILES	2-D Chemical Structure
**IDO1_01**	NS(=O)(=O)NCCNC1=NON=C1\C(NC1=CC(Br)=C(F)C=C1)=N\O	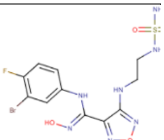
**IDO1_02**	[H][C@@]1(CC[C@H](O)CC1)[C@@H](O)C[C@@H]1N2C=NC=C2C2=C1C(F)=CC=C2	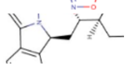
**IDO1_03**	FC1=CC2=C(NC=C2C2CC(=O)NC2=O)C=C1	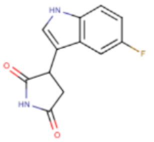
**IDO1_04**	[H][C@@]1(CC[C@]([H])(CC1)C1=C2C=C(F)C=CC2=NC=C1)[C@@H](C)C(=O)NC1=CC=C(Cl)C=C1	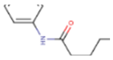
**IDO1_05**	FC(F)(F)OC1=CC2=C(NC(=C2)C(=O)CC2=CC=CN=C2)C=C1	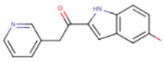
**IDO1_06**	[H][C@@]12CC3=C(NC4=C3C=CC(Br)=C4)[C@@H](C)N1C(=S)N(C)C2=O	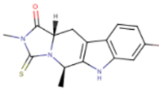
**IDO1_07**	COC1=CC(SC2=CNC3=C2C=C(Cl)C=C3)=CC(OC)=C1	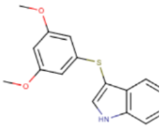
**IDO1_08**	CN1C=C(CC(N)C(O)=O)C2=C1C=CC=C2	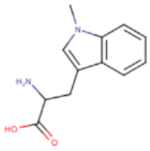
**IDO1_09**	CN1C=C(C[C@H](N)C(O)=O)C2=C1C=CC=C2	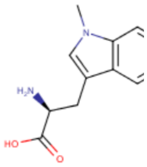
**IDO1_10**	N1C=NC=C1C1=CC=CC=C1	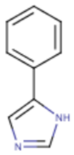
**IDO1_11**	OC1=CC=CC=C1C1=CN=CN1	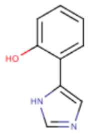

**IDO1_12**	SC1=CC=CC(=C1)C1=CN=CN1	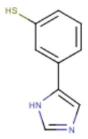
**IDO1_13**	SC1=CC=C(C=C1)C1=CN=CN1	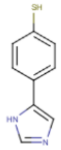
**IDO1_14**	CC1=CC=C(C=C1)C1=CSC2=NN=C(SCC(=O)NC3=CC=C4OCOC4=C3)N12	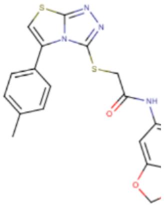
**IDO1_15**	BrC1=CC=C(C=C1)C1=CSC2=NC=C(CNC(=O)NC3=CC=C(C=C3)C#N)N12	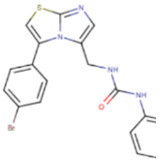
**IDO1_16**	NC1=NON=C1\C(NCC1=CC=CC=C1)=N\O	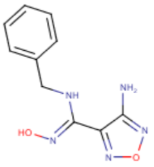
**IDO1_17**	NC1=NON=C1\C(NC1=CC(Cl)=C(F)C=C1)=N\O	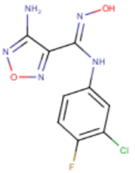
**IDO1_18**	O\N=C(\CC1=CC=C(F)C(F)=C1)NC1=CC=C(C2=NON=C12)N(=O)=O	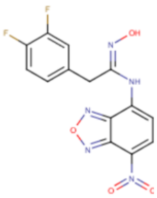
**IDO1_19**	O=C1C2=NC3=C(C=CC=C3)C(=O)N2C2=CC=CC=C12	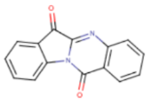
**IDO1_20**	FC1=CC=C2N3C(=NC4=C(C=CC=C4)C3=O)C(=O)C2=C1	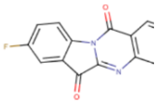
**IDO1_21**	O=S(=O)(CCC1=CC=CC=C1)NNC1=CC=CC=C1	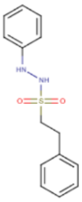
**IDO1_22**	CC(=O)NC1=CC=C(C=C1)S(=O)(=O)NNC1=CC=C(Br)C=C1	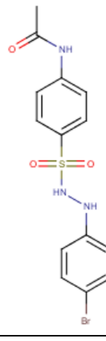

**IDO1_23**	BrC1=CC=C(NNS(=O)(=O)C2=CC3=C(NC(=O)C3)C=C2)C=C1	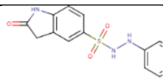
**IDO1_24**	ClC1=CC=C(SCNC2=CC=CC=C2Cl)C=C1	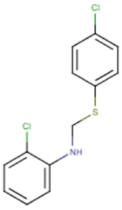
**IDO1_25**	OC1=CC=CC=C1C1=NC2=C(N1)C1=C(C=CC=C1)C1=C2C=CC=C1	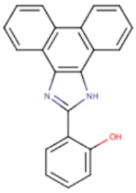
**IDO1_26**	CC1=CC=C(C=C1)S(=O)C1=CC=C(C2=NON=C12)[N+]([O-])=O	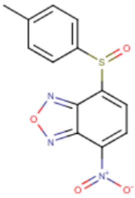
**IDO1_27**	ClC1=CC=C(CSC2=NC(=O)NC=C2)C(Cl)=C1	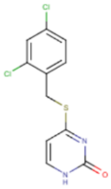
**IDO1_28**	COC1=C(OC)C=C2C(=C1)C(=O)C(C1=CC=CC=C1)=[N+]2[O-]	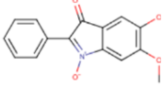
**IDO1_29**	NNC1=CC=CC=C1	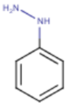
**IDO1_30**	NNC1=NC2=C(S1)C=CC=C2	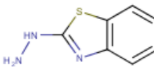
**IDO1_31**	OC1=CC=CC(=C1)C1=CSC(N\N=C2/CC(=O)C3=C2C=CC=C3)=N1	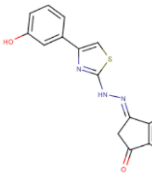
**IDO1_32**	COC1=C(OC)C=C(C=C1)C1=CSC(N\N=C2/CC(=O)C3=C2C=CC=C3)=N1	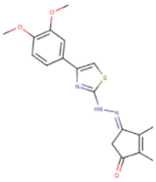
**IDO1_33**	N1N=NC=C1C1=CC=CC=C1	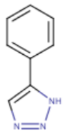
**IDO1_34**	COC1=CC=CC2=C1C(=O)C1=C(C[C@H](CN3C=C(N=N3)C3=CC=CC=C3)O[C@@H]1C)C2=O	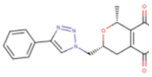
**IDO1_35**	COC1=CC=C(C=C1)C1=CNC2=C1C1=NCCC3=CNC(=C13)C2=O	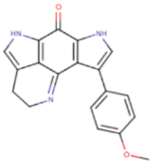
**IDO1_36**	COC1=CC(=O)C2=C(C(CO)=C(C)O2)C1=O	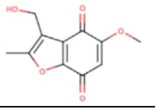

**IDO1_37**	C[C@@]12CCCCC1=C(O)C(=O)C1=C2C=C2C(=O)C3=C(C(=O)C2=C1)S(=O)(=O)CCN3	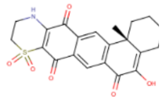
**IDO1_38**	CC(C)\N=C1/C=C(C)C(=O)C2=CC=CC=C12	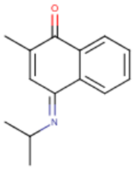
**IDO1_39**	CN1C([C@H](O)C2=CC=CC=C2)C2=NC3=CC=CC=C3C(=O)N2C2=CC=CC=C2C1=O	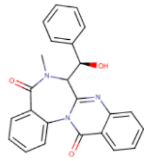
**IDO1_40**	[H][C@@]12CC[C@@]3(C)[C@@H]4CC5=C(C)C=CC(C(O)C(O)=O)=C5[C@@]4(C)CC[C@]3(C)[C@@]1(C)CC[C@@H]1O[C@@]3(C)CCCC(C)(C)[C@@H]3CC[C@@]21C	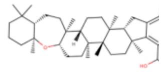
**IDO1_41**	COC1=C(C(O)=O)C(C)=C(C)C(OC(=O)C2=C(C)C(C)=C(OC(=O)C3=C(C)C=C(O)C(C)=C3O)C(C)=C2OC)=C1	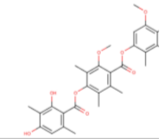
**IDO1_42**	CC1(C)CCC[C@@]2(C)[C@H]1CC[C@]1(C=O)[C@@H]2CC=C(CC1O)C=O	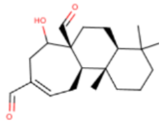
**IDO1_43**	SCC1=CC=CC=C1	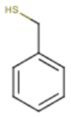
**IDO1_44**	NC(=N)SCC1=CC=C(Cl)C=C1	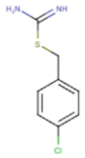
**IDO1_45**	NNC(=S)NC1=CC=C(C=C1)C#N	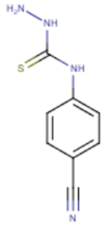
**IDO1_46**	NC1=CC=C2SC3=NCCN3C2=C1	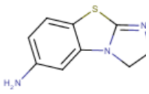
**IDO1_47**	ClC1=CC(Cl)=C(C=C1)C(CN1C=CN=C1)OCC1=CN=CC=C1	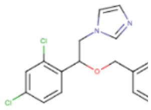
**IDO1_48**	OC1=CC=C(Cl)C=C1C1=CN=NN1	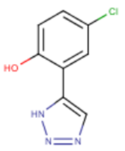
**IDO1_49**	CCOC1=NC2=C(N1CC1=CC=C(C=C1)C1=CC=CC=C1C1=NNN=N1)C(=CC=C2)C(=O)OC(C)OC(=O)OC1CCCCC1	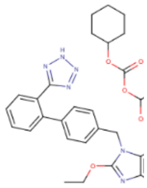

**IDO1_50**	OC(=O)C1=CC=C(C=C1)S(=O)(=O)NNC1=CC=C(Br)C=C1	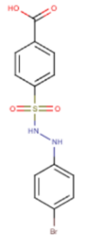
**IDO1_52***	COC1=C(SC2=CNC3=CC=C(Cl)C=C23)C=CC=C1	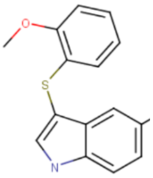
**IDO1_53***	NC1=NON=C1\C(NC1=CC=C(F)C(Cl)=C1)=N\O	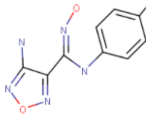
**IDO1_54***	OCCNC1=NON=C1\C(NC1=CC=C(F)C(Br)=C1)=N\O	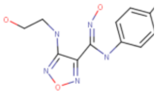
**IDO1_55***	NS(=O)(=O)NCCNC1=NON=C1\C(NC1=CC=C(F)C(Cl)=C1)=N\O	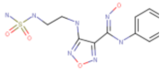
**IDO1_56***	CC1=CC=C2N3C(=NC4=C(C=CC=C4)C3=O)C(=O)C2=C1	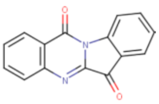
**IDO1_57***	FC1=CC=C2N3C(=NC4=C(C=C(F)C=C4)C3=O)C(=O)C2=C1	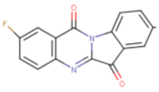
**IDO1_58***	O=C1C2=NC3=C(C=CC=C3)C(=O)N2C2=CC=C(C=C12)N(=O)=O	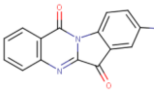
**IDO1_59***	OC1=CC=C(F)C=C1C1=NNC=C1	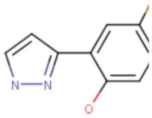
**IDO1_60***	OC1=C(CC2=C(O)C=CC(Cl)=C2)C=C(Cl)C=C1	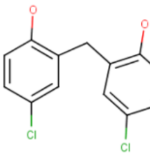
**IDO1_61***	COC1=CC2=CC=CN=C2C(NC(C)CCCN)=C1	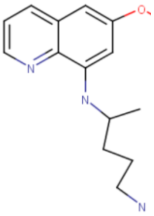
**IDO1_62***	O=C(CC1N2C=NC=C2C2=C1C=CC=C2)C1CCCCC1	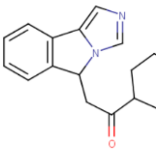
**IDO1_63***	FC1=CC=CC2=C1C(CC(=O)C1CCCCC1)N1C=NC=C21	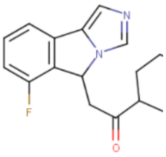

* **Test Set**

3-D QSAR model building. Through the Balloon in the Py-ConfSearch module, 70 conformations for each training set molecules were calculated and aligned by means of Shaep (Py-Align) (Figure SI2). Multiple alignments were carried out by means of the different templates as implemented in the web application (i.e. the most/least polar molecule, the most/least rigid molecule, and so on). All the 16 available alignment rules were applied leading to 16 3-D QSAR models built with default settings. Further parameters were also changed to optimize the best default model by varying the probe type, the grid spacing, the min-max cutoff energy and the minimum sigma. (Tables SI3 -SI 4)

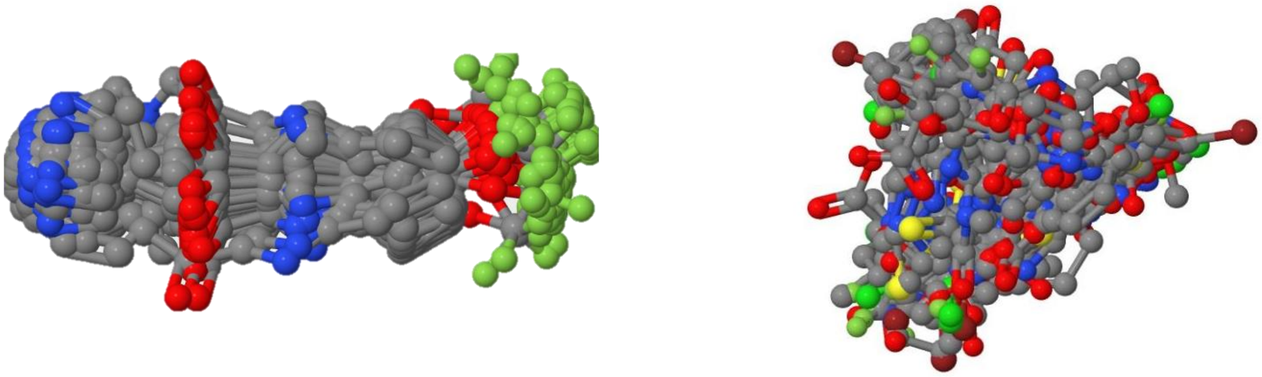

A **B**

Figure SI2. IDO1 alignment rules. **(A)** example of conformations obtained for the compound IDO1_5. **(B)** Training set aligned on the best alignment rule, most polar.

The best 3-D QSAR model was obtained aligning the training set molecules using the "most polar" (highest LogP value) molecule as a template and using as probe atom a sp2 carbon atom (C.2). The model's building settings were grid spacing of 1.312 Å, obtained through optimizations (Tables SI3 and SI4), minimum sigma 0.05, grid extension 5 Å, and cutoff energy of 15 kcal/mol. On the best model, both LOO and LSO validation were performed to evaluate the model robustness. Finally, the Y-S procedure was carried out, to verify any lack of chance correlation. The r^2_{Y-S} and q^2_{Y-S} values were always lower than those obtained on this best model, suggesting that this model was not a chance correlation (Table SI5 and Figure SI3).

Table SI3. IDO1 3-D QSAR coefficients obtained for the different alignment rules.

Alignment Rules	Probe Atom	q^2 STE	q^2 ELE	q^2 BOTH	Max ONPC	Grid Spacing	Grid Extension	CutOff	Min. Sigma	CV Type
**Least Active**	C.3	-0.214 (1)	0.194 (4)	-0.030 (4)	8	1.0	5	30.0	0.05	LOO
**Most Active**	C.3	-0.186 (1)	0.144 (5)	0.047 (4)	8	1.0	5	30.0	0.05	LOO
**Heaviest**	N.3	-0.319 (2)	-0.044 (1)	0.028 (6)	8	1.0	5	30.0	0.05	LOO
**Longest**	C.3	-0.009 (2)	-0.049 (1)	-0.032 (1)	8	1.0	5	30.0	0.05	LOO
**Most Flexible**	H	0.064 (2)	-0.054 (1)	-0.044 (1)	8	1.0	5	30.0	0.05	LOO
**Most Rigid**	H	0.140 (6)	-0.013 (4)	0.058 (6)	8	1.0	5	30.0	0.05	LOO
**Least Polar**	C.cat	-0.032 (4)	-0.045 (1)	-0.036 (1)	8	1.0	5	30.0	0.05	LOO
**Most Polar**	C.2	-0.041 (2)	0.262 (8)	0.158 (3)	8	1.0	5	30.0	0.05	LOO
**Highest MR**	C.3	-0.326 (2)	-0.044 (1)	0.027 (6)	8	1.0	5	30.0	0.05	LOO
**Lowest MR**	C.cat	-0.120 (8)	-0.042 (1)	-0.035 (1)	8	1.0	5	30.0	0.05	LOO
**Highest HA**	C.2	-0.326 (2)	-0.044 (1)	0.019 (6)	8	1.0	5	30.0	0.05	LOO
**Lowest HA**	C.2	0.075 (6)	-0.050 (1)	-0.003 (8)	8	1.0	5	30.0	0.05	LOO
**Highest HD**	O.3	-0.041 (2)	0.247 (8)	0.163 (3)	8	1.0	5	30.0	0.05	LOO
**Lowest HD**	N.3	-0.068 (4)	0.125 (2)	0.039 (2)	8	1.0	5	30.0	0.05	LOO
**Highest LogP**	C.2	-0.138 (2)	0.180 (8)	-0.033 (1)	8	1.0	5	30.0	0.05	LOO
**Lowest LogP**	N.3	0.246 (8)	0.044 (2)	0.204 (7)	8	1.0	5	30.0	0.05	LOO
**Epacadostat**	C.3	-0.040 (2)	0.248 (8)	0.159 (3)	8	1.0	5	30.0	0.05	LOO

For each alignment, a construction model was tested with the same option value for Grid spacing, Grid extension, CutOff, Min. Sigma and CV Type, changing only the probe atom to find the best result indicating in which type of alignment start building the model. The best model is highlighted in yellow

Table SI4. IDO1 3-D QSAR models optimization by varying the pretreatment parameters on the best alignment rule.

Probe Atom	q^2 STE	q^2 ELE	q^2 BOTH	ONPC	Grid Spacing	Grid Extension	CutOff	Min. Sigma	CV Type	r^2	SDEC	SDEP	r^2 YS	q^2 YS	SDEP YS
C.2	-0.041	0.262	0.158	8	1.0	5	30	0.05	LOO	/	/	/	/	/	/
C.2	-0.049	0.152	0.144	8	1.1	5	30	0.05	LOO	/	/	/	/	/	/
C.2	-0.029	0.178	0.157	8	1.2	5	30	0.05	LOO	/	/	/	/	/	/
C.2	-0.046	0.397	0.164	8	1.3	5	30	0.05	LOO	/	/	/	/	/	/
C.2	-0.060	0.129	0.123	8	1.4	5	30	0.05	LOO	/	/	/	/	/	/
C.2	-0.033	0.213	0.176	8	1.5	5	30	0.05	LOO	/	/	/	/	/	/
C.2	-0.015	0.138	0.154	8	1.6	5	30	0.05	LOO	/	/	/	/	/	/
C.2	-0.075	0.166	0.167	8	1.7	5	30	0.05	LOO	/	/	/	/	/	/
C.2	-0.092	0.181	0.119	8	1.8	5	30	0.05	LOO	/	/	/	/	/	/
C.2	-0.087	0.148	0.058	8	1.9	5	30	0.05	LOO	/	/	/	/	/	/
C.2	-0.018	0.159	0.130	8	2.0	5	30	0.05	LOO	/	/	/	/	/	/
C.2	-0.066	0.141	0.151	8	2.1	5	30	0.05	LOO	/	/	/	/	/	/
C.2	-0.031	0.149	0.167	8	2.2	5	30	0.05	LOO	/	/	/	/	/	/
C.2	-0.034	0.061	0.077	8	2.3	5	30	0.05	LOO	/	/	/	/	/	/
C.2	-0.088	0.142	0.124	8	2.4	5	30	0.05	LOO	/	/	/	/	/	/
C.2	-0.109	0.199	0.119	8	2.5	5	30	0.05	LOO	/	/	/	/	/	/
C.2	-0.136	0.157	0.041	8	2.6	5	30	0.05	LOO	/	/	/	/	/	/
C.2	-0.143	0.189	0.093	8	2.7	5	30	0.05	LOO	/	/	/	/	/	/
C.2	-0.083	0.161	0.105	8	2.8	5	30	0.05	LOO	/	/	/	/	/	/
C.2	-0.062	0.170	0.167	8	2.9	5	30	0.05	LOO	/	/	/	/	/	/
C.2	0.049	0.093	0.230	8	3.0	5	30	0.05	LOO	/	/	/	/	/	/
C.2	-0.040	0.251	0.174	8	1.29	5	30	0.05	LOO	/	/	/	/	/	/
C.2	-0.051	0.443	0.167	8	1.31	5	30	0.05	LOO	/	/	/	/	/	/
C.2	-0.072	0.362	0.153	8	1.32	5	30	0.05	LOO	/	/	/	/	/	/
C.2	-0.051	0.444	0.172	8	1.311	5	30	0.05	LOO	/	/	/	/	/	/
C.2	-0.052	0.446	0.182	8	1.312	5	30	0.05	LOO	/	/	/	/	/	/
C.2	-0.051	0.431	0.185	8	1.313	5	30	0.05	LOO	/	/	/	/	/	/
C.2	-0.093	-0.034	-0.025	8	1.312	5	3	0.05	LOO	/	/	/	/	/	/
C.2	-0.086	0.134	0.031	8	1.312	5	6	0.05	LOO	/	/	/	/	/	/
C.2	-0.081	0.314	0.105	8	1.312	5	9	0.05	LOO	/	/	/	/	/	/
C.2	-0.071	0.427	0.140	8	1.312	5	12	0.05	LOO	/	/	/	/	/	/
C.2	-0.069	0.458	0.157	8	1.312	5	14	0.05	LOO	/	/	/	/	/	/
C.2	-0.067	0.472	0.167	8	1.312	5	15	0.05	LOO	0.986	0.137	0.845	0.884	-1.453	1.768
C.2	-0.065	0.460	0.160	8	1.312	5	16	0.05	LOO	/	/	/	/	/	/
C.2	-0.063	0.447	0.157	8	1.312	5	18	0.05	LOO	/	/	/	/	/	/
C.2	-0.060	0.451	0.160	8	1.312	5	21	0.05	LOO	/	/	/	/	/	/
C.2	-0.057	0.458	0.167	8	1.312	5	24	0.05	LOO	/	/	/	/	/	/
C.2	-0.052	0.436	0.171	8	1.312	5	27	0.05	LOO	/	/	/	/	/	/
C.2	-0.050	0.439	0.177	8	1.312	5	33	0.05	LOO	/	/	/	/	/	/
C.2	-0.068	0.458	0.157	8	1.312	5	15	0.01	LOO	/	/	/	/	/	/
C.2	-0.066	0.446	0.159	8	1.312	5	15	0.04	LOO	/	/	/	/	/	/
C.2	-0.066	0.457	0.159	8	1.312	5	15	0.06	LOO	/	/	/	/	/	/
C.2	-0.068	0.470	0.164	8	1.312	5	15	0.09	LOO	/	/	/	/	/	/
C.2	-0.067	0.448	0.158	8	1.312	5	15	0.5	LOO	/	/	/	/	/	/
C.2	-0.033	0.127	0.131	8	1.312	4	15	0.05	LOO	/	/	/	/	/	/
C.2	-0.052	0.126	0.123	8	1.312	6	15	0.05	LOO	/	/	/	/	/	/

The best models (LOO and LSO) are highlighted in yellow. In red is shown the changed parameter.

Table SI5. IDO1 3-D QSAR model metrics.

**Fields**	**ONPC[a]**	r^2	**SDEC[b]**	q^2		**SDEP[c]**		r^2_{YS}	q^2_{YS}	
				**LOO[d]**	**LSO[e]**	**LOO**	**LSO**		**LOO**	**LSO**
**Steric**	2	0.541	0.771	-0.067	-0.067	1.176	1.176	0.526	-0.27	-0.28
**Electrostatic**	8	0.986	0.137	0.472	0.409	0.845	0.876	0.884	-1.45	-0.94
**Steric/Electrostatic**	8	0.988	0.123	0.156	0.192	1.047	1.024	0.940	-0.36	-0.42

**3-D QSAR Building Settings**	
**Probe Atom**	C.2
**Grid Spacing**	1.312
**Grid Extension**	5
**Minimum Sigma**	0.05
**Max/Min Energy of Cutoff Value**	15

[a] ONPC: Optimal Number of Principal Components

[b] SDEP: Cross-validated Standard Deviation Error Prediction

[c] SDEC: Standard Deviation Error Calculation

[d] LOO: Leave-One-Out

[e] LSO: Leave-Some-Out

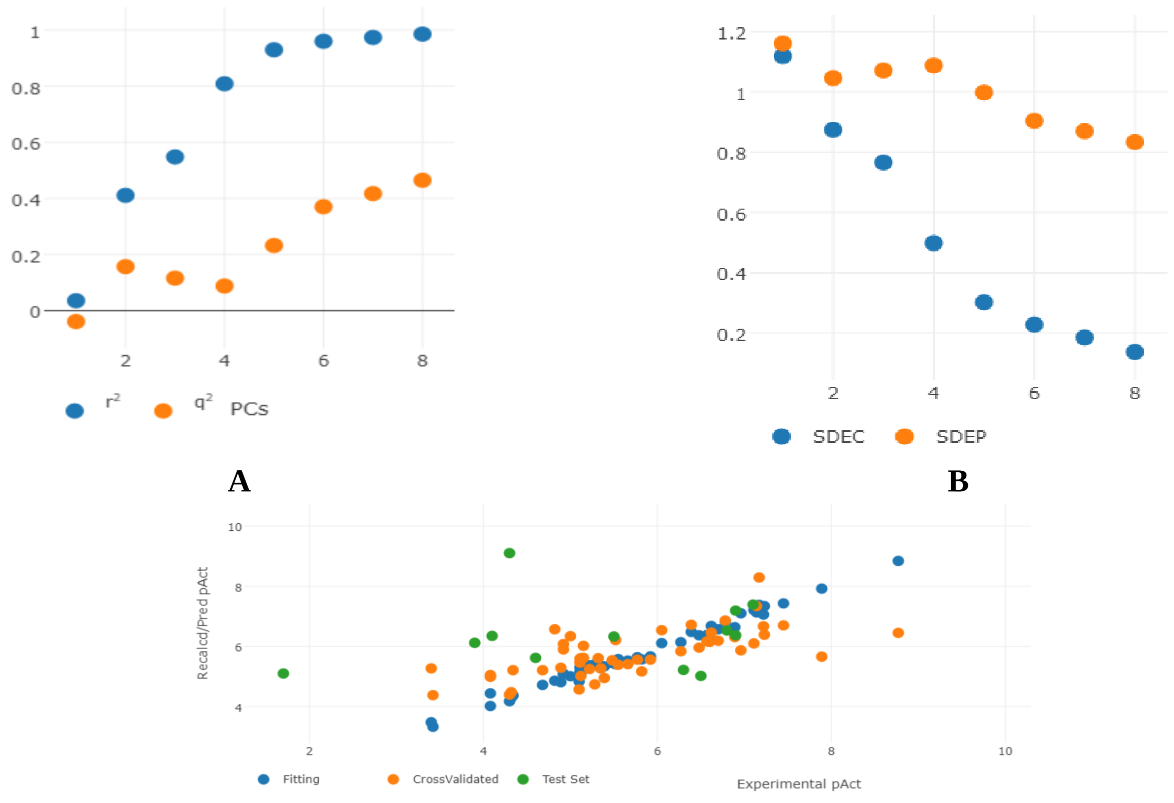

C

Figure SI3. Plots for the IDO1 dataset. **(A)** r^2 and q^2 versus the number of PCs; **(B)** SDEC and SDEP versus the number of PCs; **(C)** Recalculated and predicted versus the experimental activities.

Final model graphical inspection

3-D QSAR contour maps were generated using the Coeff x Mean to observe steric and electrostatic CoMFA like maps. In Figure SI4 the least (compound IDO1_09 in red) and most (compound IDO1_09 in blue) active molecules have been superimposed. Steric contour maps are related to increase (positive, in green) or decrease (negative, in orange) in activity, based on presence or absence of certain steric hindrance, such as Van der Waals forces (Figure SI4A). As it shown in Figure SI4A, contour maps are green where most active molecule (blue) has a steric hindrance such as benzene ring;

As for electrostatic maps, the blue colored polyhedra indicate that in this zone hydrogen bonds acceptors or generally negatively charged groups are favored, while hydrogen bonds or generally positive charge groups are unfavorable. The red polyhedral are the areas around the molecules where hydrogen bond donor groups are favored, or in general with a positive charge, while acceptor groups or generally with a negative charge could lead to lower inhibitory potency (Figure SI4B).

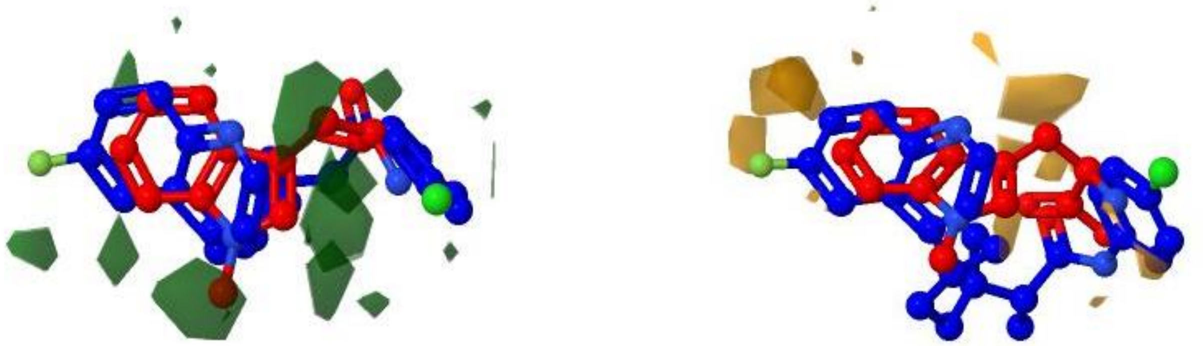

A

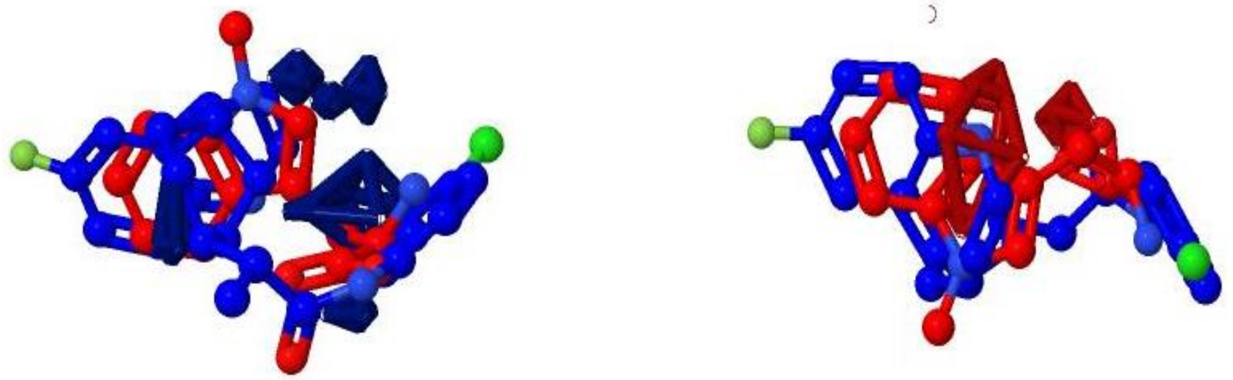

B

Figure SI4 (A) Least (in red) and most (in blue) active molecule's positive (in green) and negative (in orange) steric Coeff x Mean. **(B)** Least (in red) and most (in blue) active molecule's positive (in blue) and negative (in red) electrostatic Coeff x Mean.

Predictive Performance and Interpretation of the Final Model. As described above, during Py-CoMFA model generation, the activities of a separate test set can also be predicted. Such test set predictions for the IDO1 inhibitors are shown in Table SI6[50-51] While the overall $SDEP_{pred}$ of 1.72 was much higher than the LOO cross-validation SDEP of 1.05, it might be noted that this gap is almost entirely caused by the predicted activity for a single structure, IDO1_61, being much lower than the experimentally observed activity. In the context of an actual drug discovery project, such a promising "magic methyl" type of result might prompt the examination of IDO1_61's structure for any distinguishing features.

Table SI6. IDO1 test set experimental and predicted activity by the final 3-D QSAR model.

ID	Experimental	Predicted	Prediction Error
**IDO1_52**	4.700	5.079	-0.379
**IDO1_53**	7.100	7.349	-0.249
**IDO1_54**	6.800	6.534	0.266
**IDO1_55**	6.900	7.231	-0.331
**IDO1_56**	3.900	6.131	-2.231
**IDO1_57**	5.500	6.355	-0.855
**IDO1_58**	6.900	6.377	0.523
**IDO1_59**	4.600	5.649	-1.049
**IDO1_60**	4.100	6.366	-2.266
**IDO1_61**	4.300	9.099	-4.799

**IDO1_62**	6.300	5.186	1.114
**IDO1_63**	6.500	5.001	1.499

2. Students works [M.D.M.]: Tyrosine–Protein Phosphatase Non–Receptor Type 11 (SHP-2)

In this section is described the development of FB LB 3-D QSAR model based on the molecules described by J. Zhu et al[53] and M.J. La Marche et al[52]. The molecules were evaluated as inhibitors of SHP-2.

Overview of the biological target and connected pathology. SHP-2 (Figure SI5) is a non-receptor tyrosine phosphatase protein, presents two N-terminal tandem domains Src homology 2 called SH2, a PTP domain and a C-terminal domain [60]. Thanks to the analysis through X-ray, it was found that the conformation is composed of two N-SH2 and C-SH2 domains that bind with the PTP domain through a "tunnel" portion.[61] Aminoacid residue Glut76 plays a linking role between N-SH2 and the PTP domain, including a hydrogen bond with the residue Ser502 and a salt bridge with the residue R265.[62]

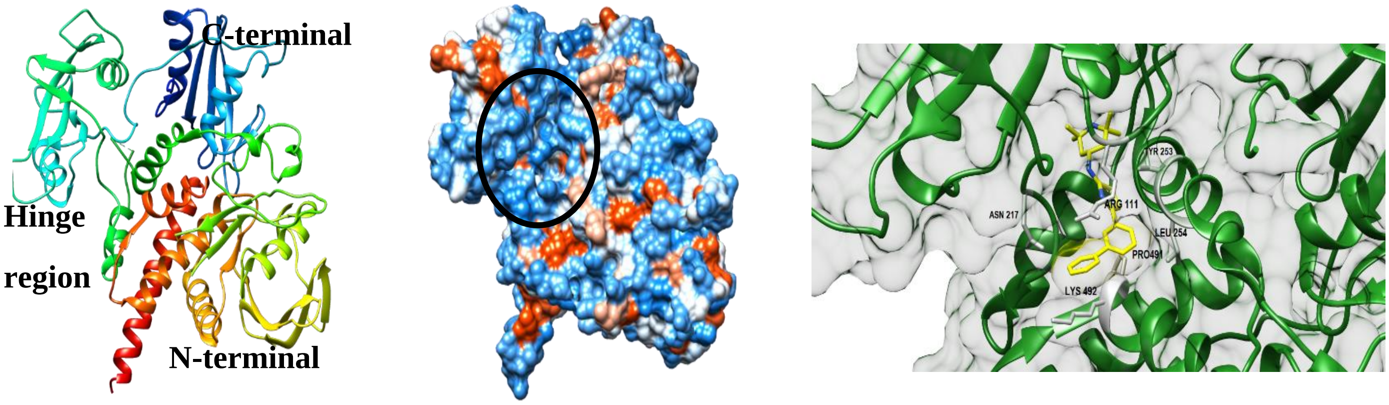

A B C

Figure SI5. Cartoon representation of SHP-2. It is composed of a C-terminal lobe (blue and light blue chains) linked by a hinge region with the N-terminal lobe (green, yellow, orange and red chains) (A). Surface representation of hydrophobic and hydrophilic sites of the protein. The black circle indicates the core (B). Cartoon, stick and surface representation of the interaction between a competitive inhibitor and binding site (C). For figure generation, the data available in the PDB entry code 5XZR was used.

SHP-2 phosphatase encoded the PTPNII gene is a non–receptor PTP containing two N-terminal Src homology 2 domains, a PTP domain and a C-terminal tail.[60] X-ray structures have demonstrated that SHP-2 adopts an self-inhibited conformation in its basal state: N-SH2 domain of SHP-2 protein binds to the PTP domain and blocks its substrate access, therefore, resulting in suppressed PTP activity. When the SH2 domains bind to specific phosphotyrosine motifs, the auto inhibitory interactions are abolished. The phosphatase is then in an open conformation that allows SHP-2 activation.[63] Protein tyrosine phosphorylation is a key modification controlling all aspects of crucial cellular processes including proliferation, differentiation, growth, and apoptosis. Dysregulation of tyrosine phosphorylation has been associated with the developmental pathologies of various human diseases such as cancer, diabetes, and autoimmune disorders.[64-65] More recently, SHP-2 was reported to bind and dephosphorylate RAS and increase its association with effector protein RAF to activate downstream proliferative RAS/ERK/MAPK signaling.[66] Also, germline or somatic mutations in PTPN11 that cause hyperactivation of SHP-2 have been identified in Noonan syndrome (50%),[67] juvenile myelomonocytic leukemia (JMML, 35%), myelodysplastic syndrome (10%), B-cell acute lymphoblastic leukemia (7%), acute myeloid leukemia (AML, 4%)[68] and solid tumors including lung adenocarcinoma, colon cancer, neuroblastoma, melanoma, and hepatocellular carcinoma.[69] SHP-2 may also participate in the programmed cell death pathway (PD-1/PD- L1) and contribute to immune evasion[70] reversing immunosuppression in the tumour microenvironment, the reason why investigating the inhibition of SHP-2 for cancer immunotherapy is also of great interest.[71] Describing the binding site, the mutated residues are located in the interface between N- SH2 and PTP domains.[72] Glutamate 76 plays a key role in bridging N-SH2 and PTP domains including a hydrogen bond with S502 hydroxyl and a salt bridge interacting with R265. Substituting glutamate 76 leads to weakened interactions between these two domains and thus arouses the destabilization of the auto inhibited conformation.[61] Mutations of E76 residue have been frequently identified in Noonan syndrome and leukaemia. Mutant E76A full-length SHP-2 (SHP-2E76A) exhibited much higher phosphatase activity than wild-type SHP-2 (SHP-2WT) in an

in vitro biochemical assay using DIFMUP as a surrogate substrate. In contrast, C459S SHP-2 (SHP-2C459S), which replaces the catalytic centre cysteine to serine, totally abolished its phosphatase activity.[62] The PTP catalytic pocket presents positive charge and it's highly conserved, that represents a unique drug discovery challenges, thus most catalytic site inhibitors require multiple ionizable functional groups in order to inhibit the enzyme.

Training set composition. The training set consisted of 40 molecules taken from two articles [52-53] (Table SI7), including two classes of compounds: the first one characterized by an aminotiazole scaffold, where adding a bromine atom increases activity; the second one characterized by pyrazine and pyrimidine scaffold, among which pyrazine showed more potency against SHP2. In Table SI8 are shown the experimental activity of each molecule and the predicted activity of the model for each molecule, with the relating error.

Table SI7. IDs, SMILES and 2-D structures of the SHP-2 datasets.[3-4]

ID	SMILES	2-D Chemical Structure
**SHP2_01**	CC1(C)CC(CC(C)(C)N1)NC1=NC(=CS1)C1=CC=CC=C1	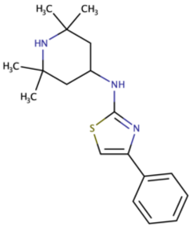
**SHP2_02**	CC1(C)CC(CC(C)(C)N1)NC1=NC(=CS1)C1=CC(=CC=C1)C1=CC=CC=C1	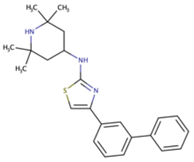
**SHP2_03**	CC1(C)CC(CC(C)(C)N1)NC1=NC(=CS1)C1=CC=C(C=C1)C1=CC=CC=C1	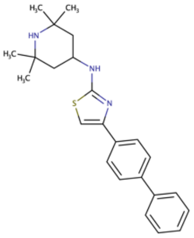
**SHP2_04**	CC1(C)CC(CC(C)(C)N1)NC1=NC(=CS1)C1=CC2=C(C=CC=C2)C=C1	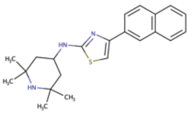
**SHP2_05**	C1CC(CCN1)NC1=NC(=CS1)C1=CC2=C(C=CC=C2)C=C1	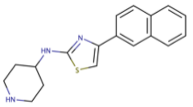
**SHP2_06**	CN1CCC(CC1)NC1=NC(=CS1)C1=CC2=C(C=CC=C2)C=C1	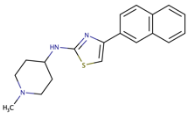
**SHP2_07**	C1CC(CN1)NC1=NC(=CS1)C1=CC2=C(C=CC=C2)C=C1	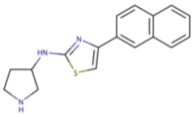
**SHP2_08**	C(NC1=NC(=CS1)C1=CC2=C(C=CC=C2)C=C1)C1CCCNC1	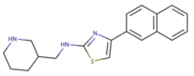
**SHP2_09**	CC1(N)CCN(CC1)C1=NC(=CS1)C1=CC2=C(C=CC=C2)C=C1	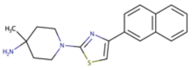
**SHP2_10**	CC1CN(CC(C)N1)C1=NC(=CS1)C1=CC2=C(C=CC=C2)C=C1	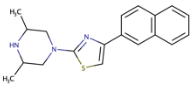
**SHP2_11**	NCC1CCCN(C1)C1=NC(=CS1)C1=CC2=C(C=CC=C2)C=C	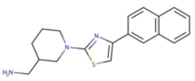
**SHP2_12**	CC1(N)CCN(CC1)C1=NC(=CS1)C1=CC2=C(C=C1)C=C(Cl)C=C2	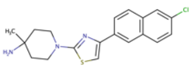

**SHP2_13**	CC1(N)CCN(CC1)C1=NC(=CS1)C1=CC2=C(C=C1)C=C(F)C=C2	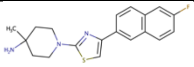
**SHP2_14**	COC1=CC2=C(C=C1)C=C(C=C2)C1=CSC(=N1)N1CCC(C)(N)CC1	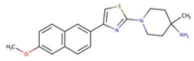
**SHP2_15**	CC1(N)CCN(CC1)C1=NC(=CS1)C1=CC2=C(C=C1)C=C(Br)C=C2	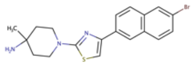
**SHP2_16**	CC1(N)CCN(CC1)C1=NC(=CS1)C1=CC2=C(C=C1)C=C(C=C2)C#N	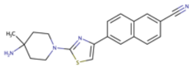
**SHP2_17**	COC(=O)C1=CC2=C(C=C1)C=C(C=C2)C1=CSC(=N1)N1CCC(C)(N)CC1	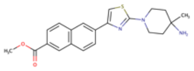
**SHP2_18**	CC1(N)CCN(CC1)C1=NC(=CS1)C1=CC2=C(C=C1)C=C(C=C2)C(O)=O	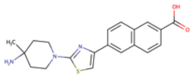
**SHP2_19**	CC1(N)CCN(CC1)C1=NC(=CS1)C1=CC(=CC=C1)C1=CC=CC=C1	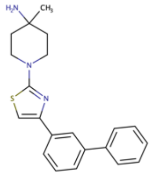
**SHP2_20**	CC1(N)CCN(CC1)C1=NC(=CS1)C1=CC(=CC=C1)C1=CC=C(C=C1)C#N	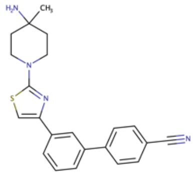
**SHP2_21**	COC(=O)C1=CC=C(C=C1)C1=CC=CC(=C1)C1=CSC(=N1)N1CCC(C)(N)CC1	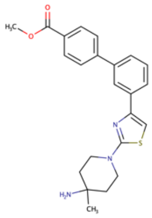
**SHP2_22**	CC1(N)CCN(CC1)C1=NC(=CS1)C1=CC=C(C=C1)C1=CC=C(F)C=C1	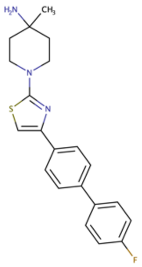
**SHP2_23**	CC1(N)CCN(CC1)C1=NC(=CS1)C1=CC=C(C=C1)C1=CC=C(O)C=C1	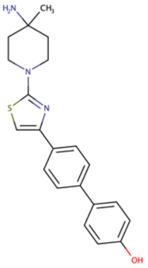
**SHP2_24**	CC1(N)CCN(CC1)C1=NC(=CS1)C1=CC2=C(C=CC=C2)N=C1	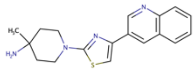
**SHP2_25**	CC1(N)CCN(CC1)C1=NC(=CS1)C1=C2OC3=C(C=CC=C3)C2=CC=C1	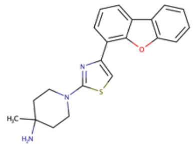
**SHP2_26**	CC1(N)CCN(CC1)C1=CN=C(C(N)=N1)C1=C(Cl)C(Cl)=CC=C1	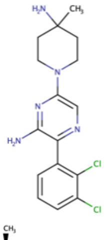
**SHP2_27**	C[C@H]1CN(C[C@@H](C)N1)C1=NC=C(C(N)=N1)C1=C(Cl)C(Cl)=CC=C1	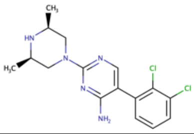

**SHP2_28**	C[C@H]1CN(C[C@@H](C)N1)C1=NC=C(C(N)=N1)C1=CC(Cl)=CC=C1	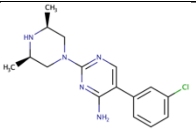
**SHP2_29**	C[C@H]1CN(C[C@@H](C)N1)C1=NC=C(C(N)=N1)C1=CC(Cl)=C(Cl)C=C1	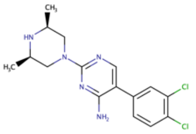
**SHP2_30**	NC1CCN(CC1)C1=NC=C(C(N)=N1)C1=C(Cl)C(Cl)=CC=C1	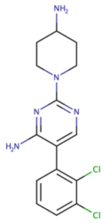
**SHP2_31**	CN(C)C1CCN(CC1)C1=NC=C(C(N)=N1)C1=C(Cl)C(Cl)=CC=C1	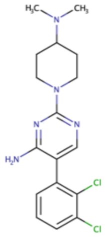
**SHP2_32**	CC1(N)CCN(CC1)C1=NC=C(C(N)=N1)C1=C(Cl)C(Cl)=CC=C1	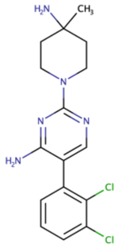
**SHP2_33**	CC1(N)CCN(CC1)C1=NN=C(C(N)=N1)C1=C(Cl)C(Cl)=CC=C1	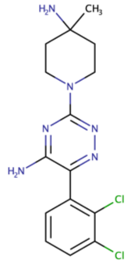
**SHP2_34**	CC1(N)CCN(CC1)C1=CN=C(C=N1)C1=C(Cl)C(Cl)=CC=C1	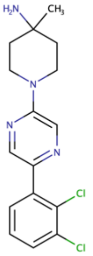
**SHP2_35**	CNC1=NC(=CN=C1C1=C(Cl)C(Cl)=CC=C1)N1CCC(C)(N)CC1	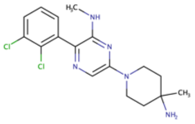
**SHP2_36**	Cn1c(c(I)c2cc(C(O)=O)c(O)cc12)-c1cccc(NC(=O)C(=O)Nc2ccc(cc2)-c2ccc(cc2)C#N)c1	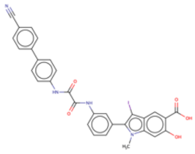
**SHP2_37**	[Na+].[Na+].Oc1c(cc(c2cccnc12)S([O-])(=O)=O)N=Nc1ccc2cc(ccc2c1)S([O-])(=O)=O	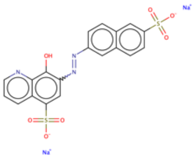
**SHP2_38**	Cn1c(c(I)c2cc(C(O)=O)c(O)cc12)-c1cccc(NC(=O)C(=O)Nc2ccc(cc2)-c2cccc(c2)C#N)c1	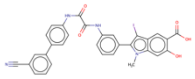

**SHP2_39**	Cn1c(c(I)c2cc(C(O)=O)c(O)cc12)-c1cccc(NC(=O)C(=O)Nc2ccc(cc2)-c2ccsc2)c1	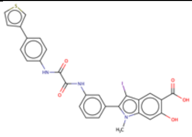
**SHP2_40**	OOOSc1ccc2cc(ccc2c1)N=Nc1cc(c2cccnc2c1O)S(O)(=O)=O	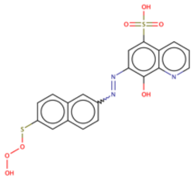
**SHP2_41***	NC(=O)C(Cc1ccc(cc1)C(F)(F)P(O)(O)=O)NC(=O)C(CC([O-])=O)NC(=O)Cc1ccc(cc1)C(F)(F)P(O)(O)=O	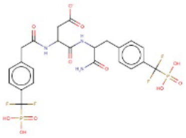
**SHP2_42***	Oc1cc2oc(c(CCc3cccc(F)c3)c2cc1C([O-])=O)-c1ccccc1	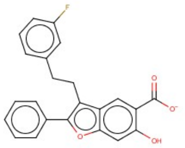
**SHP2_43***	CC1(C)C(O)CCC2(C)C1CCC1(C)C2C(=O)C=C2C3CC(C)(CCC3(C)CCC12C)C([O])=O	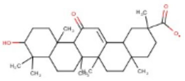
**SHP2_44***	Brc1ccc(o1)C(=O)N\N=C\c1ccc(Br)cc1	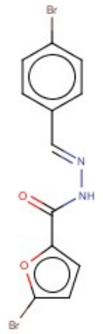
**SHP2_45***	CCOC(=O)C(Cc1ccccc1)NC(=O)N1CCc2cc(ccc12)S(=O)(=O)N1CCN(CC1)c1cccc(Cl)c1	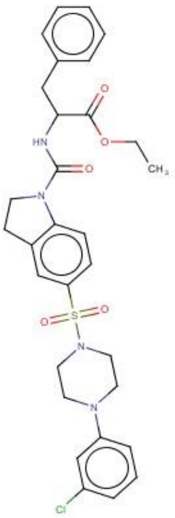
**SHP2_46***	Clc1cccc(Cl)c1NC(=S)NCCc1ccccc1	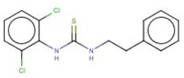
**SHP2_47***	S=C(N1CCCC1)c1cn(CCOc2ccccc2)c2ccccc12	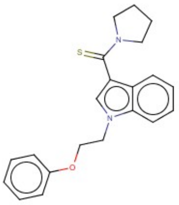

**SHP2_48***	NC(=O)C1=Cc2ccccc2O\C1=N/NC(=O)c1ccccc1O	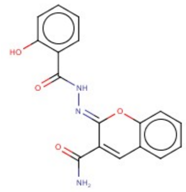
**SHP2_49***	O=C1Nc2ccc3sc4ccccc4c3c2C1=O	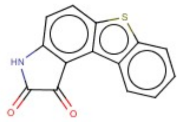
**SHP2_50***	CCOC(=O)CN1C(=O)S\C(=C\c2ccc(OCc3ccc(cc3)C([O-])=O)cc2)C1=O	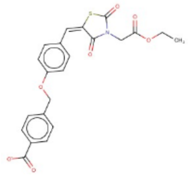

* **Test Set**

Table SI8. SHP-2 training set composition. The table shows the experimental activity of each molecule and the predicted activity of the model for each molecule, with the relative error of fitting.

ID	Experimental	Predicted (CV)	Prediction Error
**SHP-2_01**	4.720	5.076	-0.356
**SHP-2_02**	5.480	5.097	0.383
**SHP-2_03**	5.270	5.502	-0.232
**SHP-2_04**	5.590	5.617	-0.027
**SHP-2_05**	6.130	4.827	1.303
**SHP-2_06**	5.550	5.235	0.315
**SHP-2_07**	5.780	5.834	-0.054
**SHP-2_08**	5.820	5.904	-0.084
**SHP-2_09**	5.830	5.189	0.641
**SHP-2_10**	4.900	4.803	0.097
**SHP-2_11**	4.270	5.675	-1.405
**SHP-2_12**	5.570	6.036	-0.466
**SHP-2_13**	5.890	5.315	0.575
**SHP-2_14**	5.600	5.330	0.270
**SHP-2_15**	6.140	5.477	0.663
**SHP-2_16**	5.860	5.353	0.325
**SHP-2_17**	5.960	5.868	0.092
**SHP-2_18**	4.280	5.619	-1.339
**SHP-2_19**	5.440	5.853	-0.413
**SHP-2_20**	5.750	5.017	0.733
**SHP-2_21**	5.480	6.100	-0.620
**SHP-2_22**	4.840	4.955	-0.115
**SHP-2_23**	4.690	4.618	0.072
**SHP-2_24**	4.280	5.497	-1.271
**SHP-2_25**	4.330	5.157	-0.827
**SHP-2_26**	7.150	5.867	1.283
**SHP-2_27**	4.920	4.423	0.497
**SHP-2_28**	4.000	4.692	-0.692
**SHP-2_29**	4.260	4.092	0.168
**SHP-2_30**	5.880	5.905	-0.025
**SHP-2_31**	5.180	5.909	-0.729

**SHP-2_32**	6.580	5.230	1.350
**SHP-2_33**	6.520	6.076	0.444
**SHP-2_34**	5.240	5.538	-0.298
**SHP-2_35**	4.650	6.070	-1.420
**SHP-2_36**	6.650	6.762	-0.112
**SHP-2_37**	6.520	6.233	0.287
**SHP-2_38**	6.500	6.629	-0.129
**SHP-2_39**	6.690	5.730	0.960
**SHP-2_40**	6.450	6.240	0.210

3-D QSAR model building.

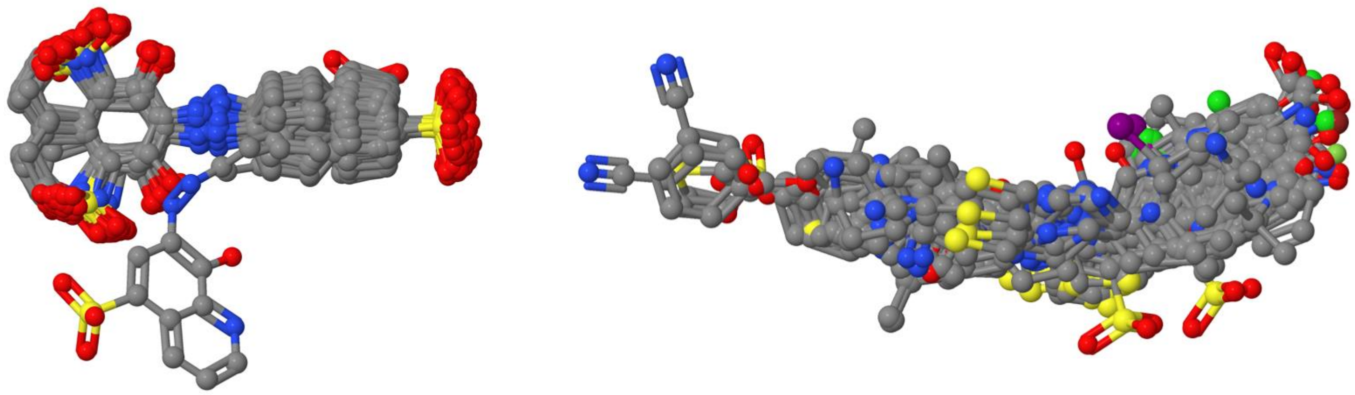

A **B**

Figure SI6. SHP-2 alignment rules. **(A)** Example of conformations obtained for the compound SHP2-29. **(B)** Training set aligned on longest alignment rule.

The best model obtained was optimized in terms of probes atom, grid spacing, grid extension and so on to find the best 3-D QSAR model optimized (Table SI9). The best model was obtained with C.3 probe; it had an electrostatic q^2 of -0.049, a r^2 of 0.847, SDEC of 0.312 and SDEP of 0.275 considering 4 ONPCs. The best result setting were obtained with grid spacing 2.6 Å, minimum sigma 0.10, grid extension 5 Å, and cutoff energy of 20 kcal/mol. LSO cross-validation was also performed in addition to LOO, in order to better evaluate the model stability. Finally, a Y-scrambling (Y_s) was carried out to verify any lack of fortuitous correlation. The model was not affected by chance correlations as the q^2 results resulted al negative. (Table SI10-11)

Table SI9. SHP-2 3-D QSAR coefficients obtained for the different alignment rules.

Alignment Rules	Probe Atom	q^2 STE	q^2 ELE	q^2 BOTH	Max ONPC	Grid Spacing	Grid Extension	CutOff	Min. Sigma	CV Type
**Least Active**	C.3	-0.214(1)	0.194(4)	-0.030(4)	8	1.0	5	30.0	0.05	LOO
**Most Active**	C.3	-0.186(1)	0.144(5)	0.047(4)	8	1.0	5	30.0	0.05	LOO
**Heaviest**	N.3	-0.319(2)	-0.044(1)	0.028(6)	8	1.0	5	30.0	0.05	LOO
**Longest**	C.3	-0.009(2)	-0.049(1)	-0.032(1)	8	1.0	5	30.0	0.05	LOO
**Most Flexible**	H	0.064(2)	-0.054(1)	-0.044(1)	8	1.0	5	30.0	0.05	LOO
**Most Rigid**	H	0.140(6)	-0.013(4)	0.058(6)	8	1.0	5	30.0	0.05	LOO
**Least Polar**	C.cat	-0.032(4)	-0.045(1)	-0.036(1)	8	1.0	5	30.0	0.05	LOO
**Most Polar**	C.2	-0.041(2)	0.262(8)	0.158(3)	8	1.0	5	30.0	0.05	LOO
**Highest MR**	C.3	-0.326(2)	-0.044(1)	0.027(6)	8	1.0	5	30.0	0.05	LOO
**Lowest MR**	C.cat	-0.120(8)	-0.042(1)	-0.035(1)	8	1.0	5	30.0	0.05	LOO
**Highest HA**	C.2	-0.326(2)	-0.044(1)	0.019(6)	8	1.0	5	30.0	0.05	LOO
**Lowest HA**	C.2	0.075(6)	-0.050(1)	-0.003(8)	8	1.0	5	30.0	0.05	LOO
**Highest HD**	O.3	-0.041(2)	0.247(8)	0.163(3)	8	1.0	5	30.0	0.05	LOO
**Lowest HD**	N.3	-0.068(4)	0.125(2)	0.039(2)	8	1.0	5	30.0	0.05	LOO
**Highest LogP**	C.2	-0.138(2)	0.180(8)	-0.033(1)	8	1.0	5	30.0	0.05	LOO
**Lowest LogP**	N.3	0.246(8)	0.044(2)	0.204(7)	8	1.0	5	30.0	0.05	LOO

For each alignment, a construction model was tested with the same option value for Grid spacing, Grid extension, CutOff, Min. Sigma and CV Type, changing only the probe atom to find the best result indicating in which type of alignment start building the model. The best model is highlighted in yellow.

Table SI10. SHP-2 3-D QSAR models optimization by varying the pretreatment parameters on the best alignment rule.

Probe Atom	q^2 STE	q^2 ELE	q^2 BOTH	ONPC	Grid Spacing	Grid Extension	CutOff	Min. Sigma	CV Type	r^2	SDEC	SDEP	r^2 YS	q^2 YS	SDEP YS
H	0.310	0.118	0.123	8	1.0	5	30.0	0.05	LOO	/	/	/	/	/	/
H	0.260	0.119	0.123	8	1.1	5	30.0	0.05	LOO	/	/	/	/	/	/
H	0.346	0.119	0.135	8	1.2	5	30.0	0.05	LOO	/	/	/	/	/	/
H	0.285	0.118	0.123	8	1.3	5	30.0	0.05	LOO	/	/	/	/	/	/
H	0.371	0.119	0.178	8	1.4	5	30.0	0.05	LOO	/	/	/	/	/	/
H	0.241	0.119	0.124	8	1.5	5	30.0	0.05	LOO	/	/	/	/	/	/
H	0.224	0.120	0.124	8	1.6	5	30.0	0.05	LOO	/	/	/	/	/	/
H	0.248	0.119	0.122	8	1.7	5	30.0	0.05	LOO	/	/	/	/	/	/
H	0.273	0.118	0.122	8	1.8	5	30.0	0.05	LOO	/	/	/	/	/	/
H	0.287	0.119	0.125	8	1.9	5	30.0	0.05	LOO	/	/	/	/	/	/
H	0.371	0.119	0.120	8	2.0	5	30.0	0.05	LOO	/	/	/	/	/	/
H	0.155	0.118	0.121	8	2.1	5	30.0	0.05	LOO	/	/	/	/	/	/
H	0.125	0.119	0.123	8	2.2	5	30.0	0.05	LOO	/	/	/	/	/	/
H	0.181	0.120	0.124	8	2.3	5	30.0	0.05	LOO	/	/	/	/	/	/
H	0.107	0.120	0.123	8	2.4	5	30.0	0.05	LOO	/	/	/	/	/	/
H	0.164	0.120	0.124	8	2.5	5	30.0	0.05	LOO	/	/	/	/	/	/
H	0.406	0.119	0.228	8	2.6	5	30.0	0.05	LOO	/	/	/	/	/	/
H	0.225	0.120	0.125	8	2.7	5	30.0	0.05	LOO	/	/	/	/	/	/
H	0.378	0.120	0.205	8	2.8	5	30.0	0.05	LOO	/	/	/	/	/	/
H	0.119	0.120	0.122	8	2.9	5	30.0	0.05	LOO	/	/	/	/	/	/
H	0.097	0.122	0.125	8	3.0	5	30.0	0.05	LOO	/	/	/	/	/	/
H	0.406	0.119	0.228	8	2.6	5	30.0	0.05	LOO	/	/	/	/	/	/
H	0.412	0.120	0.275	8	2.6	5	20.0	0.05	LOO	/	/	/	/	/	/
H	0.417	0.119	0.251	8	2.6	5	25.0	0.05	LOO	/	/	/	/	/	/
H	0.384	0.119	0.202	8	2.6	5	35.0	0.05	LOO	/	/	/	/	/	/
H	0.364	0.119	0.175	8	2.6	5	40.0	0.05	LOO	/	/	/	/	/	/
H	0.412	0.120	0.275	8	2.6	5	20.0	0.05	LOO	/	/	/	/	/	/
H	0.252	0.119	0.125	8	2.6	6	20.0	0.05	LOO	/	/	/	/	/	/
H	0.110	0.121	0.124	8	2.6	7	20.0	0.05	LOO	/	/	/	/	/	/
H	0.099	0.122	0.124	8	2.6	8	20.0	0.05	LOO	/	/	/	/	/	/
H	0.215	0.122	0.129	8	2.6	9	20.0	0.05	LOO	/	/	/	/	/	/
H	0.357	0.121	0.144	8	2.6	10	20.0	0.05	LOO	/	/	/	/	/	/
H	0.412	0.120	0.275	8	2.6	5	20.0	0.05	LOO	/	/	/	/	/	/
H	0.412	0.120	0.275	8	2.6	5	20.0	0.10	LOO	/	/	/	/	/	/
H	0.412	0.120	0.275	8	2.6	5	20.0	0.15	LOO	/	/	/	/	/	/
H	0.412	0.120	0.275	8	2.6	5	20.0	0.20	LOO	/	/	/	/	/	/
H	0.412	0.120	0.275	8	2.6	5	20.0	0.25	LOO	/	/	/	/	/	/
H	0.412	0.120	0.275	8	2.6	5	20.0	0.30	LOO	/	/	/	/	/	/
H	0.412	0.120	0.275	8	2.6	5	20.0	0.35	LOO	/	/	/	/	/	/
H	0.412	0.120	0.275	8	2.6	5	20.0	0.40	LOO	/	/	/	/	/	/
H	0.412	0.120	0.275	8	2.6	5	20.0	0.45	LOO	/	/	/	/	/	/
H	0.412	0.120	0.275	8	2.6	5	20.0	0.50	LOO	/	/	/	/	/	/
H	0.412	0.120	0.275	8	2.6	5	20.0	0.55	LOO	/	/	/	/	/	/
H	0.412	0.120	0.275	8	2.6	5	20.0	0.60	LOO	/	/	/	/	/	/
H	0.412	0.120	0.275	8	2.6	5	20.0	0.65	LOO	/	/	/	/	/	/
H	0.412	0.120	0.275	8	2.6	5	20.0	0.70	LOO	/	/	/	/	/	/
H	0.412	0.120	0.275	8	2.6	5	20.0	0.75	LOO	/	/	/	/	/	/
H	0.412	0.120	0.275	8	2.6	5	20.0	0.80	LOO	/	/	/	/	/	/
H	0.412	0.120	0.275	8	2.6	5	20.0	0.85	LOO	/	/	/	/	/	/
H	0.412	0.120	0.275	8	2.6	5	20.0	0.90	LOO	/	/	/	/	/	/
H	0.412	0.120	0.275	8	2.6	5	20.0	0.95	LOO	/	/	/	/	/	/
H	0.412	0.120	0.275	8	2.6	5	20.0	1.00	LOO	/	/	/	/	/	/
H	0.413	0.120	0.275	8	2.6	5	20.0	1.05	LOO	/	/	/	/	/	/
H	0.413	0.120	0.275	8	2.6	5	20.0	1.10	LOO	/	/	/	/	/	/
H	0.413	0.120	0.275	8	2.6	5	20.0	1.15	LOO	/	/	/	/	/	/
H	0.413	0.120	0.275	8	2.6	5	20.0	1.20	LOO	/	/	/	/	/	/
H	0.413	0.120	0.275	8	2.6	5	20.0	1.25	LOO	/	/	/	/	/	/
H	0.413	0.120	0.275	8	2.6	5	20.0	1.30	LOO	/	/	/	/	/	/
H	0.413	0.120	0.275	8	2.6	5	20.0	1.35	LOO	/	/	/	/	/	/
H	0.413	0.120	0.275	8	2.6	5	20.0	1.40	LOO	/	/	/	/	/	/
H	0.413	0.120	0.275	8	2.6	5	20.0	1.45	LOO	/	/	/	/	/	/
H	0.413	0.120	0.275	8	2.6	5	20.0	1.50	LOO	/	/	/	/	/	/
H	0.413	0.120	0.275	8	2.6	5	20.0	1.55	LOO	/	/	/	/	/	/
H	0.413	0.120	0.275	8	2.6	5	20.0	1.60	LOO	/	/	/	/	/	/
H	0.413	0.120	0.275	8	2.6	5	20.0	1.65	LOO	/	/	/	/	/	/
H	0.413	0.120	0.276	8	2.6	5	20.0	1.70	LOO	/	/	/	/	/	/
H	0.413	0.121	0.276	8	2.6	5	20.0	1.75	LOO	/	/	/	/	/	/
H	0.413	0.121	0.276	8	2.6	5	20.0	1.80	LOO	/	/	/	/	/	/
H	0.412	0.121	0.276	8	2.6	5	20.0	1.85	LOO	/	/	/	/	/	/
H	0.412	0.121	0.277	8	2.6	5	20.0	1.90	LOO	/	/	/	/	/	/
H	0.412	0.121	0.277	8	2.6	5	20.0	1.95	LOO	/	/	/	/	/	/
H	0.412	0.121	0.278	8	2.6	5	20.0	2.00	LOO	/	/	/	/	/	/
H	0.310	0.118	0.123	8	1.0	5	30.0	0.05	LOO	/	/	/	/	/	/
H	0.260	0.119	0.123	8	1.1	5	30.0	0.05	LOO	/	/	/	/	/	/
H	0.346	0.119	0.135	8	1.2	5	30.0	0.05	LOO	/	/	/	/	/	/
H	0.285	0.118	0.123	8	1.3	5	30.0	0.05	LOO	/	/	/	/	/	/
H	0.371	0.119	0.178	8	1.4	5	30.0	0.05	LOO	/	/	/	/	/	/
H	0.241	0.119	0.124	8	1.5	5	30.0	0.05	LOO	/	/	/	/	/	/
H	0.224	0.120	0.124	8	1.6	5	30.0	0.05	LOO	/	/	/	/	/	/
H	0.248	0.119	0.122	8	1.7	5	30.0	0.05	LOO	/	/	/	/	/	/

The best models (LOO and LSO) are highlighted in yellow. In red is shown the changed parameter.

Table SI11. SHP-2 3-D QSAR model metrics

Fields	ONPC[a]	r^2	SDEC[b]	q^2		SDEP[c]		r^2_{YS}	q^2_{YS}	
				LOO[d]	LSO[e]	LOO	LSO		LOO	LSO
**Steric**	4	0.847	0.312	0.412	0.410	0.611	0.612	0.761	-0.670	-0.462
**Electrostatic**	1	0.187	0.719	0.120	0.128	0.748	0.744	0.055	-0.124	-0.106
**Steric/Electrostatic**	4	0.788	0.367	0.275	0.298	0.679	0.668	0.613	-0.613	-0.513

**3-D QSAR Building Settings**	
**Probe Atom**	H
**Grid Spacing**	2.2
**Grid Extension**	10
**Minimum Sigma**	1.5
**Max/Min Energy of Cutoff Value**	25

[a] ONPC: Optimal Number of Principal Components
[b] SDEP: Cross-validated Standard Deviation Error Prediction
[c] SDEC: Standard Deviation Error Calculation
[d] LOO: Leave-One-Out
[e] LSO: Leave-Some-Out

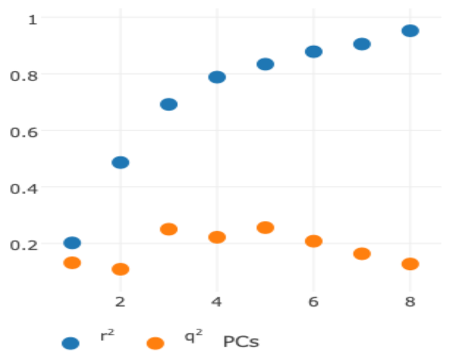

A

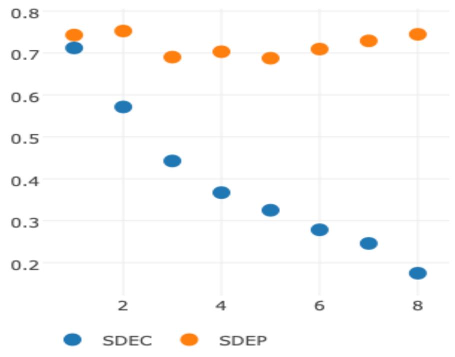

B

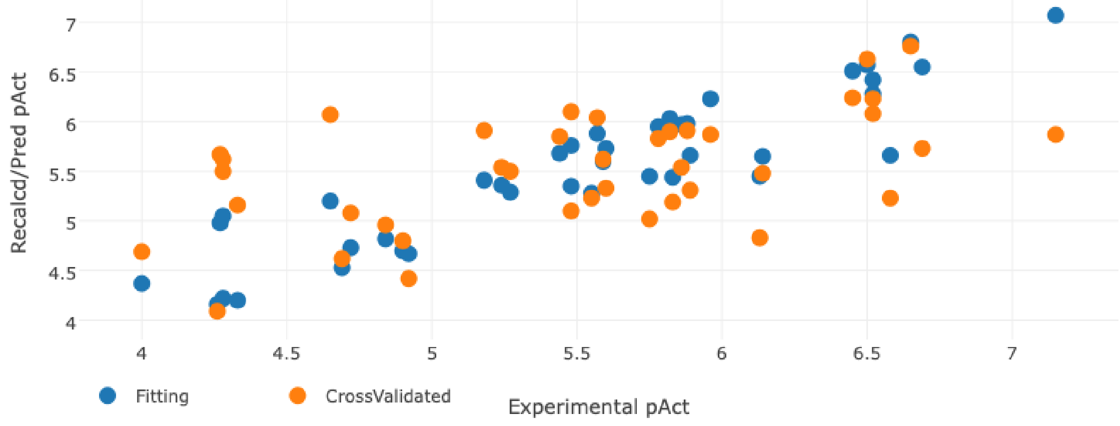

C

Figure SI7. Plots for the SHP2 dataset. **(A)** r^2 and q^2 versus the number of PCs; **(B)** SDEC and SDEP versus the number of PCs; **(C)** Recalculated and predicted versus the experimental activities.

Final model graphical inspection.

By best model 3-D QSAR contour maps are generated using the Coeff x Mean, to observe steric and electrostatic CoMFA like maps. First of all, in Figure SI8 the least (compound SHP-2_28 in red) and most (compound SHP-2_26 in blue) active molecules have been overlying. Given the presence of a fluorine atom (in light green) on most active molecule's terminal portion, can be hypothesized that presence of a large atom can be important for the activity. Steric contour maps are related to increase (positive, in green) or decrease (negative, in orange) in activity, based on presence or absence of certain steric hindrance, such as Van der Waals forces (Figure SI8A).

Electrostatic contour maps give information on molecules' charge to observe whether attractive or repulsive forces are favored: in positive electrostatic contour maps (green polyhedral) attractive forces are favored, instead in negative electrostatic contour maps (orange polyhedral) repulsive forces are favored (Figure SI8B).

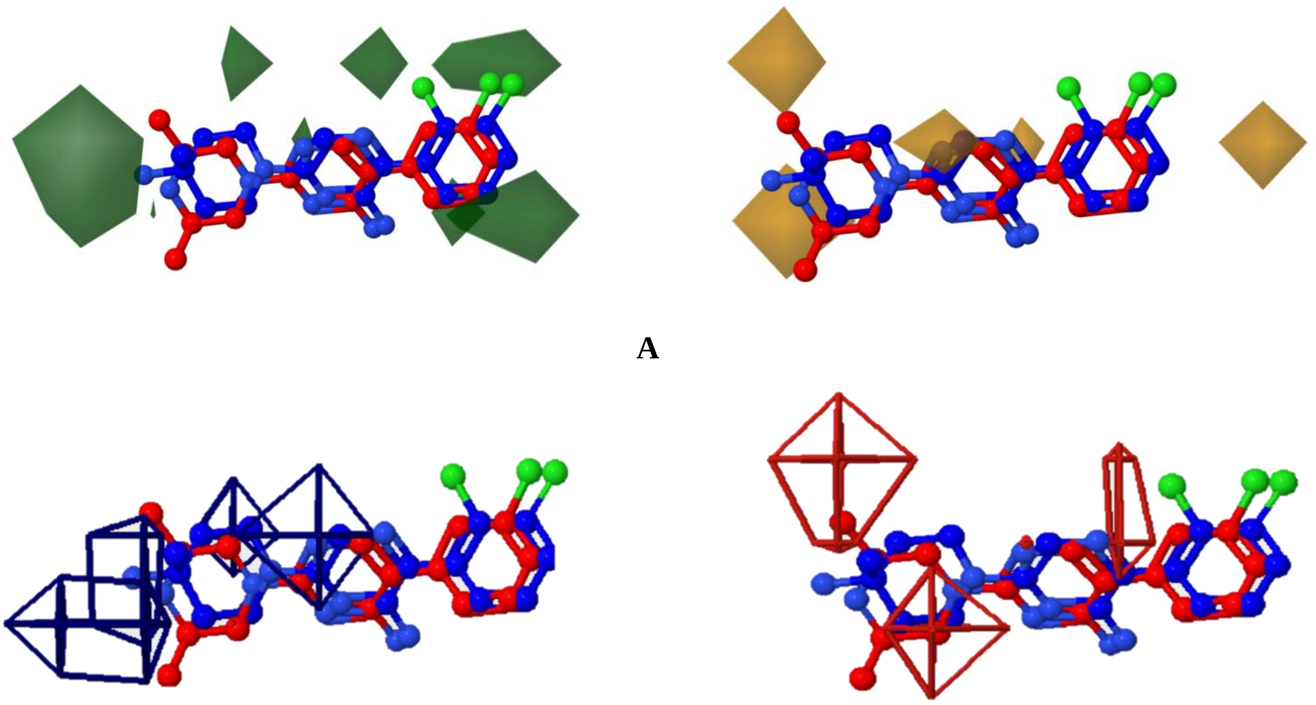

A

B

Figure SI8 (A) Least (in red) and most (in blue) active molecule's positive (in green) and negative (in yellow) steric Coeff x Mean. (B) Least (in red) and most (in blue) active molecule's positive (in blue) and negative (in red) electrostatic Coeff x Mean.

Test set composition and predictive ability evaluation. Regarding the external validation about the test set, the following table summarized the difference between experimental and predicted activities. The prediction errors are low, except for the molecules SHP-2_41 and SHP-2_43 (Table SI12).

Table SI12. SHP-2 test set experimental and predicted activity by the final 3-D QSAR model.

ID	Experimental	Predicted	Error
**SHP-2_41**	4.900	6.173	-1.270
**SHP-2_42**	5.000	4.450	0.580
**SHP-2_43**	5.010	5.934	-0.920
**SHP-2_44**	5.040	4.620	0.420

**SHP-2_45**	5.160	5.289	-0.130
**SHP-2_46**	5.230	5.110	0.120
**SHP-2_47**	5.260	5.100	0.160
**SHP-2_48**	5.270	5.609	-0.340
**SHP-2_49**	5.490	5.210	0.230
**SHP-2_50**	5.490	5.340	0.150

3. Students works [S.M.]: Interleukin-1 Receptor Associated Kinase-4 (IRAK4)

In this section is described the development of FB LB 3-D QSAR model based on the molecules described Scott J.S. et al[54] and Lee K.L. et al.[54-55] The molecules were evaluated as an inhibitor of IRAK4.

Overview of the biological target and connected pathology. IRAK4 (Figure SI9) is a serine-threonine kinase that is known to have biologically important kinase activity.[73] IRAK4 and all members of the family (including IRAK1, IRAK2 and IRAKM) facilitate key protein scaffolding and regulatory functions in various signaling pathways[74-75]. IRAK4, in particular, participates as a key mediator in the *Interleukin-1/Toll-like Receptor (IL-1/TLR)* signaling cascades.[76-77] This kinase made up of 460 amino acids, which contains both a kinase domain and a death domain.[76] Its kinase domain exhibits the typical bilobal structure of kinases, with the N–terminal lobe consisting of five-stranded antiparallel β–sheet and one α–helix, while the C–terminal lobe is composed mainly of a few α–helices. Situated where the two lobes meet is an ATP binding site, which is covered by a tyrosine gatekeeper. Tyrosine as a gatekeeper is believed to be unique to the IRAK family of kinases[78]. The protein also contains three auto-phosphorylation sites, each of which when mutated results in a decrease in the kinase activity of IRAK4[79]. Signaling through *IL-1R/TLR* results in the activation of adaptor protein myeloid differentiation primary response *gene 88 (MyD88)* which recruits IRAK4 and IRAK1 to form a signaling complex. This complex then interacts with a series of kinases, adaptor proteins and ligases eventually resulting in the activation of the Transcription Factors Kappa light chain enhancer of activated B cells (NF-κB) and Activator Protein-1 (AP-1), ultimately inducing the generation of inflammatory cytokines[80-81]. IRAK4 is proposed to play a role in lymphomas that contain a mutation in the MYD88 adaptor protein: *L265P*[82]. This mutation regards *diffuse large B–cell lymphoma (DLBCL)*, that is the most common *B–cell non–Hodgkin lymphoma (NHL)* comprising 30–35% of all NHLs[83]. IRAK4 inhibition, together with inhibition of B-cell receptor (BCR) signaling could have synergistic effects on ABC DLBCL killing[84].

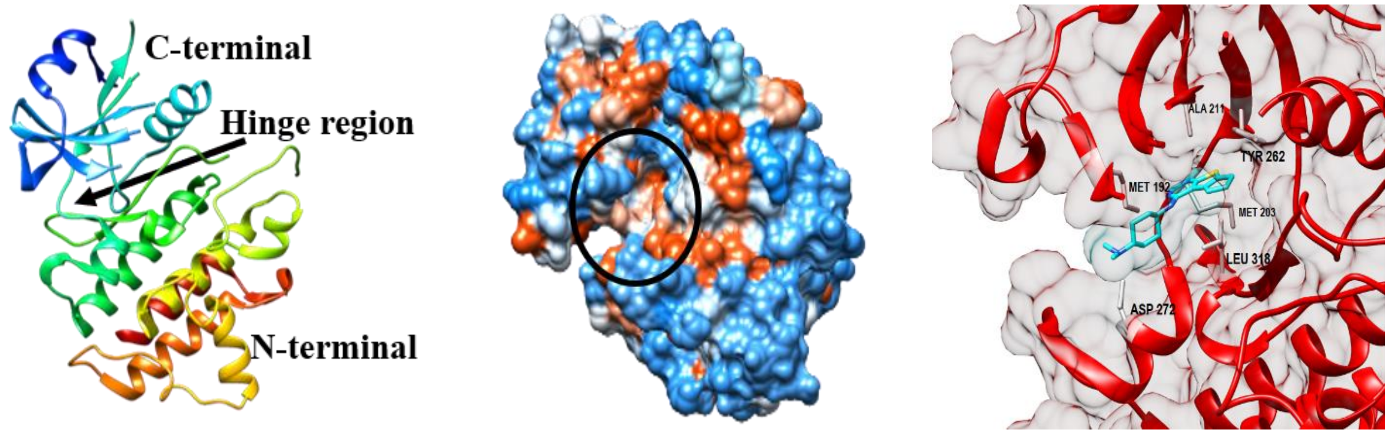

A B C

Figure SI9. Cartoon representation of IRAK4 three-dimensional structure **(A)**: It is composed of a C-terminal lobe (blue and light blue chains) linked by a hinge region with the N-terminal lobe (green, yellow, orange and red chains). **(B)**: Surface representation of hydrophobic and hydrophilic sites of the protein; the black circle indicates the core. **(C)**: Cartoon, stick and surface representation of the interaction between a competitive inhibitor and ATP binding site. For figure generation the PDB entry code 5k75 was used.

Training set composition. The training set consists of 58 molecules arising from two articles[54-55] (Table SI13). Most of these compounds were characterized by a tricyclic scaffold that showed potency against IRAK4 and a good level of selectivity against a panel of kinases.[85] In Table SI14 are shown the experimental activity of each molecule and the predicted activity of the model for each molecule, with the relating error.

Table SI13. IDs, SMILES and 2-D structures of the IRAK4 datasets.[5-6]

ID	SMILES	2-D Chemical Structure
**IRAK4_01**	CN([C@H]1CC[C@@H](CC1)Nc2ncnc3c2c4c(s3)CCC4)C	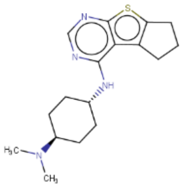
**IRAK4_02**	Cc1c2c(sc1C)ncnc2N[C@H]3CC[C@@H](CC3)N(C)C	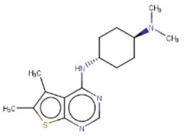
**IRAK4_03**	Cc1sc2ncnc(c2c1)N[C@H]3CC[C@@H](CC3)N(C)C	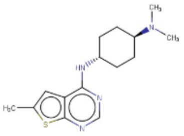
**IRAK4_04**	Cc1c2c(sc1)ncnc2N[C@H]3CC[C@@H](CC3)N(C)C	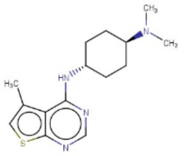
**IRAK4_05**	CN([C@H]1CC[C@@H](CC1)Nc2ncnc3c2ccs3)C	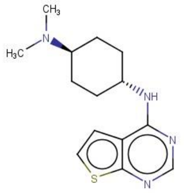
**IRAK4_06**	CN([C@H]1CC[C@@H](CC1)Nc2ncnc3c2c4c([nH]3)CCC4)C	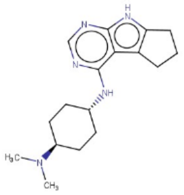
**IRAK4_07**	Cc1c2c(ncnc2[nH]c1C)N[C@H]3CC[C@@H](CC3)N(C)C	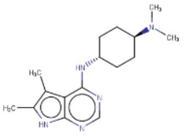
**IRAK4_08**	Cc1c2c(ncnc2[nH]c1)N[C@H]3CC[C@@H](CC3)N(C)C	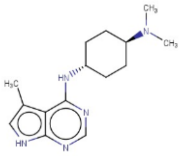
**IRAK4_09**	CN([C@H]1CC[C@@H](CC1)Nc2ncnc3c2cc[nH]3)C	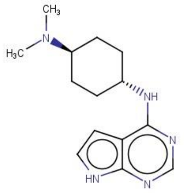
**IRAK4_10**	CCc1c2c(ncnc2[nH]c1)N[C@H]3CC[C@@H](CC3)N(C)C	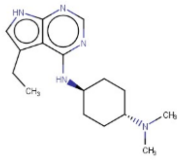
**IRAK4_11**	CC(c1c2c(ncnc2[nH]c1)N[C@H]3CC[C@@H](CC3)N(C)C)C	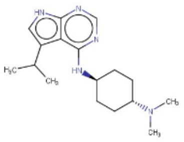

**IRAK4_12**	CC(c1c2c(ncnc2[nH]c1)N[C@H]3CC[C@@H](CC3)N(C)C)(C)C	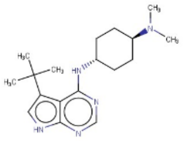
**IRAK4_13**	CN([C@H]1CC[C@@H](CC1)Nc2ncnc3c2c(c[nH]3)C(F)(F)F)C	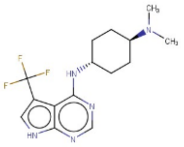
**IRAK4_14**	CN([C@H]1CC[C@@H](CC1)Nc2ncnc3c2c(c[nH]3)C4CC4)C	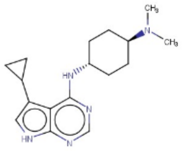
**IRAK4_15**	CN([C@H]1CC[C@@H](CC1)Nc2ncnc3c2c(c[nH]3)C4CCC4)C	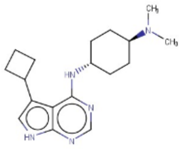
**IRAK4_16**	CN([C@H]1CC[C@@H](CC1)Nc2ncnc3c2c(c[nH]3)C4CCCC4)C	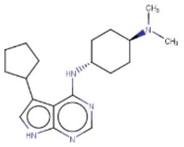
**IRAK4_17**	CN([C@H]1CC[C@@H](CC1)Nc2ncnc3c2c(c[nH]3)C4OCCC4)C	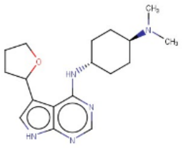
**IRAK4_18**	CN([C@H]1CC[C@@H](CC1)Nc2ncnc3c2c(c[nH]3)C4COCC4)C	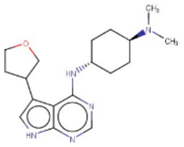
**IRAK4_19**	CN([C@H]1CC[C@@H](CC1)Nc2ncnc3c2c(c[nH]3)C4COCCC4)C	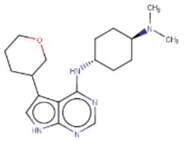
**IRAK4_20**	CN([C@H]1CC[C@@H](CC1)Nc2ncnc3c2c(c[nH]3)C4CCOCC4)C	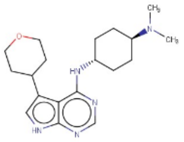
**IRAK4_21**	CN([C@H]1CC[C@@H](CC1)N(c2ncnc3c2c(c[nH]3)C4CCOCC4)C)C	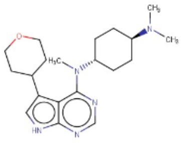
**IRAK4_22**	CN([C@H]1CC[C@@H](CC1)Oc2ncnc3c2c(c[nH]3)C4CCOCC4)C	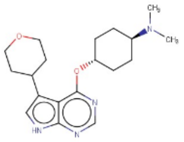
**IRAK4_23**	CC(=O)N1CCN(CC1)[C@H]2CC[C@@H](CC2)Nc3ncnc4c3c(c[nH]4)C5CCOCC5	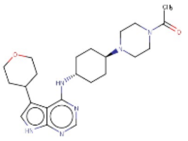
**IRAK4_24**	CN(C(=O)N1CCN(CC1)[C@H]2CC[C@@H](CC2)Nc3ncnc4c3c(c[nH]4)C5CCOCC5)C	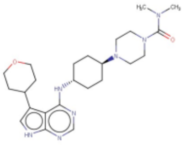

**IRAK4_25**	CS(=O)(=O)N1CCN(CC1)[C@H]2CC[C@@H](CC2)Nc3ncnc4c3c(c[nH]4)C5CCOCC5	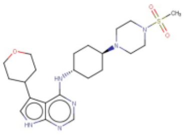
**IRAK4_26**	COC(=O)N1CCN(CC1)[C@H]2CC[C@@H](CC2)Nc3ncnc4c3c(c[nH]4)C5CCOCC5	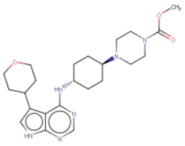
**IRAK4_27**	c1[nH]c2ncnc(c2c1C3CCOCC3)N[C@H]4CC[C@@H](CC4)N5CCOCC5	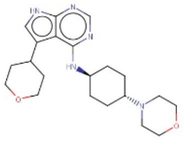
**IRAK4_28**	CN(C(=O)C1CCN(CC1)[C@H]2CC[C@@H](CC2)Nc3ncnc4c3c(c[nH]4)C5CCOCC5)C	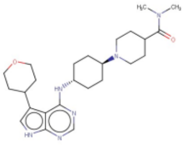
**IRAK4_29**	CN1C(=O)C[C@@H]2[C@H]1CCN(C2)[C@H]3CC[C@@H](CC3)Nc4ncnc5c4c(c[nH]5)C6CCOCC6	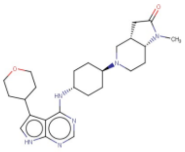
**IRAK4_30**	c1[nH]c2ncnc(c2c1C3CCOCC3)N[C@H]4CC[C@H](CC4)N5CCOCC5	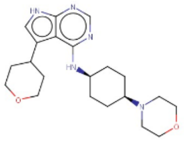
**IRAK4_31**	c1[nH]c2ncnc(c2c1C3CCOCC3)O[C@H]4CC[C@@H](CC4)N5CCOCC5	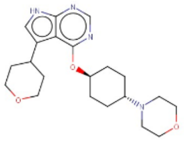
**IRAK4_32**	c1[nH]c2ncnc(c2c1C3CCOCC3)S[C@H]4CC[C@@H](CC4)N5CCOCC5	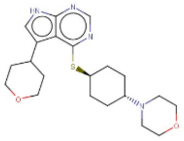
**IRAK4_33**	c1c(c2c(nc1)[nH]cc2C3CCOCC3)N[C@H]4CC[C@@H](CC4)N5CCOCC5	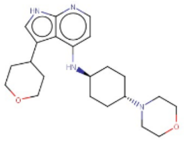
**IRAK4_34**	c1[nH]c2ncc(c(c2c1C3CCOCC3)N[C@H]4CC[C@@H](CC4)N5CCOCC5)C#N	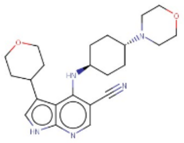
**IRAK4_35**	CC(C)Oc1cc2ccccc2cc1C(=O)N	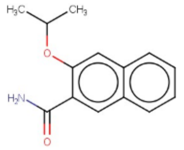
**IRAK4_36**	CC(C)Oc1cc2c(cccc2c3ccc(cc3)C#N)cc1C(=O)N	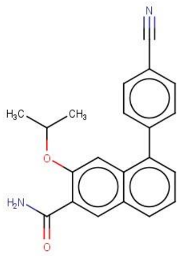

**IRAK4_37**	CC(C)Oc1cc2c(ccnc2cc1C(=O)N)O[C@@H]3CCCNC3	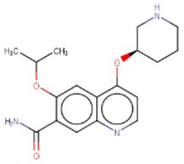
**IRAK4_38**	CC(C)Oc1cc2c(ccnc2O[C@@H]3CCCNC3)cc1C(=O)N	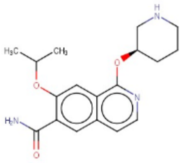
**IRAK4_39**	CC(C)Oc1cc2c(OC[C@@H]3CNC(=O)C3)nccc2cc1C(N)=O	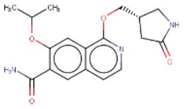
**IRAK4_40**	COc1cc2c(OC[C@H]3CCC(=O)N3)nccc2cc1C(N)=O	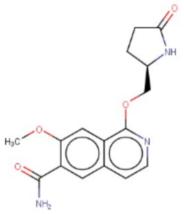
**IRAK4_41**	CCOc1cc2c(OC[C@H]3CCC(=O)N3)nccc2cc1C(N)=O	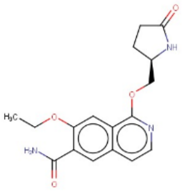
**IRAK4_42**	COc1cc2c(OC[C@H]3CCC(=O)N3)ccnc2cc1C(N)=O	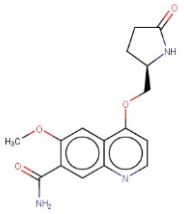
**IRAK4_43**	CC(C)Oc1cc2c(OC[C@H]3CCC(=O)N3)nccc2cc1C(N)=O	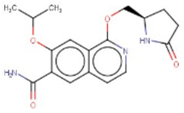
**IRAK4_44**	COc1cc2c(OC[C@H]3CCC(=O)N3)cccc2cc1C(N)=O	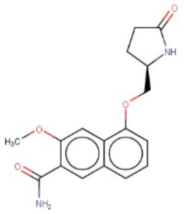
**IRAK4_45**	COc1cc2c(OC[C@H]3NC(=O)C[C@H]3C)nccc2cc1C(N)=O	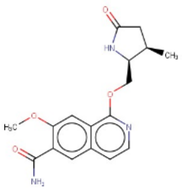
**IRAK4_46**	CC[C@H]1CC(=O)N[C@H]1COc1nccc2cc(C(N)=O)c(OC)cc12	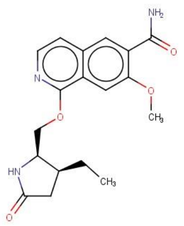
**IRAK4_47**	CCC[C@H]1CC(=O)N[C@H]1COc1nccc2cc(C(N)=O)c(OC)cc12	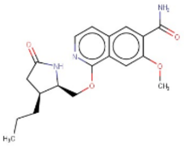

**IRAK4_48**	COC[C@H]1CC(=O)N[C@H]1COc1nccc2cc(C(N)=O)c(OC)cc12	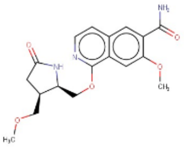
**IRAK4_49**	COc1cc2c(OC[C@H]3NC(=O)[C@@H](C)[C@H]3C)nccc2cc1C(N)=O	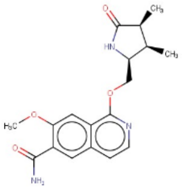
**IRAK4_50**	COc1cc2c(OC[C@H]3NC(=O)[C@H](C)[C@H]3C)nccc2cc1C(N)=O	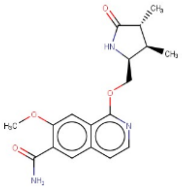
**IRAK4_51**	COc1cc2c(OC[C@H]3NC(=O)[C@@H](F)[C@H]3C)nccc2cc1C(N)=O	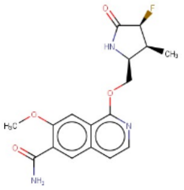
**IRAK4_52**	COc1cc2c(OC[C@H]3NC(=O)[C@H](F)[C@H]3C)nccc2cc1C(N)=O	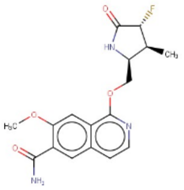
**IRAK4_53**	CC[C@H]1[C@@H](COc2nccc3cc(C(N)=O)c(OC)cc23)NC(=O)[C@H]1F	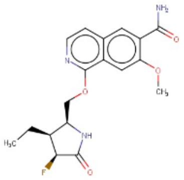
**IRAK4_54**	CC[C@H]1[C@@H](COc2nccc3cc(C(N)=O)c(OC)cc23)NC(=O)[C@@H]1F	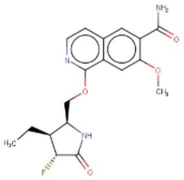
**IRAK4_55**	COc1cc2c(OC[C@H]3NC(=O)[C@@H]4C[C@H]34)nccc2cc1C(N)=O	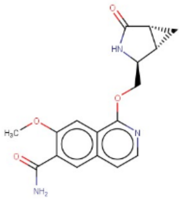
**IRAK4_56**	COc1cc2c(OC[C@H]3NC(=O)[C@@H]4[C@@H](C)[C@H]34)nccc2cc1C(N)=O	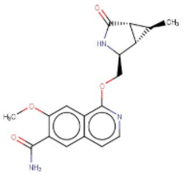
**IRAK4_57**	COc1cc2c(OC[C@H]3NC(=O)[C@]4(F)[C@@H](C)[C@H]34)nccc2cc1C(N)=O	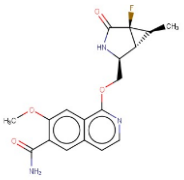

**IRAK4_58**	CC(C)Oc1ccccc1C(=O)N	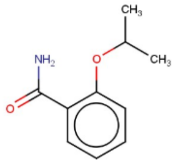
**IRAK4_59**[*]	CC(C)N1C(NC(=O)C2=CC=CC(=C2)[N+]([O-])=O)=NC2=C1C=CC=C2	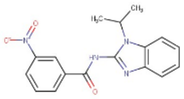
**IRAK4_60**[*]	C1CC(CCN1)NC1=NC=CC(=N1)C1=CN=C2C=CC=CN12	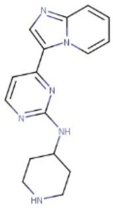
**IRAK4_61**[*]	CN(C)C(=O)C1=CN2C(C=C1)=NC=C2C1=NC(NC2CCNCC2)=CC=C1	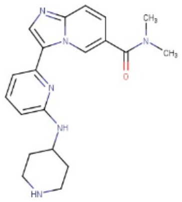
**IRAK4_62**[*]	CN(C)[C@H]1CC[C@@H](CC1)NC1=C2C(NC=C2C#N)=NC=N1	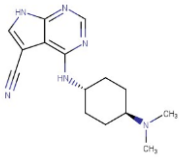
**IRAK4_63**[*]	CN(C)[C@H]1CC[C@@H](CC1)NC1=C2C(F)=CNC2=NC=N1	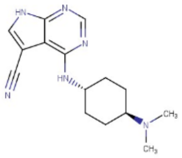
**IRAK4_64**[*]	CN(C)[C@H]1CC[C@@H](CC1)NC1=C2C=CNC2=NC(NC2=CC=CC=C2)=N1	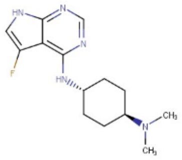
**IRAK4_65**[*]	NC1=NC(N[C@@H]2C[C@H](CO)[C@@H](O)[C@H]2O)=C(C=N1)C1=NC2=C(S1)C=CN=C2	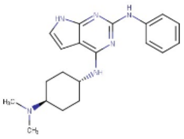
**IRAK4_66**[*]	NC1=NC(N[C@@H]2C[C@H](CO)[C@@H](O)[C@H]2O)=C(C=N1)C1=CN=C2C=CC=CN12	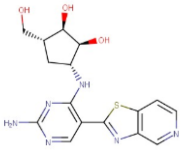
**IRAK4_67**[*]	CN1C=C(NC(=O)C2=C3N=CC=CN3N=C2)C(=N1)C(N)=O	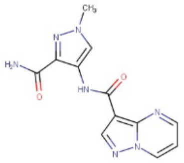
**IRAK4_68**[*]	CN(C)C1=NC(N[C@@H]2C[C@H](CO)[C@@H](O)[C@H]2O)=C(C2=NC3=C(S2)C=CC=C3)C(=O)N1	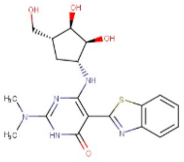

**IRAK4_69***	N#CC1=C(NC2=CC=CC(=N2)C2=CN=C3C=CC=CN23)C=CC=C1	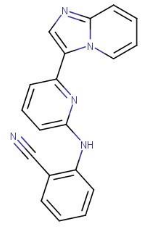
**IRAK4_70***	C1CC(CCN1)NC1=CC=CC(=N1)C1=CN=CC2=CC=CC=C12	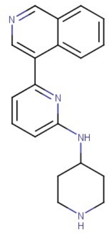

*** Test Set**

Table SI14. IRAK4 training set composition. The table shows the experimental activity of each molecule and the predicted activity of the model for each molecule, with the relative error of fitting

ID	Experimental	Predicted (CV)	Prediction Error
**IRAK4_01**	6.854	6.987	-0.133
**IRAK4_02**	5.824	5.660	0.164
**IRAK4_03**	5.796	6.338	-0.542
**IRAK4_04**	5.620	6.200	-0.580
**IRAK4_05**	5.201	6.408	-1.207
**IRAK4_06**	6.387	6.899	-0.512
**IRAK4_07**	5.046	6.276	-1.230
**IRAK4_08**	8.301	6.002	2.299
**IRAK4_09**	6.194	5.731	0.463
**IRAK4_10**	7.886	7.390	0.496
**IRAK4_11**	8.301	7.853	0.448
**IRAK4_12**	6.854	7.501	-0.647
**IRAK4_13**	7.004	6.600	0.404
**IRAK4_14**	7.959	7.251	0.708
**IRAK4_15**	8.523	7.880	0.643
**IRAK4_16**	8.222	7.517	0.705
**IRAK4_17**	6.561	7.199	-0.638
**IRAK4_18**	7.194	7.443	-0.249
**IRAK4_19**	7.796	7.512	0.284
**IRAK4_20**	8.222	7.978	0.244
**IRAK4_21**	6.721	7.162	-0.441
**IRAK4_22**	7.357	7.882	-0.525
**IRAK4_23**	8.699	7.284	1.415
**IRAK4_24**	8.523	8.833	-0.310
**IRAK4_25**	6.854	8.372	-1.518
**IRAK4_26**	8.523	7.956	0.567
**IRAK4_27**	8.301	7.702	0.599
**IRAK4_28**	8.046	8.388	-0.342
**IRAK4_29**	8.699	8.525	0.174
**IRAK4_30**	6.244	7.769	-1.525
**IRAK4_31**	7.481	6.856	0.625
**IRAK4_32**	8.398	8.129	0.269

**IRAK4_33**	7.658	7.519	0.139
**IRAK4_34**	7.252	7.944	-0.692
**IRAK4_35**	8.680	8.261	0.419
**IRAK4_36**	7.143	8.341	-1.198
**IRAK4_37**	6.836	7.320	-0.484
**IRAK4_38**	6.917	6.068	0.849
**IRAK4_39**	8.337	8.353	-0.016
**IRAK4_40**	8.119	7.949	0.170
**IRAK4_41**	7.622	7.601	0.021
**IRAK4_42**	8.229	7.754	0.475
**IRAK4_43**	6.076	8.175	-2.099
**IRAK4_44**	8.886	8.923	-0.037
**IRAK4_45**	8.569	8.676	-0.107
**IRAK4_46**	9.301	8.527	0.774
**IRAK4_47**	8.886	8.650	0.236
**IRAK4_48**	7.917	8.762	-0.845
**IRAK4_49**	7.535	8.704	-1.169
**IRAK4_50**	8.432	7.644	0.788
**IRAK4_51**	9.523	9.126	0.397
**IRAK4_52**	8.721	8.171	0.550
**IRAK4_53**	9.699	8.157	1.542
**IRAK4_54**	10.000	8.260	1.740
**IRAK4_55**	8.319	9.331	-1.012
**IRAK4_56**	8.620	7.836	0.784
**IRAK4_57**	9.222	8.854	0.368
**IRAK4_58**	7.259	8.230	-0.971

3-D QSAR model building. For conformation analysis, 70 conformations through Balloon method were performed and a molecular alignment with Shaep method was executed (Figure SI10). Being a Ligand-Based approach, the alignment rules can be multiple, such as the most/least active molecule, the most/least polar molecule, etc. (Table SI15) So, all rules were applied and for each one a 3-D QSAR model with default settings has built. The best alignment found on the lowest LogP molecule (ID molecule: IRAK4_55).

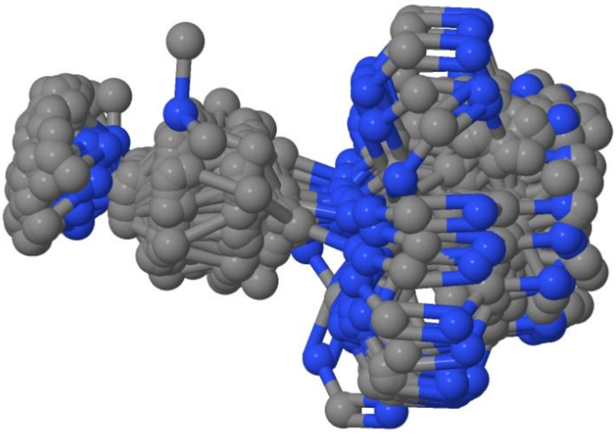

A

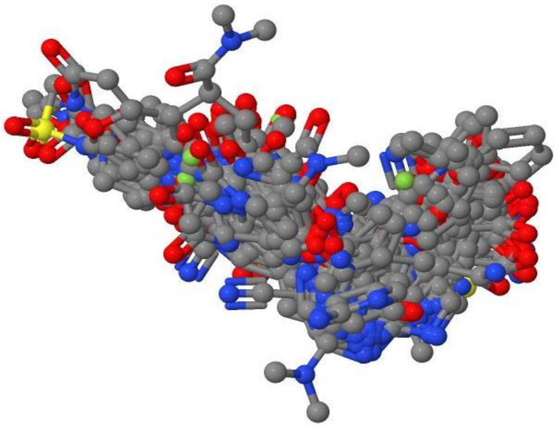

B

Figure SI10. IRAK4 alignment rules. **(A)** example of conformations obtained for the compound IRAK4 12. **(B)** Training set aligned on the template molecule IRAK4_55.

Table SI15. IRAK4 3-D QSAR coefficients obtained for the different alignment rules.

Alignment Rules	Probe Atom	q^2 STE	q^2 ELE	q^2 BOTH	Max ONPC	Grid Spacing	Grid Extension	CutOff	Min. Sigma	CV Type
**Least Active**	H	0.156	0.249	0.251	8	1.0	5	30.0	0.05	LOO
**Most Active**	O.3	0.075	0.367	0.436	8	1.0	5	30.0	0.05	LOO
**Heaviest**	N.3	0.293	0.174	0.357	8	1.0	5	30.0	0.05	LOO
**Longest**	H	0.213	0.374	0.352	8	1.0	5	30.0	0.05	LOO
**Most Flexible**	C.3	0.193	0.197	0.286	8	1.0	5	30.0	0.05	LOO
**Most Rigid**	C.3	0.193	0.197	0.286	8	1.0	5	30.0	0.05	LOO
**Least Polar**	H	0.107	0.323	0.343	8	1.0	5	30.0	0.05	LOO
**Most Polar**	C.3	0.228	0.188	0.268	8	1.0	5	30.0	0.05	LOO
**Highest MR**	O.3	-0.064	0.198	0.206	8	1.0	5	30.0	0.05	LOO
**Lowest MR**	H	-0.026	0.341	0.347	8	1.0	5	30.0	0.05	LOO
**Highest HA**	C.3	0.038	0.247	0.312	8	1.0	5	30.0	0.05	LOO
**Lowest HA**	C.3	0.038	0.247	0.312	8	1.0	5	30.0	0.05	LOO
**Highest HD**	O.3	0.279	0.186	0.233	8	1.0	5	30.0	0.05	LOO
**Lowest HD**	O.3	0.279	0.186	0.233	8	1.0	5	30.0	0.05	LOO
**Highest LogP**	N.3	-0.041	0.213	0.261	8	1.0	5	30.0	0.05	LOO
**Lowest LogP**	O.3	0.315	0.222	0.466	8	1.0	5	30.0	0.05	LOO

For each alignment, a construction model was tested with the same option value for Grid spacing, Grid extension, CutOff, Min. Sigma and CV Type, changing only the probe atom to find the best result indicating in which type of alignment start building the model. The best model is highlighted in yellow

The best model obtained was optimized in terms of probes atom, grid spacing, grid extension and so on to find the best 3-D QSAR model optimized (Table SI16). The best probe with best energy interaction is Oxygen (O.3) and it shows an electrostatic and steric q^2 value of 0.444, a fitting represented by r^2 of 0.979, SDEC of 0.162 and SDEP of 0.837 considering 6 optimal numbers of principal components (ONPC) . The best outcome setting is obtained with grid spacing 1.0 Å, grid extension 5 Å, minimum sigma 2.0 and cutoff energy of 25 kcal/mol. Later, on the best model has been performed an LSO validation in addition to LOO, in order to assess the model stability. In the end, a Y-scrambling (Y_S) validation has been carried out to verify if the model was affected by the fortuitous correlation and the feedback was positive (Tables SI16- SI17 and Figure SI11).

Table SI16. IRAK4 3-D QSAR models optimization by varying the pretreatment parameters on the best alignment rule.

Probe Atom	q^2 STE	q^2 ELE	q^2 BOTH	ONPC	Grid Spacing	Grid Extension	CutOff	Min. Sigma	CV Type	r^2	SDEC	SDEP	r^2 YS	q^2 YS	SDEP YS
O.3	0.315	0.222	0.466	8	1.0	5	30.0	0.05	LOO	0.995	0.079	0.820	/	/	/
O.3	0.215	0.330	0.443	8	1.1	5	30.0	0.05	LOO	/	/	/	/	/	/
O.3	0.187	0.345	0.458	8	1.2	5	30.0	0.05	LOO	/	/	/	/	/	/
O.3	0.297	0.217	0.405	8	1.3	5	30.0	0.05	LOO	/	/	/	/	/	/
O.3	0.095	0.273	0.295	8	1.4	5	30.0	0.05	LOO	/	/	/	/	/	/
O.3	0.183	0.273	0.337	8	1.5	5	30.0	0.05	LOO	/	/	/	/	/	/
O.3	0.283	0.214	0.444	8	1.6	5	30.0	0.05	LOO	/	/	/	/	/	/
O.3	0.045	0.346	0.470	8	1.7	5	30.0	0.05	LOO	/	/	/	/	/	/
O.3	0.192	0.275	0.386	8	1.8	5	30.0	0.05	LOO	/	/	/	/	/	/
O.3	0.146	0.216	0.271	8	1.9	5	30.0	0.05	LOO	/	/	/	/	/	/
O.3	-0.036	0.273	0.388	8	2.0	5	30.0	0.05	LOO	/	/	/	/	/	/
O.3	0.026	0.461	0.308	8	2.1	5	30.0	0.05	LOO	/	/	/	/	/	/
O.3	0.240	0.217	0.439	8	2.2	5	30.0	0.05	LOO	/	/	/	/	/	/
O.3	0.149	0.212	0.337	8	2.3	5	30.0	0.05	LOO	/	/	/	/	/	/
O.3	-0.077	0.229	0.355	8	2.4	5	30.0	0.05	LOO	/	/	/	/	/	/
O.3	0.044	0.234	0.318	8	2.5	5	30.0	0.05	LOO	/	/	/	/	/	/
O.3	0.173	0.216	0.301	8	2.6	5	30.0	0.05	LOO	/	/	/	/	/	/
O.3	-0.162	0.223	0.248	8	2.7	5	30.0	0.05	LOO	/	/	/	/	/	/
O.3	-0.001	0.259	0.252	8	2.8	5	30.0	0.05	LOO	/	/	/	/	/	/
O.3	-0.055	0.212	0.241	8	2.9	5	30.0	0.05	LOO	/	/	/	/	/	/
O.3	0.111	0.260	0.328	8	3.0	5	30.0	0.05	LOO	/	/	/	/	/	/
O.3	0.315	0.222	0.466	8	1.0	5	30.0	0.05	LOO	0.995	0.079	0.820	/	/	/
O.3	0.302	0.223	0.462	8	1.0	5	20.0	0.05	LOO	/	/	/	/	/	/
O.3	0.310	0.221	0.469	8	1.0	5	25.0	0.05	LOO	/	/	/	/	/	/
O.3	0.314	0.223	0.460	8	1.0	5	35.0	0.05	LOO	/	/	/	/	/	/
O.3	0.308	0.223	0.459	8	1.0	5	40.0	0.05	LOO	/	/	/	/	/	/
O.3	0.310	0.221	0.469	8	1.0	5	25.0	0.05	LOO	0.995	0.079	0.818	/	/	/
O.3	0.310	0.221	0.468	8	1.0	6	25.0	0.05	LOO	/	/	/	/	/	/
O.3	0.310	0.221	0.468	8	1.0	7	25.0	0.05	LOO	/	/	/	/	/	/
O.3	0.310	0.222	0.467	8	1.0	8	25.0	0.05	LOO	/	/	/	/	/	/
O.3	0.310	0.222	0.466	8	1.0	9	25.0	0.05	LOO	/	/	/	/	/	/
O.3	0.310	0.222	0.466	8	1.0	10	25.0	0.05	LOO	/	/	/	/	/	/
O.3	0.310	0.221	0.469	8	1.0	5	25.0	0.05	LOO	0.995	0.079	0.818	/	/	/

O.3	0.310	0.221	0.469	8	1.0	5	25.0	0.10	LOO	/	/	/	/	/	/
O.3	0.310	0.221	0.469	8	1.0	5	25.0	0.15	LOO	/	/	/	/	/	/
O.3	0.310	0.221	0.469	8	1.0	5	25.0	0.20	LOO	/	/	/	/	/	/
O.3	0.310	0.221	0.469	8	1.0	5	25.0	0.25	LOO	/	/	/	/	/	/
O.3	0.310	0.221	0.469	8	1.0	5	25.0	0.30	LOO	/	/	/	/	/	/
O.3	0.310	0.221	0.469	8	1.0	5	25.0	0.35	LOO	/	/	/	/	/	/
O.3	0.310	0.221	0.469	8	1.0	5	25.0	0.40	LOO	/	/	/	/	/	/
O.3	0.310	0.221	0.469	8	1.0	5	25.0	0.45	LOO	/	/	/	/	/	/
O.3	0.310	0.221	0.469	8	1.0	5	25.0	0.50	LOO	/	/	/	/	/	/
O.3	0.310	0.221	0.469	8	1.0	5	25.0	0.55	LOO	/	/	/	/	/	/
O.3	0.310	0.221	0.469	8	1.0	5	25.0	0.60	LOO	/	/	/	/	/	/
O.3	0.310	0.221	0.469	8	1.0	5	25.0	0.65	LOO	/	/	/	/	/	/
O.3	0.310	0.221	0.469	8	1.0	5	25.0	0.70	LOO	/	/	/	/	/	/
O.3	0.310	0.221	0.469	8	1.0	5	25.0	0.75	LOO	/	/	/	/	/	/
O.3	0.310	0.221	0.469	8	1.0	5	25.0	0.80	LOO	/	/	/	/	/	/
O.3	0.310	0.221	0.469	8	1.0	5	25.0	0.85	LOO	/	/	/	/	/	/
O.3	0.310	0.221	0.469	8	1.0	5	25.0	0.90	LOO	/	/	/	/	/	/
O.3	0.310	0.221	0.469	8	1.0	5	25.0	0.95	LOO	/	/	/	/	/	/
O.3	0.310	0.221	0.469	8	1.0	5	25.0	1.00	LOO	/	/	/	/	/	/
O.3	0.310	0.221	0.469	8	1.0	5	25.0	1.05	LOO	/	/	/	/	/	/
O.3	0.310	0.221	0.469	8	1.0	5	25.0	1.10	LOO	/	/	/	/	/	/
O.3	0.310	0.221	0.469	8	1.0	5	25.0	1.15	LOO	/	/	/	/	/	/
O.3	0.310	0.221	0.469	8	1.0	5	25.0	1.20	LOO	/	/	/	/	/	/
O.3	0.310	0.221	0.469	8	1.0	5	25.0	1.25	LOO	/	/	/	/	/	/
O.3	0.310	0.221	0.469	8	1.0	5	25.0	1.30	LOO	/	/	/	/	/	/
O.3	0.311	0.221	0.469	8	1.0	5	25.0	1.35	LOO	/	/	/	/	/	/
O.3	0.310	0.220	0.469	8	1.0	5	25.0	1.40	LOO	/	/	/	/	/	/
O.3	0.311	0.220	0.469	8	1.0	5	25.0	1.45	LOO	/	/	/	/	/	/
O.3	0.311	0.220	0.469	8	1.0	5	25.0	1.50	LOO	/	/	/	/	/	/
O.3	0.311	0.220	0.469	8	1.0	5	25.0	1.55	LOO	/	/	/	/	/	/
O.3	0.311	0.220	0.469	8	1.0	5	25.0	1.60	LOO	/	/	/	/	/	/
O.3	0.311	0.219	0.470	8	1.0	5	25.0	1.65	LOO	/	/	/	/	/	/
O.3	0.311	0.219	0.470	8	1.0	5	25.0	1.70	LOO	/	/	/	/	/	/
O.3	0.311	0.219	0.470	8	1.0	5	25.0	1.75	LOO	/	/	/	/	/	/
O.3	0.311	0.219	0.470	8	1.0	5	25.0	1.80	LOO	/	/	/	/	/	/
O.3	0.312	0.218	0.470	8	1.0	5	25.0	1.85	LOO	/	/	/	/	/	/
O.3	0.311	0.218	0.470	8	1.0	5	25.0	1.90	LOO	/	/	/	/	/	/
O.3	0.312	0.218	0.471	8	1.0	5	25.0	1.95	LOO	/	/	/	/	/	/
O.3	0.312	0.218	0.471	8	1.0	5	25.0	2.00	LOO	0.995	0.078	0.816	/	/	/
O.3	0.313	0.221	0.469	8	1.0	5	25.0	3.00	LOO	/	/	/	/	/	/
O.3	0.312	0.215	0.469	8	1.0	5	25.0	4.00	LOO	/	/	/	/	/	/
O.3	0.311	0.291	0.456	8	1.0	5	25.0	2.00	LSO	0.995	0.078	0.827	/	/	/
O.3	0.312	0.218	0.471	6	1.0	5	25.0	2.00	LOO	0.979	0.162	0.830	0.938	-0.304	1.280
O.3	0.316	0.276	0.453	6	1.0	5	25.0	2.00	LSO	0.979	0.162	0.830	0.938	-0.276	1.265

The best models (LOO and LSO) are highlighted in yellow.

Table SI17. IRAK4 3-D QSAR model metrics.

Fields	ONPC[a]	r^2	SDEC[b]	q^2		SDEP[c]		r^2_{YS}	q^2_{YS}	
				LOO[d]	LSO[e]	LOO	LSO		LOO	LSO
**Steric**	6	0.985	0.138	0.291	0.312	0.945	0.930	0.969	-0.304	-0.298
**Electrostatic**	4	0.831	0.461	0.217	0.266	0.993	0.961	0.629	-0.366	-0.477
**Steric/Electrostatic**	6	0.979	0.162	0.444	0.453	0.837	0.830	0.938	-0.304	-0.276

**Optimal Settings**	
**Probe Atom**	O.3
**Grid Spacing**	1
**Grid Extension**	5
**Minimum Sigma**	2
**Max/Min Energy of Cutoff Value**	25

[a] ONPC: Optimal Number of Principal Components
[b] SDEP: Cross-validated Standard Deviation Error Prediction
[c] SDEC: Standard Deviation Error Calculation
[d] LOO: Leave-One-Out
[e] LSO: Leave-Some-Out

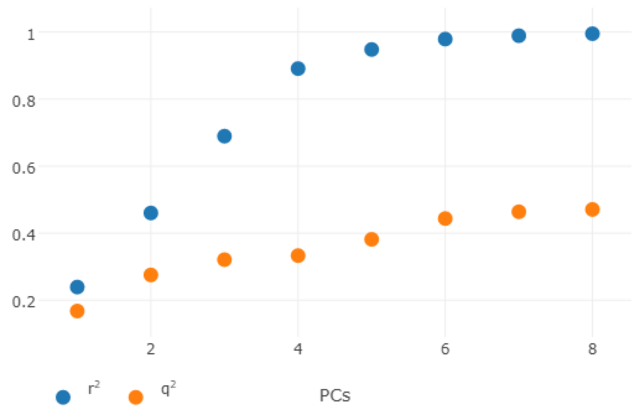

A

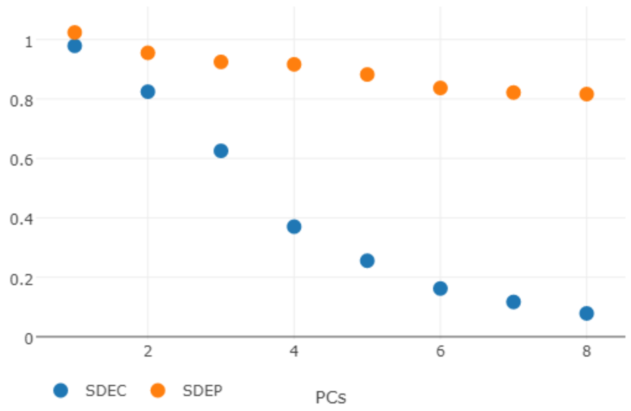

B

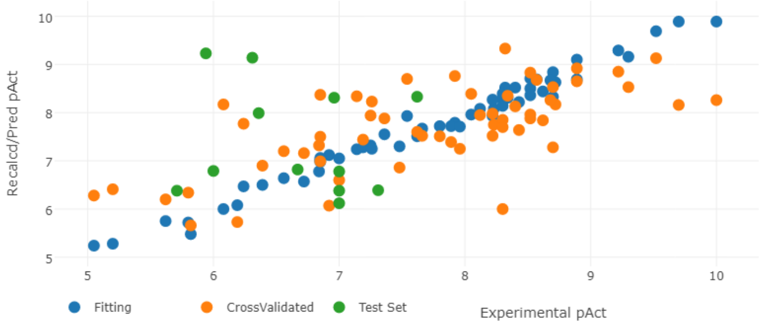

C

Figure SI11. Plots for IRAK4 dataset. **(A)** r^2 and q^2 versus the number of PCs; **(B)** SDEC and SDEP versus the number of PCs; **(C)** Recalculated and predicted versus the experimental activities.

Final model graphical inspection.

3-D QSAR contour maps generated using the "Coeff x Mean" (average activity contribution - AAC) option and overlapped to the most (IRAK4_54) and least (IRAK4_07) potent IRAK4 inhibitor were used to interpret the best CoMFA like model (Figure SI12). Positive and negative steric associated AAC are depicted by green and yellow polyhedrons as suggested in the original CoMFA article[29] (panels A and B of Figure SI12). In particular, green polyhedron was observable around the IRAK_07 fluorine atom indicating that a slight increase of steric hindrance in that position could lead to more potent derivatives (Figure SI12A). Further green polyhedron are visible around the main central fused bicycle (pyrrolopyrimidine) revealing that some substitution could provide more potent derivatives. Regarding the negatively associate steric contribution (yellow polyhedrons in Figure SI12B) the most important features to be avoided are the substitutions corresponding to the IRAK4_07 methyl and IRAK_54 carboxyamide at position C12 and C11 of the byciclic central moiety (pyrrolopyrimidine), respectively (Figure SI12 B). Regarding positive and negative electrostatic associated AAC to avoid any misunderstanding have been represented in blue and red meshed contour maps, respectively (panels C and D of Figure SI12). These maps clearly indicated that an electron-rich substituent is favorable for the activity (Figure SI12C), instead 2-pyrrolidone's ethyl group and -NH group are negatively influencing the inhibiting potency (Figure SI12D).

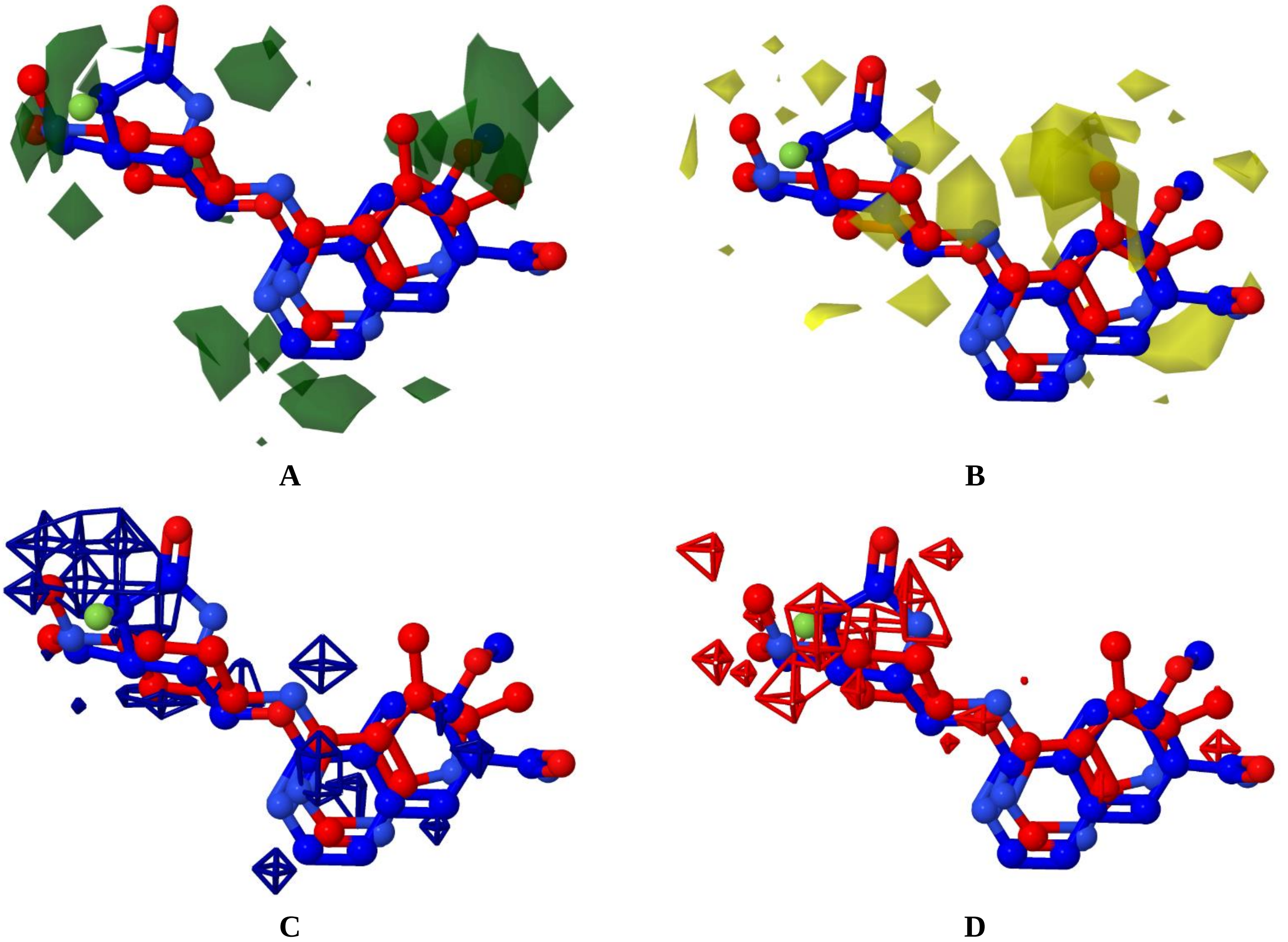

Figure SI12. (A) Least (in red) and most (in blue) active molecule's positive (in green) and negative (in orange) steric Coeff x Mean. (B) Least (in red) and most (in blue) active molecule's positive (in blue) and negative (in red) electrostatic Coeff x Mean.

Test set composition and predictive ability evaluation.

The predictive ability of the best model was then evaluated by mean of an external test set by randomly selecting 12 compounds from the ChEMBL database (https://www.ebi.ac.uk/chembl/) and subjecting them to all the procedure and getting the predicted activities (Table SI18). It must be noted however that, inasmuch as ChEMBL is a record of all published structures having any reported biological potency, the very poor accuracy of these predictions for these random structural choices is only to be expected.

Table SI18. IRAK4 test set experimental and predicted activity by the final 3-D QSAR model.

ID	Experimental	Predicted	Error
**IRAK4_59**	6.000	6.793	-0.793
**IRAK4_60**	6.670	6.819	-0.149
**IRAK4_61**	7.310	6.385	0.925
**IRAK4_62**	7.000	6.123	0.877
**IRAK4_63**	7.000	6.376	0.624
**IRAK4_64**	7.000	6.780	0.220
**IRAK4_65**	7.620	8.328	-0.708
**IRAK4_66**	5.940	9.229	-3.289
**IRAK4_67**	6.960	8.306	-1.346

**IRAK4_68**	6.310	9.138	-2.828
**IRAK4_69**	6.360	7.986	-1.626
**IRAK4_70**	5.710	6.377	-0.667

4. Students works [M.V.]: Bromodomain–Containing Protein 4 (BRD4)

In this section is described the development of FB LB 3-D QSAR model based on the molecules described by *Keith F. McDaniel et al.*[86] The molecules were evaluated as ligand for BRD4.

Overview of the biological target and connected pathology. BRDs are structure evolutionarily conserved in different nuclear proteins and there are 46 human proteins known to contain bromodomains[86] BRD4 (Figure SI13) is a protein made up 1362 amino acids and results to have a molecular weight of 200 kD. BRD4 belongs to the BET subgroup of the bromodomain superfamily and contains 2 bromodomains and a conserved Extra-Terminal (ET)-domain.[87] Despite extensive sequence variations, all BRDs share a conserved fold that includes a bundle of four left-handed helices (αZ, αA, αB, αC), linked by loop regions of varying length (ZA and BC rings) that delineate the site of binding of acetyl-lysine (KAc) and determine the binding specificity. Precisely this affinity with KAc becomes an object of interest for the development of drugs as competitive inhibitors[88]. BET-proteins have been implicated in a number of pathological pathways as for example transcription, chromatin remodelling, gene splicing, protein scaffolding, and signal transduction, and thus play key roles in cell proliferation and division and this is why are considered promising pharmacological targets [89]. The BET family represents an entire group, composed of four members: BRD2, BRD3, BRD4 and BRDT. Each contains two highly conserved N-terminal bromodomains (BD1 and BD2), an ET domain, and a C-terminal domain (CTD). Despite their sequence similarity, BD1 and BD2 appear to have distinct functions due to their interactions with different lysine-acetylated histones (e.g., H3 and H4) or with transcriptional proteins[88]. An essential role of BRD4 in cell proliferation and cancer growth has been reported in several recent studies. The chemical inhibition of BRD4 could have interesting desirable biological findings as antitumor and anti-inflammatory properties.[90-92]. All known BRD4 inhibitors target the KAc recognition site, in particular through H-bonding interactions with a conserved Asn433 residue. The basic interaction that every drug for BRD4 must respect to maintain affinity with the binding site is the strong hydrogen bond with Asp433, the hydrophobic interactions with conserved residues Trp374, Pro375 and Phe376 (WPT region) [88] It was observed that the inhibition of BRD4 down-regulates MYC oncogenic transcription factors in several tumour cell lines[93]. Efforts are underway to develop chemically diverse and very potent BRD4 inhibitors such as new cancer therapies. Currently, eight different BRD4 inhibitors have entered phase I studies for the treatment of liquid and solid tumours[86].

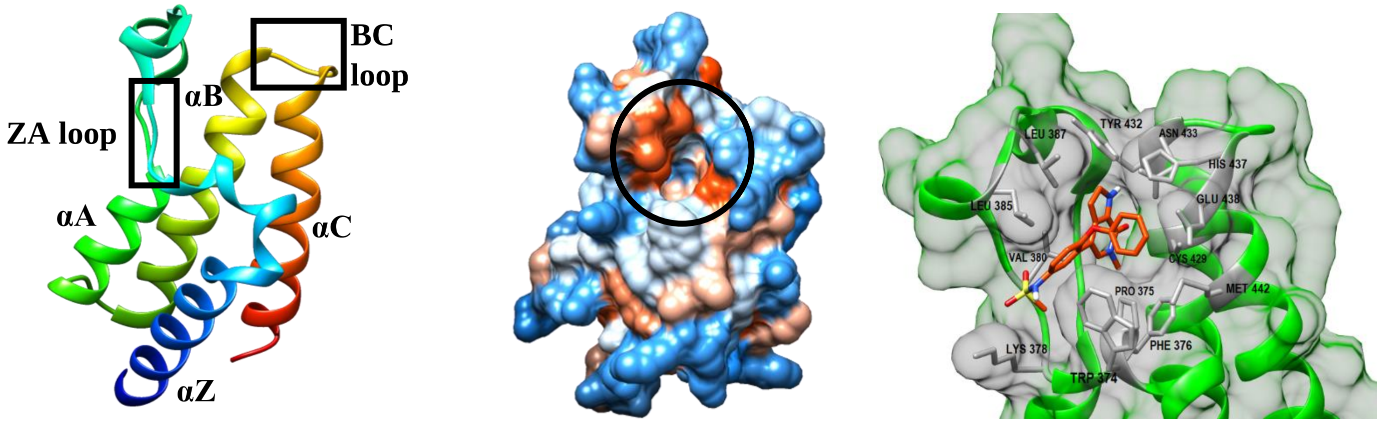

A B C

Figure SI13. Cartoon representation of BRD4 three-dimensional structure. (A) The four α chains are indicated in different colors: αA in green, αB in yellow; BC in orange, αZ in blue. Furthermore, the two black rectangles show the two rings that delimit the binding site. (B) Surface representation of hydrophobic and hydrophilic sites of the protein. The black circle indicates the core. (C) Cartoon, stick and surface representation of the A-1359643 into the hydrophobic binding site of BRD4 (C). For figure generation, the data available in the PDB entry code 5UVX was used.

Training set composition. The training set consists of 45 molecules reported by *Keith F. McDaniel et al* [86] (Table SI19). These compounds, characterized by a pyridone core linked to a diaryl ether scaffold showing binding

ability against BRD4. In Table SI20 are shown the experimental activity of each molecule and the predicted activity of the model for each molecule, with the relating error.

Table SI19. IDs, SMILES and 2-D structures of the BRD4 datasets.[7]

ID	SMILES	2-D Chemical Structure
**BRD4_01**	C(N(C)C(=O)C=C1)=C1c2c(Oc3ccccc3)cccc2	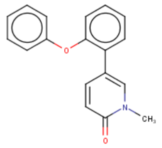
**BRD4_02**	N(C1=O)([H])C=C(c2c(Oc3ccccc3)cccc2)C=C1C	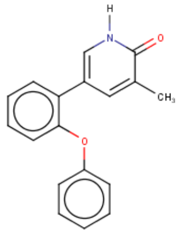
**BRD4_03**	N1(C)C(=O)C(N)=CC(c2ccccc2Oc3ccccc3)=C1	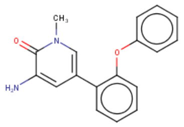
**BRD4_04**	C(N(C)C(=O)c([nH]cc1)c12)=C2c3c(Oc4ccccc4)cccc3	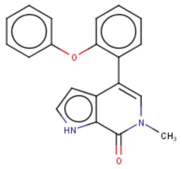
**BRD4_05**	C1(=O)N(C)C=C(c2c(Oc3ccccc3)ccc(NS(C)(=O)=O)c2)c(c4)c1[nH]c4	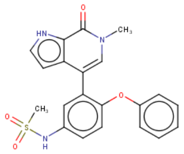
**BRD4_06**	c1(NS(=O)(=O)CC)cc(C2=CN(C)C(=O)c([nH]cc3)c23)c(Oc4ccccc4)cc1	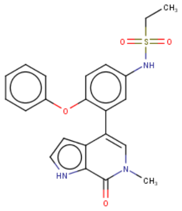
**BRD4_07**	C1(=O)N(C)C=C(c2c(Oc3ccccc3)ccc(NS(CC(F)(F)F)(=O)=O)c2)c(c4)c1[nH]c4	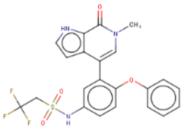
**BRD4_08**	c1(NS(=O)(=O)N(C)C)cc(C2=CN(C)C(=O)c([nH]cc3)c23)c(Oc4ccccc4)cc1	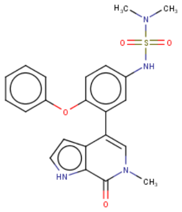
**BRD4_09**	C1(=O)N(C)C=C(c2c(Oc3ccccc3)ncc(NS(=O)(=O)C)c2)c(c4)c1[nH]c4	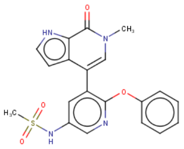

**BRD4_10**	C1(=O)N(C)C=C(c(c(ccc2NC(C)=O)Oc3ccccc3)c2)c(c4)c1n([H])c4	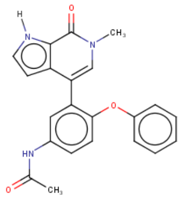
**BRD4_11**	c1(C(=O)N)cc(C2=CN(C)C(=O)c([nH]cc3)c23)c(Oc4ccccc4)cc1	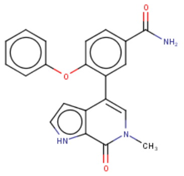
**BRD4_12**	c1(C(=O)NCC)cc(C2=CN(C)C(=O)c([nH]cc3)c23)c(Oc4ccccc4)cc1	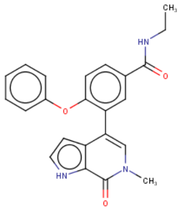
**BRD4_13**	N1(C)C(=O)c([nH]cc2)c2C(c3c(Oc4ccccc4)ccc(S(C)(=O)=O)c3)=C1	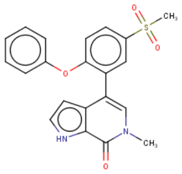
**BRD4_14**	N1(C)C(=O)c([nH]cc2)c2C(c3c(Oc4ccccc4)ccc(S(N)(=O)=O)c3)=C1	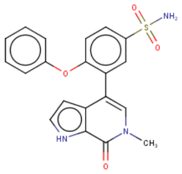
**BRD4_15**	N1(C)C(=O)c([nH]cc2)c2C(c3c(Oc4ccc(Cl)cc4)ccc(NS(=O)(=O)CC)c3)=C1	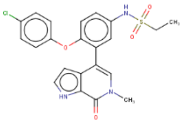
**BRD4_16**	c1(NS(=O)(=O)C)cc(C2=CN(C)C(=O)c([nH]cc3)c23)c(Oc4ccc(C#N)cc4)cc1	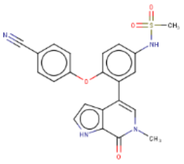
**BRD4_17**	N1(C)C(=O)c([nH]cc2)c2C(c3c(Oc4ccc(C(F)(F)F)cc4)ccc(NS(=O)(=O)CC)c3)=C1	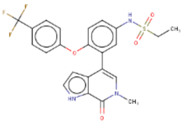
**BRD4_18**	c1(NS(=O)(=O)C)cc(C2=CN(C)C(=O)c([nH]cc3)c23)c(Oc4c(C(F)(F)F)cccc4)cc1	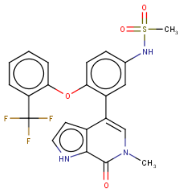
**BRD4_19**	c1(NS(=O)(=O)C)cc(C2=CN(C)C(=O)c([nH]cc3)c23)c(Oc4c(Cl)cc(F)cc4)cc1	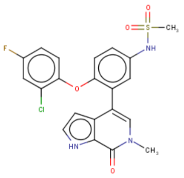
**BRD4_20**	c1(NS(=O)(=O)C)cc(C2=CN(C)C(=O)c([nH]cc3)c23)c(Oc4c(F)cc(F)cc4)cc1	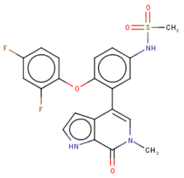

**BRD4_21**	c1(NS(=O)(=O)CC)cc(C2=CN(C)C(=O)c([nH]cc3)c23)c(Oc4c(F)cc(F)cc4)cc1	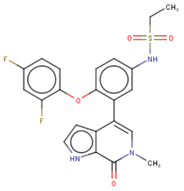
**BRD4_22**	c1(NS(=O)(=O)CC)cc(C2=CN(C)C(=O)c([nH]cc3)c23)c(Oc4c(F)cc(F)cc4F)cc1	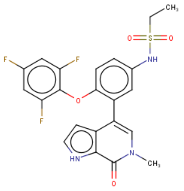
**BRD4_23**	c1(NS(=O)(=O)C)cc(C2=CN(C)C(=O)c([nH]cc3)c23)c(Oc4cnccc4)cc1	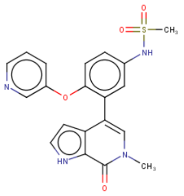
**BRD4_24**	c1(NS(=O)(=O)CC)cc(C2=CN(C)C(=O)c([nH]cc3)c23)c(Oc4cncnc4)cc1	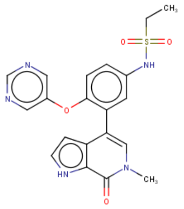
**BRD4_25**	c1(NS(=O)(=O)C)cc(C2=CN(C)C(=O)c([nH]cc3)c23)c(OCc4ccccc4)cc1	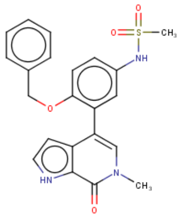
**BRD4_26**	c1(NS(=O)(=O)C)cc(C2=CN(C)C(=O)c([nH]cc3)c23)c(OC4CCCCC4)cc1	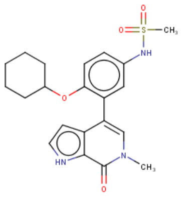
**BRD4_27**	c1(c(OC2CCOCC2)ccc(NS(C)(=O)=O)c1)C3=CN(C)C(=O)c([nH]cc4)c34	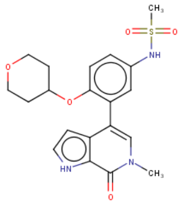
**BRD4_28**	c1(NS(=O)(=O)C)cc(C2=CN(C)C(=O)c([nH]cc3)c23)c(OC4CCC(F)(F)CC4)cc1	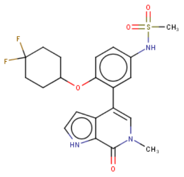
**BRD4_29**	c1(NS(=O)(=O)CC)cc(C2=CN(C)C(=O)c([nH]cc3)c23)c(OCC(C)(C)C)cc1	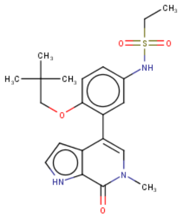
**BRD4_30**	N1(C)C(=O)c([nH]cc2)c2C(c3c(OC4CCC(N(C)C)CC4)ccc(NS(=O)(=O)CC)c3)=C1	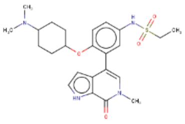

**BRD4_31**	N1(C)C(=O)c([nH]cc2)c2C(c3c(OC4CCN(C)CC4)ccc(NS(=O)(=O)CC)c3)=C1	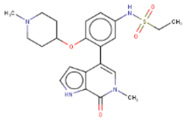
**BRD4_32**	c1(NS(=O)(=O)CCC)cc(C2=CN(C)C(=O)c([nH]cc3)c23)c(Oc4c(F)cc(F)cc4)cc1	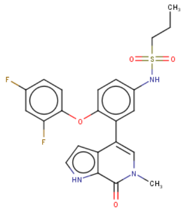
**BRD4_33**	N1(C)C(=O)c([nH]cc2)c2C(c3c(Oc4c(F)cc(F)cc4)ccc(NS(=O)(=O)C5CC5)c3)=C1	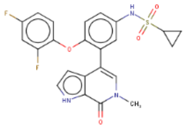
**BRD4_34**	N1(C)C(=O)c([nH]cc2)c2C(c3c(Oc4c(F)cc(F)cc4)ccc(NS(=O)(=O)CCOC)c3)=C1	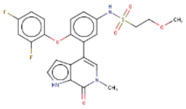
**BRD4_35**	N1(C)C(=O)c([nH]cc2)c2C(c3cc(NS(=O)(=O)CCN(C)C)ccc3Oc4ccc(F)cc4F)=C1	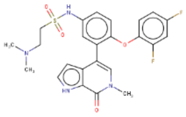
**BRD4_36**	c1(NS(=O)(=O)NC)cc(C2=CN(C)C(=O)c([nH]cc3)c23)c(Oc4c(F)cc(F)cc4)cc1	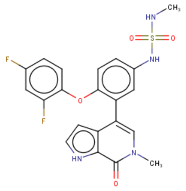
**BRD4_37**	c1(NC(=O)C)cc(C2=CN(C)C(=O)c([nH]cc3)c23)c(Oc4c(F)cc(F)cc4)cc1	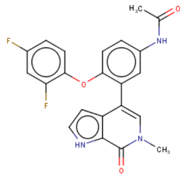
**BRD4_38**	N1(C)C(=O)c([nH]cc2)c2C(c3c(Oc4c(F)cc(F)cc4)ccc(S(=O)(=O)C)c3)=C1	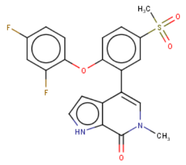
**BRD4_39**	N1(C)C(=O)c([nH]cc2)c2C(c3c(Oc4c(F)cc(F)cc4)ccc(S(=O)(=O)CC)c3)=C1	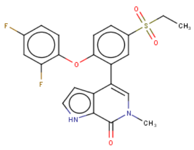
**BRD4_40**	c1c[nH]c(C(=O)N(C)C=C2c3c(Oc4c(F)cc(F)cc4)ncc(S(=O)(CC)=O)c3)c12	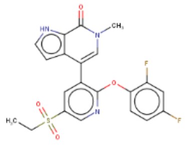
**BRD4_41**	N1(C)C(=O)c([nH]cc2)c2C(c3c(Oc4c(F)cc(F)cc4)ccc(S(=O)(=O)C(C)C)c3)=C1	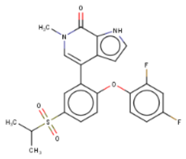
**BRD4_42**	c1c(C2=CN(C)C(=O)c([nH]cc3)c23)c(Oc4c(F)cc(F)cc4)ccc1S(=O)(=O)N	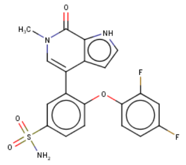

**BRD4_43**	C(N(C)C(=O)c([nH]cc1)c12)=C2c3c(Oc4c(F)cc(F)cc4)ccc(C(=O)N)c3	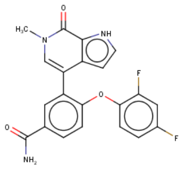
**BRD4_44**	N1(C)C(=O)c([nH]cc2)c2C(c3c(Oc4c(F)cc(F)cc4)ccc(CS(=O)(=O)C)c3)=C1	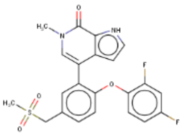
**BRD4_45**	N1(C)C(=O)c([nH]cc2)c2C(c3c(Oc4c(F)cc(F)cc4)ccc(CS(=O)(=O)CC)c3)=C1	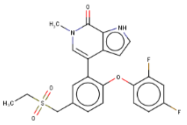
**BRD4_46**[*]	COC1=CC(=O)N(C)N=C1C1=C(OC2=CC=CC=C2)C=CC=C1	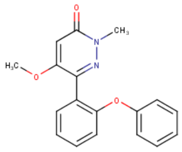
**BRD4_47**[*]	COC1=CC(=O)N(C)N=C1C1=C(OC2=CC=CC=C2)C=CC(N)=C1	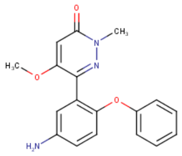
**BRD4_48**[*]	COC1=CC(=O)N(C)N=C1C1=C(OC2=CC=CC=C2)C=CC(NC(C)=O)=C1	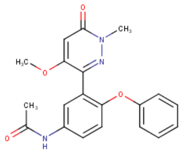
**BRD4_49**[*]	COC1=CC(=O)N(C)N=C1C1=C(OC2=CC=CC=C2)C=CC(NS(C)(=O)=O)=C1	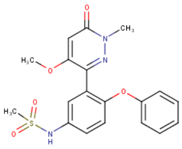
**BRD4_50**[*]	CCOC(=O)NC1=CC(=C(OC2=CC=CC=C2)C=C1)C1=NN(C)C(=O)C=C1OC	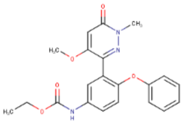
**BRD4_51**[*]	CCNC(=O)NC1=CC(=C(OC2=CC=CC=C2)C=C1)C1=NN(C)C(=O)C=C1OC	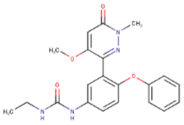
**BRD4_52**[*]	COC1=CC(=O)N(C)C=C1C1=C(OC2=CC=CC=C2)C=CC=C1	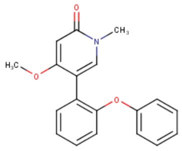
**BRD4_53**[*]	COC1=CC(=O)N(C)C=C1C1=C(OC2=CC=CC=C2)C=CC(NS(C)(=O)=O)=C1	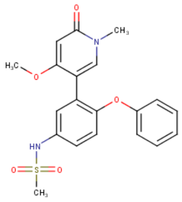
**BRD4_54**[*]	CCS(=O)(=O)NC1=CC(=C(OC2=CC=CC=C2)C=C1)C1=CN(C)C(=O)C=C1OC	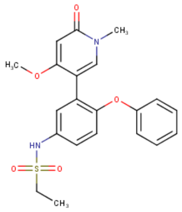

**BRD4_55***	CCOC1=CC(=O)N(C)C=C1C1=C(OC2=CC=CC=C2)C=CC(NS(=O)(=O)CC)=C1	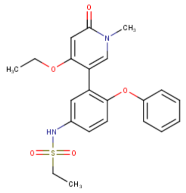
**BRD4_56***	CCOC1=CC(=O)N(C)C=C1C1=C(OC2=CC=C(F)C=C2)C=CC(NS(=O)(=O)CC)=C1	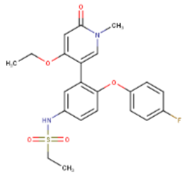
**BRD4_57***	CCOC1=CC(=O)N(C)C=C1C1=C(OC2=CC=C(Cl)C=C2)C=CC(NS(=O)(=O)CC)=C1	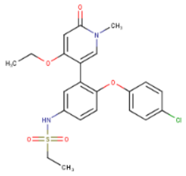

* **Test Set**

Table SI20. BRD4 training set composition. The table shows the experimental activity of each molecule and the predicted activity of the model for each molecule, with the relative error of fitting

ID	Experimental	Predicted (CV)	Prediction Error
**BRD4_01**	6.051	5.703	0.348
**BRD4_02**	5.770	6.067	-0.297
**BRD4_03**	5.523	6.433	-0.910
**BRD4_04**	7.319	6.999	0.320
**BRD4_05**	8.620	8.348	0.272
**BRD4_06**	8.824	8.176	0.648
**BRD4_07**	8.357	8.529	-0.172
**BRD4_08**	8.538	7.628	0.910
**BRD4_09**	8.387	8.076	0.311
**BRD4_10**	8.009	7.650	0.359
**BRD4_11**	7.854	7.203	0.651
**BRD4_12**	7.495	7.949	-0.454
**BRD4_13**	8.237	7.879	0.358
**BRD4_14**	8.444	7.582	0.862
**BRD4_15**	8.469	7.751	0.718
**BRD4_16**	8.027	8.059	-0.032
**BRD4_17**	8.046	8.247	-0.201
**BRD4_18**	8.194	8.121	0.073
**BRD4_19**	8.509	8.224	0.285
**BRD4_20**	8.347	8.319	0.028
**BRD4_21**	8.824	7.926	0.898
**BRD4_22**	7.921	8.113	-0.192
**BRD4_23**	8.201	7.885	0.316
**BRD4_24**	7.495	8.087	-0.592
**BRD4_25**	7.167	8.214	-1.047
**BRD4_26**	8.432	8.121	0.311
**BRD4_27**	7.167	7.833	-0.666
**BRD4_28**	7.721	7.842	-0.121
**BRD4_29**	7.420	7.813	-0.393

**BRD4_30**	7.585	6.977	0.608
**BRD4_31**	6.367	6.343	0.024
**BRD4_32**	8.000	8.168	-0.168
**BRD4_33**	8.036	7.874	0.162
**BRD4_34**	7.886	8.058	-0.172
**BRD4_35**	6.971	7.461	-0.490
**BRD4_36**	7.921	7.851	0.070
**BRD4_37**	7.319	7.884	-0.565
**BRD4_38**	7.824	7.282	0.542
**BRD4_39**	7.553	8.138	-0.585
**BRD4_40**	7.602	8.226	-0.624
**BRD4_41**	7.678	7.704	-0.026
**BRD4_42**	8.009	7.756	0.253
**BRD4_43**	7.553	7.771	-0.218
**BRD4_44**	7.886	8.441	-0.555
**BRD4_45**	7.959	8.233	-0.274

3-D QSAR model building.

The conformation analysis is made by Balloon on the platform www.3d-qsar.com. In order to find the conformation of each molecule which has the lowest energy, it was performed a conformation analysis with at least 70 conformation for each molecule (Figure SI14).

Once the conformational analysis is performed, it needs to perform a molecular alignment. In the Ligand-Based approach, the alignment rules can be multiple and, in this work, it was used “lowest_LogP” rule. Therefore, the reference molecule is BRD4_27 (Table SI21).

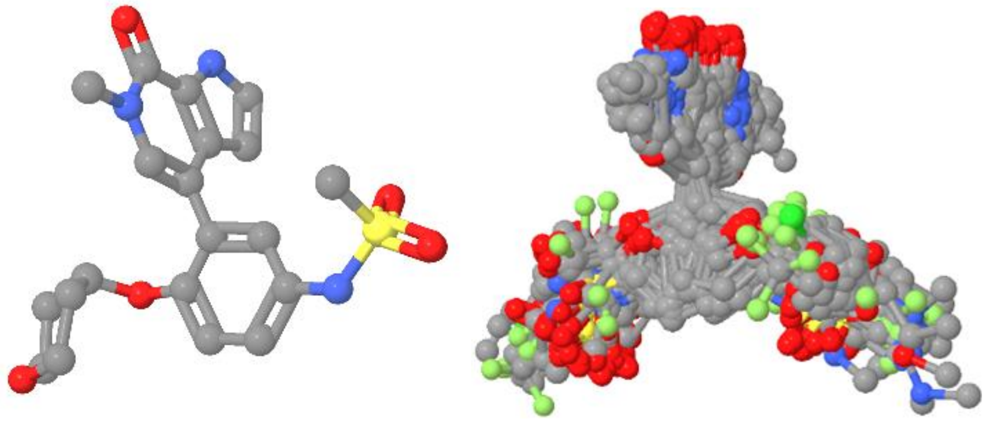

A B

Figure SI14. BRD4 alignment rules. (A) example of conformation obtained for the compound BRD4_27. (B) Training set aligned on the template molecule BRD4_27.

The best probe atom with best energy interaction results to be Hydrogen (H). It is found to be with an Electrostatic and Steric q^2 0.548, a Fitting r^2 of 0.886, a SDEC of 0.246 and a SDEP of 0.489, considering 4 optimal numbers of principal component (ONPC). These results were obtained by optimizing parameters by imposing a Grid spacing of 2.2 Å, a Grid extension of 5 Å, a Minimum sigma of 0.05 and cutoff energy of 25 kcal/mol (Table SI22). LSO and LOO validation was performed in order to assess the model stability. In addition, a Y-scrambling (Y_S) validation was made to verify if the model was affected by fortuitous correlation and the feedback was positive (Table SI23 and Figure SI15).

Table SI21. BRD_4 3-D QSAR coefficients obtained for the different alignment rules.

Alignment Rules	Probe Atom	q^2 STE	q^2 ELE	q^2 BOTH	Max ONPC	Grid Spacing	Grid Extension	CutOff	Min. Sigma	CV Type
**Least Active**	C.3	0.104	0.032	0.14	8	1.0	5	30.0	0.05	LOO
**Most Active**	H	0.210 (3)	0.348 (5)	0.369 (4)	8	1.0	5	30.0	0.05	LOO
**Heaviest**	C.3	-0.008 (1)	0.008 (1)	0.165 (3)	8	1.0	5	30.0	0.05	LOO
**Longest**	C.3	0.295 (3)	0.127 (3)	0.334 (3)	8	1.0	5	30.0	0.05	LOO
**Most Flexible**	C.3	0.425 (3)	0.211 (2)	0.392 (3)	8	1.0	5	30.0	0.05	LOO
**Most Rigid**	C.3	0.425 (3)	0.211 (2)	0.392 (3)	8	1.0	5	30.0	0.05	LOO
**Least Polar**	H	0.113 (4)	0.002 (1)	0.207 (5)	8	1.0	5	30.0	0.05	LOO
**Most Polar**	H	0.173 (7)	0.085 (3)	0.455 (3)	8	1.0	5	30.0	0.05	LOO
**Highest MR**	C.3	0.195 (2)	-0.003 (1)	0.283 (5)	8	1.0	5	30.0	0.05	LOO
**Lowest MR**	H	0.113 (4)	0.002 (1)	0.207 (5)	8	1.0	5	30.0	0.05	LOO
**Highest HA**	H	0.173 (7)	0.085 (3)	0.455 (3)	8	1.0	5	30.0	0.05	LOO
**Lowest HA**	H	-0.032 (8)	-0.004 (1)	0.244 (6)	8	1.0	5	30.0	0.05	LOO
**Highest HD**	C.3	0.182 (8)	0.158 (6)	0.298 (4)	8	1.0	5	30.0	0.05	LOO
**Lowest HD**	H	0.113 (4)	0.002 (1)	0.207 (5)	8	1.0	5	30.0	0.05	LOO
**Highest LogP**	C.3	0.170 (1)	0.003 (1)	0.178 (4)	8	1.0	5	30.0	0.05	LOO
**Lowest LogP**	H	0.274 (2)	0.364 (4)	0.524 (4)	8	1.0	5	30.0	0.05	LOO

For each alignment, a construction model was tested with the same option value for Grid spacing, Grid extension, CutOff, Min. Sigma and CV Type, changing only the probe atom to find the best result indicating in which type of alignment start building the model. The best model is highlighted in yellow

Table SI22. BRD_4 3-D QSAR models optimization by varying the pretreatment parameters on the best alignment rule.

Probe Atom	q^2 STE	q^2 ELE	q^2 BOTH	ONPC	Grid Spacing	Grid Extension	CutOff	Min. Sigma	CV Type	r^2	SDEC	SDEP	r^2 YS	q^2 YS	SDEP YS
C.3	0.289(2)	0.364(4)	0.515(4)	8	1.0	5	30.0	0.05	LOO	/	/	/	/	/	/
C.2	0.289(2)	0.364(4)	0.515(4)	8	1.0	5	30.0	0.05	LOO	/	/	/	/	/	/
C.cat	0.289(2)	0.364(4)	0.515(4)	8	1.0	5	30.0	0.05	LOO	/	/	/	/	/	/
H	0.274(2)	0.364(4)	0.524(4)	8	1.0	5	30.0	0.05	LOO	/	/	/	/	/	/
N.3	0.282(2)	0.364(4)	0.522(4)	8	1.0	5	30.0	0.05	LOO	/	/	/	/	/	/
O.3	0.282(2)	0.364(4)	0.522(4)	8	1.0	5	30.0	0.05	LOO	/	/	/	/	/	/
H	0.289(2)	0.323(4)	0.527(4)	8	1.1	5	30.0	0.05	LOO	/	/	/	/	/	/
H	0.297(2)	0.376(5)	0.519(4)	8	1.2	5	30.0	0.05	LOO	/	/	/	/	/	/
H	0.331(7)	0.160(4)	0.473(4)	8	1.3	5	30.0	0.05	LOO	/	/	/	/	/	/
H	0.257(1)	0.326(5)	0.501(4)	8	1.4	5	30.0	0.05	LOO	/	/	/	/	/	/
H	0.273(2)	0.413(5)	0.523(4)	8	1.5	5	30.0	0.05	LOO	/	/	/	/	/	/
H	0.301(2)	0.017(2)	0.436(4)	8	1.6	5	30.0	0.05	LOO	/	/	/	/	/	/
H	0.311(2)	0.273(5)	0.476(4)	8	1.7	5	30.0	0.05	LOO	/	/	/	/	/	/
H	0.324(7)	0.155(5)	0.519(4)	8	1.8	5	30.0	0.05	LOO	/	/	/	/	/	/
H	0.234(2)	0.105(4)	0.494(4)	8	1.9	5	30.0	0.05	LOO	/	/	/	/	/	/
H	0.217(2)	0.302(7)	0.492(4)	8	2.0	5	30.0	0.05	LOO	/	/	/	/	/	/
H	0.276(2)	0.267(8)	0.481(3)	8	2.1	5	30.0	0.05	LOO	/	/	/	/	/	/
H	0.312(1)	0.064(2)	0.539(4)	8	2.2	5	30.0	0.05	LOO	/	/	/	/	/	/
H	0.300(2)	0.090(4)	0.459(3)	8	2.3	5	30.0	0.05	LOO	/	/	/	/	/	/
H	0.312(2)	0.210(5)	0.513(5)	8	2.4	5	30.0	0.05	LOO	/	/	/	/	/	/
H	0.295(1)	0.005(1)	0.350(3)	8	2.5	5	30.0	0.05	LOO	/	/	/	/	/	/
H	0.200(2)	0.001(1)	0.271(3)	8	2.6	5	30.0	0.05	LOO	/	/	/	/	/	/
H	0.078(1)	0.015(1)	0.275(4)	8	2.7	5	30.0	0.05	LOO	/	/	/	/	/	/
H	0.131(2)	-0.001(1)	0.455(3)	8	2.8	5	30.0	0.05	LOO	/	/	/	/	/	/
H	0.150(2)	0.003(4)	0.346(5)	8	2.9	5	30.0	0.05	LOO	/	/	/	/	/	/
H	0.165(2)	-0.002(1)	0.194(4)	8	3.0	5	30.0	0.05	LOO	/	/	/	/	/	/
H	0.283(1)	0.072(2)	0.482(4)	8	2.2	5	30.0	0.05	LOO	/	/	/	/	/	/
H	0.308(2)	0.100(5)	0.461(4)	8	2.2	6	30.0	0.05	LOO	/	/	/	/	/	/
H	0.261(3)	0.049(4)	0.360(3)	8	2.2	7	30.0	0.05	LOO	/	/	/	/	/	/
H	0.207(3)	0.003(1)	0.367(3)	8	2.2	8	30.0	0.05	LOO	/	/	/	/	/	/
H	0.207(2)	0.215(5)	0.463(4)	8	2.2	9	30.0	0.05	LOO	/	/	/	/	/	/
H	0.293(1)	0.080(4)	0.532(4)	8	2.2	10	20.0	0.05	LOO	/	/	/	/	/	/
H	0.304(1)	0.045(2)	0.544(4)	8	2.2	5	25.0	0.05	LOO	0,886	0,246	0,491	/	/	/
H	0.312(1)	0.078(2)	0.538(4)	8	2.2	5	35.0	0.05	LOO	/	/	/	/	/	/
H	0.313(1)	0.081(2)	0.540(5)	8	2.2	5	40.0	0.05	LOO	/	/	/	/	/	/
H	0.304(1)	0.045(2)	0.544(4)	8	2.2	5	25.0	0.10	LOO	/	/	/	/	/	/
H	0.304(1)	0.045(2)	0.544(4)	8	2.2	5	25.0	0.15	LOO	/	/	/	/	/	/
H	0.304(1)	0.045(2)	0.544(4)	8	2.2	5	25.0	0.20	LOO	/	/	/	/	/	/
H	0.304(1)	0.045(2)	0.544(4)	8	2.2	5	25.0	0.25	LOO	/	/	/	/	/	/
H	0.304(1)	0.045(2)	0.544(4)	8	2.2	5	25.0	0.30	LOO	/	/	/	/	/	/
H	0.304(1)	0.045(2)	0.544(4)	8	2.2	5	25.0	0.35	LOO	/	/	/	/	/	/
H	0.304(1)	0.045(2)	0.544(4)	8	2.2	5	25.0	0.40	LOO	/	/	/	/	/	/

H	0.304(1)	0.045(2)	0.544(4)	8	2.2	5	25.0	0.45	LOO	/	/	/	/	/	/
H	0.304(1)	0.045(2)	0.544(4)	8	2.2	5	25.0	0.50	LOO	/	/	/	/	/	/
H	0.304(1)	0.045(2)	0.544(4)	8	2.2	5	25.0	0.55	LOO	/	/	/	/	/	/
H	0.304(1)	0.046(2)	0.544(4)	8	2.2	5	25.0	0.60	LOO	/	/	/	/	/	/
H	0.304(1)	0.046(2)	0.544(4)	8	2.2	5	25.0	0.65	LOO	/	/	/	/	/	/
H	0.304(1)	0.047(2)	0.544(4)	8	2.2	5	25.0	0.70	LOO	/	/	/	/	/	/
H	0.304(1)	0.047(2)	0.544(4)	8	2.2	5	25.0	0.75	LOO	/	/	/	/	/	/
H	0.304(1)	0.047(2)	0.544(4)	8	2.2	5	25.0	0.80	LOO	/	/	/	/	/	/
H	0.304(1)	0.046(2)	0.544(4)	8	2.2	5	25.0	0.85	LOO	/	/	/	/	/	/
H	0.304(1)	0.045(2)	0.544(4)	8	2.2	5	25.0	0.90	LOO	/	/	/	/	/	/
H	0.305(1)	0.045(2)	0.544(4)	8	2.2	5	25.0	0.95	LOO	/	/	/	/	/	/
H	0.305(1)	0.045(2)	0.544(4)	8	2.2	5	25.0	1.00	LOO	/	/	/	/	/	/
H	0.305(1)	0.044(2)	0.544(4)	8	2.2	5	25.0	1.05	LOO	/	/	/	/	/	/
H	0.305(1)	0.043(2)	0.544(4)	8	2.2	5	25.0	1.10	LOO	/	/	/	/	/	/
H	0.305(1)	0.041(2)	0.544(4)	8	2.2	5	25.0	1.15	LOO	/	/	/	/	/	/
H	0.305(1)	0.039(4)	0.544(4)	8	2.2	5	25.0	1.20	LOO	/	/	/	/	/	/
H	0.305(1)	0.040(1)	0.544(4)	8	2.2	5	25.0	1.25	LOO	/	/	/	/	/	/
H	0.305(1)	0.042(1)	0.544(4)	8	2.2	5	25.0	1.30	LOO	/	/	/	/	/	/
H	0.305(1)	0.043(1)	0.544(4)	8	2.2	5	25.0	1.35	LOO	/	/	/	/	/	/
H	0.305(1)	0.045(1)	0.544(4)	8	2.2	5	25.0	1.40	LOO	/	/	/	/	/	/
H	0.305(1)	0.048(1)	0.543(4)	8	2.2	5	25.0	1.45	LOO	/	/	/	/	/	/
H	0.305(1)	0.048(1)	0.543(4)	8	2.2	5	25.0	1.50	LOO	/	/	/	/	/	/
H	0.305(1)	0.050(1)	0.543(4)	8	2.2	5	25.0	1.55	LOO	/	/	/	/	/	/
H	0.305(1)	0.053(1)	0.543(4)	8	2.2	5	25.0	1.60	LOO	/	/	/	/	/	/
H	0.305(1)	0.056(1)	0.543(4)	8	2.2	5	25.0	1.65	LOO	/	/	/	/	/	/
H	0.305(1)	0.058(1)	0.542(4)	8	2.2	5	25.0	1.70	LOO	/	/	/	/	/	/
H	0.305(1)	0.060(1)	0.541(4)	8	2.2	5	25.0	1.75	LOO	/	/	/	/	/	/
H	0.305(1)	0.061(1)	0.540(4)	8	2.2	5	25.0	1.80	LOO	/	/	/	/	/	/
H	0.305(1)	0.063(1)	0.539(4)	8	2.2	5	25.0	1.85	LOO	/	/	/	/	/	/
H	0.305(1)	0.064(1)	0.540(4)	8	2.2	5	25.0	1.90	LOO	/	/	/	/	/	/
H	0.305(1)	0.065(1)	0.539(4)	8	2.2	5	25.0	1.95	LOO	/	/	/	/	/	/
H	0.305(1)	0.067(1)	0.538(4)	8	2.2	5	25.0	2.00	LOO	/	/	/	/	/	/
H	0.313(2)	0.087(2)	0.548(4)	8	2.2	5	25.0	0.05	LSO	0,886	0,246	0,548	0,745	-0,369	0,845

The best models (LOO and LSO) are highlighted in yellow.

Table SI23. Selected 3-D QSAR model metrics for BRD4

Fields	ONPC[a]	r^2	SDEC[b]	q^2		SDEP[c]		r^2_{YS}	q^2_{YS}	
				LOO[d]	LSO[e]	LOO	LSO		LOO	LSO
**Steric**	2	0.697	0.400	0.282	0.313	0.616	0.603	0.551	-0.648	-0.393
**Electrostatic**	2	0.312	0.603	0.045	0.087	0.711	0.695	0.224	-0.397	-0.233
**Steric/Electrostatic**	4	0.886	0.246	0.544	0.548	0.491	0.489	0.745	-0.676	-0.369

Optimal Settings

**Probe Atom**	H
**Grid Spacing**	2.2
**Grid Extension**	5
**Minimum Sigma**	0.05
**Max/Min Energy of Cutoff Value**	25

[a] ONPC: Optimal Number of Principal Components

[b] SDEP: Cross-validated Standard Deviation Error Prediction

[c] SDEC: Standard Deviation Error Calculation

[d] LOO: Leave-One-Out

[e] LSO: Leave-Some-Out

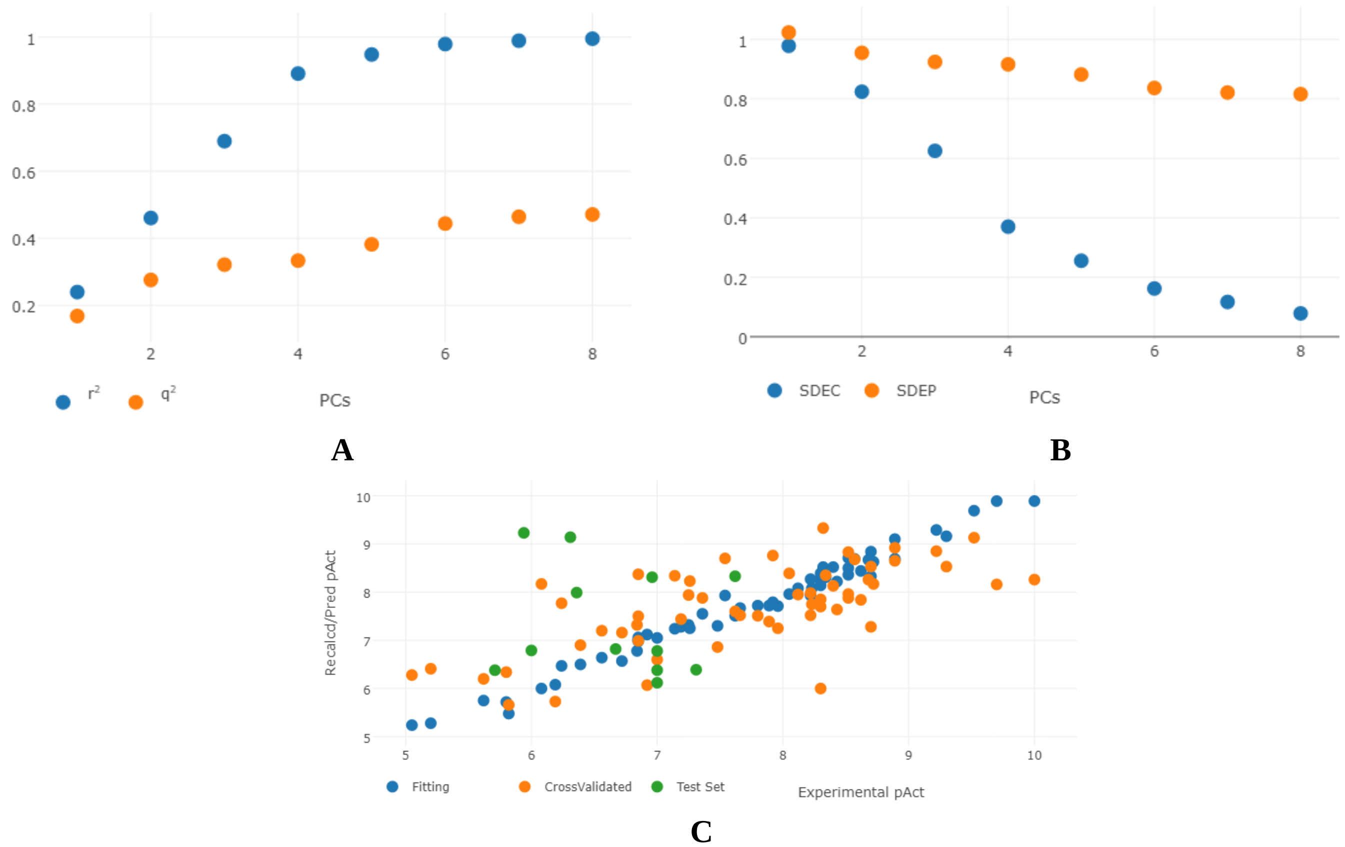

Figure SI15. Plots for IRAK4 dataset. **(A)** r^2 and q^2 versus the number of PCs; **(B)** SDEC and SDEP versus the number of PCs; **(C)** Recalculated and predicted versus the experimental activities.

Final model graphical inspection.

3-D QSAR contour maps were generated using the Coeff x Mean option, using both the most active and the least active model, superimposing them. In this way, steric and electrostatic CoMFA like maps were obtained and studied. The CAA associated with positive and negative steric is represented by green and yellow polyhedra, as suggested in the original article CoMFA [30] (panels A and B of Figure SI16).

In yellow (Figure SI16A) we see the negative steric fields, where a further steric hindrance is not recommended. In fact, they could lead to less activity.

In green (Figure SI16B), therefore, we see areas with positive steric fields, where it is therefore advisable to add substituents to increase activity.

In particular, it is observable green polyhedron around the fluorine atoms (in green) suggests that a slight increase in steric hindrance in that position could lead to more active compounds. But even a replacement with a larger group instead of the methyl group of the 6-methyl-1H-pyrrolo [2,3-c] pyridine-7-one group or in the sulfur position of the ethanesulfonamide group could lead to better structures. (Figure SI16A). While, instead, as regards the negative steric component (in yellow), it is necessary to avoid substitutions on the carbonyl group of 6-methyl-1H-pyrrole [2,3 - c] pyridine-7-one (Figure SI16B).

In positive electrostatic contour maps (blue polyhedra) the attractive forces are favored around the 6-methyl-1H-pyrrole [2,3-c] pyridine-7-one group (Figure SI16C), while the repulsive forces (red polyhedra) are favored close to the second aromatic diphenyl ether ring (Figure SI16D).

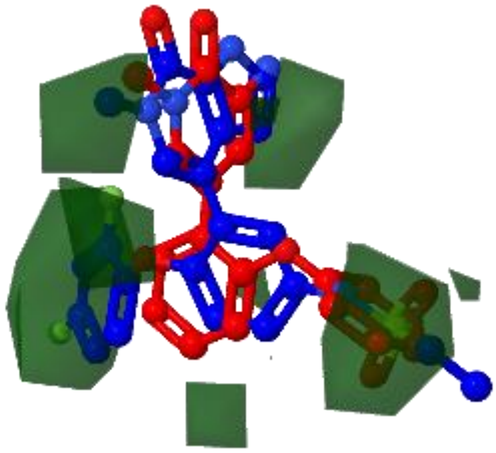

A

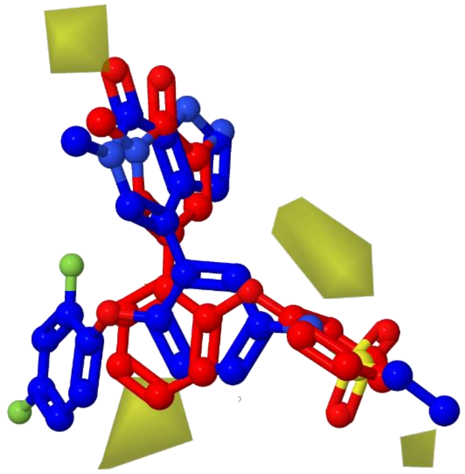

B

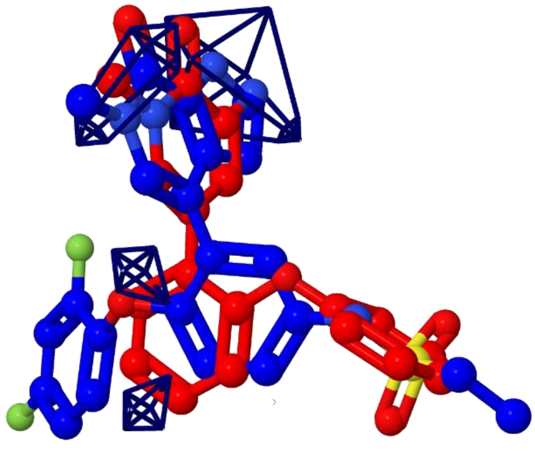

C

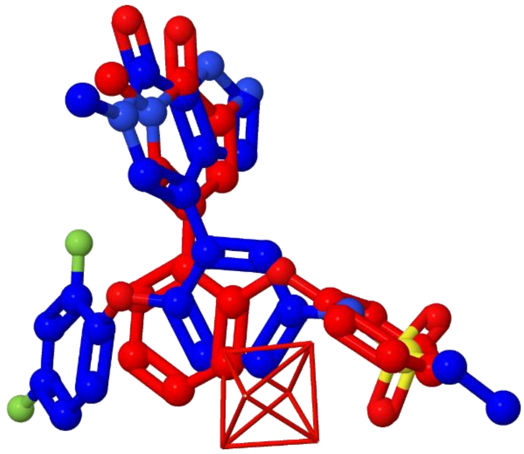

D

Figure SI16. A) Least (ID_03 in red) and most (ID_21 in blue) active molecule's positive (in green) and negative (in orange) steric Coeff x Mean. B) Least (in red) and most (in blue) active molecule's positive (in green) and negative (in orange) electrostatic Coeff x Mean.

Test set composition

Model's external validation was performed with the molecules of the test set and the following table summarized the difference between the experimental activities and those foreseen. The molecules were obtained by randomly selecting 12 compounds from the ChEMBL database (https://www.ebi.ac.uk/chembl/) and subjecting them to all the procedure and getting the predicted activities (Table SI24). It must be noted however that, inasmuch as ChEMBL is a record of <u>all</u> published structures having <u>any</u> reported biological potency, the very poor accuracy of these predictions for these random structural choices is only to be expected.

Table SI24. Test set experimental and predicted activity

ID	Experimental	Predicted	Error
**BRD4_46**	6.013	6.697	-0.684
**BRD4_47**	5.770	7.639	-1.869
**BRD4_48**	6.046	7.746	-1.700
**BRD4_49**	6.854	8.073	-1.219
**BRD4_50**	5.959	8.225	-2.266
**BRD4_51**	5.721	8.453	-2.732
**BRD4_52**	6.347	6.851	-0.504
**BRD4_53**	7.237	8.524	-1.287

**BRD4_54**	8.086	7.093	-0.993
**BRD4_55**	8.357	8.328	-0.029
**BRD4_56**	8.252	8.385	-0.133
**BRD4_57**	8.585	7.782	0.803

List of full tutorial on the www.3d-qsar.com four web applications elaborated by the four students

Py-MolEdit: https://www.3d-qsar.com/forum/attachment/3/

Py-ConfSearch: https://www.3d-qsar.com/forum/attachment/4/

Py-Align: https://www.3d-qsar.com/forum/attachment/5/

Py-CoMFA: https://www.3d-qsar.com/forum/attachment/4/
